# Using random walks to identify cancer-associated modules in expression data

**DOI:** 10.1186/1756-0381-6-17

**Published:** 2013-10-15

**Authors:** Deanna Petrochilos, Ali Shojaie, John Gennari, Neil Abernethy

**Affiliations:** 1 Biomedical and Health Informatics, Dept of Biomedical Informatics and Medical Education, University of Washington, Box 357240, 1959 NE Pacific Street, HSB I-264, Seattle, WA 98195-7240, USA; 2 Department of Biostatistics, University of Washington, Box 357232, F-650 Health Sciences Bldg, Seattle, WA, USA

**Keywords:** Network analysis, Cancer, Modules, Graph theory, Interactions, Random walk, Walktrap

## Abstract

**Background:**

The etiology of cancer involves a complex series of genetic and environmental conditions. To better represent and study the intricate genetics of cancer onset and progression, we construct a network of biological interactions to search for groups of genes that compose cancer-related modules. Three cancer expression datasets are investigated to prioritize genes and interactions associated with cancer outcomes. Using a graph-based approach to search for communities of phenotype-related genes in microarray data, we find modules of genes associated with cancer phenotypes in a weighted interaction network.

**Results:**

We implement *Walktrap*, a random-walk-based community detection algorithm, to identify biological modules predisposing to tumor growth in 22 hepatocellular carcinoma samples (GSE14520), adenoma development in 32 colorectal cancer samples (GSE8671), and prognosis in 198 breast cancer patients (GSE7390). For each study, we find the best scoring partitions under a maximum cluster size of 200 nodes. Significant modules highlight groups of genes that are functionally related to cancer and show promise as therapeutic targets; these include interactions among transcription factors (*SPIB*, *RPS6KA2* and *RPS6KA6*), cell-cycle regulatory genes (*BRSK1*, *WEE1* and *CDC25C*), modulators of the cell-cycle and proliferation (*CBLC* and *IRS2*) and genes that regulate and participate in the map-kinase pathway (*MAPK9, DUSP1, DUSP9, RIPK2*). To assess the performance of *Walktrap* to find genomic modules (*Walktrap-GM*), we evaluate our results against other tools recently developed to discover disease modules in biological networks. Compared with other highly cited module-finding tools, *jActiveModules* and *Matisse, Walktrap-GM* shows strong performance in the discovery of modules enriched with known cancer genes.

**Conclusions:**

These results demonstrate that the *Walktrap-GM* algorithm identifies modules significantly enriched with cancer genes, their joint effects and promising candidate genes. The approach performs well when evaluated against similar tools and smaller overall module size allows for more specific functional annotation and facilitates the interpretation of these modules.

## Background

Cancer biology involves an intricate series of genetic and environmental interactions that act in concert to influence the onset and progression of disease. The complex nature of this information motivates the search for analytical tools that can model these interactions to examine associations between gene interactions and cancer. Network-based studies facilitate these genotype-phenotype investigations by integrating evidence of biological interactions from high throughput experiments, the literature, and a growing number of online databases, to improve the prioritization of disease genes and their interactions.

Gene set enrichment approaches leverage genomic interaction and pathway information to enable the study of putative genes in the context of their biological processes. Gene Set Enrichment Analysis (GSEA) [1] is a computational method that considers a priori defined gene sets to investigate expression data for significantly enriched sets of genes or pathways. GSEA focuses on the significance of groups of interacting genes rather than individual genes; and variations have been developed to improve statistical validity [2-5] and to use more granular methods to study pathway activity [6-9]. Such approaches allow interpretation of significant genes in the setting of their pathway interactions and functional relevance; however, they are limited in their ability to search for significantly expressed genes that form a small component of large pathways or interact across multiple gene sets.

Network analyses provide a framework to study genes in the context of interactions derived from multiple data sources and integrated as a global interactome. Several studies evaluate the topology of disease genes in global interaction networks and have found that related disease genes are more likely to interact, and that cancer genes are associated with high network centrality [10-12]. Building on the hypothesis that neighboring genes within an interaction network share a common biological function, other network studies seed known disease genes in functional networks combining evidence from the literature, functional annotation, genomic distances, or genetic variation data (i.e., GWAS, SNP, eQTL), to search for nearby putative genes [13-15]. Related work integrates experimental data in the interaction network, for example, significant genes from regulatory or proteomic experiments, to discover candidate genes given their proximity to query genes [16-18].

Further graph-based approaches search for densely connected communities within interaction networks, using the structure of the network and weights derived from experimental data to find modules significantly associated with phenotypes of interest. Dittrich and colleagues [19] implement a Steiner tree to find parsimonious subnetworks of cancer-related genes in microarray studies. The tree is an optimally connected subgraph spanning an interactome weighted by expression data. Ideker et al. and Chuang et al. [20,21] apply a simulated annealing algorithm to identify significant subgraphs associated with cancer in a protein interaction network. The algorithm initiates module generation with seed genes and iteratively adds nearby proteins with significant p-values to the subnetwork, until an optimal score is reached reflecting differential activity of the module in the expression data. Ulitsky and Shamir [22] use a seed-based clustering algorithm to discover significant modules in yeast and human cell cycle data. They provide multiple heuristics to generate seeds in the network and build modules based on similarity in expression values. These studies conclude that mining for dense subgraphs of significant genes within interaction networks can reveal modules of genomic interactions that are functionally relevant to specific phenotypes of interest.

Among graph-based algorithms used to study genomic data, random walks have shown strong performance in the prioritization of disease genes and when evaluated against other graph-clustering algorithms used to partition complex networks [23-25]. Transition probabilities generated by the random walk are drawn upon to calculate distances between network nodes that can be used to prioritize genes or as metrics for clustering algorithms. Kholer et al. [14] apply a random walk in a functional interaction network to identify novel disease genes by their proximity to known disease genes based on genome mapping, eQTL and interaction data. They conclude that random walks outperform other distance-based methods in prioritizing related disease genes, and similar approaches have been applied in genome-phenome networks [26,27]. Tu et al. [28] employ a heuristic random walk in an integrated network to find regulatory modules in gene expression data, identifying the most likely path from quantitative trait loci to a candidate gene. The Markov Clustering (MCL) algorithm, based on random walks, has been applied to cluster proteomic and expression data using similar expression profiles [29], and to search for gene signatures in cancer expression data [30]. Komurov et al. [23,31] implement a random walk algorithm to prioritize cancer genes and hierarchically cluster expression data. These studies show the random walk is well-adapted to genome studies in interaction networks and can be used to define distances between nodes that reflect correlation or relevance of interaction between nodes.

The implementation of random walks varies based on optimization strategies, greedy-search heuristics and the calculation of distance metrics. We use a random-walk algorithm, *Walktrap*[32], that is optimized for large networks and integrates a community search using distances derived from transition probabilities. We develop a scoring method to rank significant modules, and configure the algorithm to improve the search for informative modules by including a series of stopping criteria in the merge process, using modularity, module size and maximum module score to guide clustering. The random walk algorithm adapted to genomic modules (*Walktrap-GM*), is applied to guide a semi-supervised search for cancer-related modules in an expression-weighted interactome. *Walktrap-GM* demonstrates strong performance compared with similar tools developed to identify subnetworks of disease genes in interaction networks and highlights the potential role of candidate genes and their interactions in cancer.

## Methods

**Figure 1:**
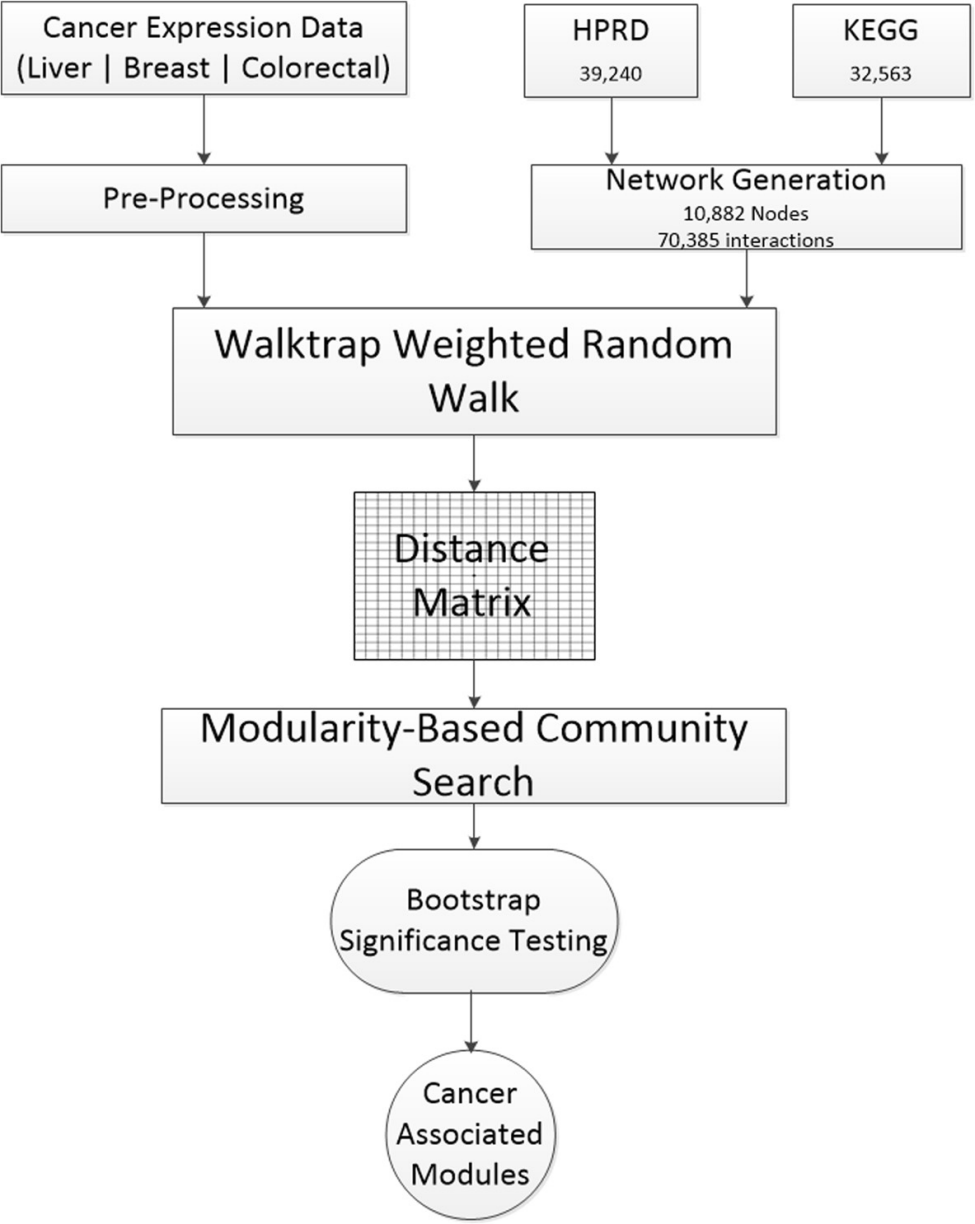
**Flow diagram of network-based expression analysis.** Three cancer datasets from GEO and interactions from HPRD and KEGG are integrated in a weighted interaction network. The *Walktrap* random walk builds modules based on transition probabilities generated from the random walk process. The modules are assessed for their significance compared to a random distribution of differential expression values per module.

We employ a graph-based random walk algorithm in an integrated interaction network to mine expression data for modules of genes associated with cancer outcomes. First, metabolic, signaling, and protein interactions from the Kyoto Encyclopedia of Genes and Genomes (KEGG) [33] and the Human Protein Reaction Database (HPRD) [34] are used to construct a global network of biological interactions. Edge weights are derived from expression data from three public datasets with multiple cancer outcomes: breast cancer, hepatocellular carcinoma and colorectal adenoma. We apply a random walk algorithm to these networks to discover modules of closely interconnected genes and build communities using distances derived from the random walk process. Finally, a score is calculated for each community and modules are ranked by significance. These methods are summarized in Figure 1.

### Gene expression data

Three cancer datasets were downloaded from the Gene Expression Omnibus (GEO) [35] covering onset of breast cancer prognosis (BC), hepatocellular carcinoma (HCC), and adenoma development in colorectal cancer (CCA). Data were selected to represent different stages of cancer onset and development, by the availability of paired samples comparing normal and adjacent tissues, and detailed prognosis data. We include three recent, large case–control studies from expression studies generated by common platforms, Affymetrix U133A and U133A 2.0 arrays. GSE14520 is a study of hepatocellular carcinoma conducted by Roessler et al. [36], consisting of 22 paired tumor and non-tumor expression profiles using the Affymetrix HG-U133A 2.0 array. Desmedt et al. [37] published an expression dataset consisting of 198 samples to independently validate a 76-gene prognostic breast cancer signature as part of the TRANSBIG project (GSE7390). A total of 198 profiles from lymph node-negative patients (N-) were analyzed on the Affymetrix HG-U133A array, and each profile was associated with the Adjuvant!Online clinical risk index, identifying patients at high risk for distant metastasis (good = 47, poor = 151). Sebates-Bellver [38] obtained tissue from sporadic colonic adenomas and normal mucosa of 32 colonoscopy patients and analyzed expression profiles using Affymetrix HG-U133A 2.0 arrays (GSE8671). Normal tissue was compared to colonic adenoma cancer precursors. These data are summarized in Table 1.

**Table 1:** Description of cancer expression data

**GEO accession**	**Reference**	**Clinical outcome**	**# of samples**	**Controls**
**GSE14520**	Roessler 2010	Hepatocellular carcinoma tumors (HCC)	22 Hepatocellular tumors	22 Paired non-tumor
**GSE7390**	Desmedt 2007	Risk of early distant breast cancer metastasis (BC)	198 Breast tumors from lymph-node negative patients	Favorable prognosis (good = 47, poor = 151)
**GSE8671**	Sebates-Bellver 2007	Colorectal cancer adenomas (CCA)	32 Paired sporadic adenoma	32 Paired normal

We calculate normalized, log-transformed fold-change values and p-values comparing paired normal versus disease tissue in HCC and CCA, and high-risk versus low-risk samples in BC. Log odds of differential expression were calculated by empirical Bayes and corresponding p-values were corrected for multiple testing using the Benjamini and Hochberg false discovery rate [39]. Processing of expression values and phenotype data and differential statistical analyses were performed in R using the GEOquery [40] and limma [41,42] packages in Bioconductor [43].

### Network construction

The interactome for this study was built by extracting human interactions from KEGG and HPRD. KEGG relations were parsed from KGML files, representing 32,563 unique interactions. Metabolic reactions were defined as a relation between two neighboring enzymes that share a common metabolite; signaling reactions were defined as two genes that participate in a signaling cascade where both genes share a reaction event. A total of 39,240 protein-protein interactions were downloaded from HPRD. Duplicate nodes and edges were removed and provenance of each interaction was saved as an edge attribute. The resulting global interaction network consisted of 10,882 nodes and 70,385 interactions. The largest connected cluster of unique pairwise interactions consisting of 10,642 nodes and 62,407 interactions was extracted for further analysis.

Global statistics are summarized for the network: the network diameter is 15; graph density is 0.0011; average node degree is 11.72; average node betweenness, the number of shortest paths via a node or edge are 16723 and 3759, respectively; closeness, or the inverse of the number of nodes in the shortest path from one node to all other nodes in the network, is .2454; the average shortest path length is 4.1281; and global clustering coefficient is 0.1314. Small average path length between network nodes and high betweenness characterize the small world property of the network such that all nodes are generally reachable by all other nodes in the network by relatively few steps.

### Weights and significance scoring

For each interaction network corresponding to HCC, CCA or BC data, edge weights are estimated as a function of differential expression. Each node is mapped to an HGNC gene symbol and the activity of that node is determined by the absolute value of the fold-change estimate for that symbol. Where multiple probes are associated with a gene symbol, we choose the node with maximum differential expression. This approach was chosen rather than using p-values, as fold-change measures were more robust weight factors with a more discrete range of values and stable dispersion. Absolute fold change was used in lieu of correlation values to support a semi-supervised analysis of the association between the cumulative activity of all nodes in the module and the outcome variables versus unsupervised clustering.

Weights are applied to each edge by calculating the square of the mean of the two adjacent nodes of the edge, $\left(\frac{\left|\text{FC}1\right|+\left|\text{FC}2\right|}{2}\right)$. The average weighting scheme was considered best suited to the random walk approach as it allows for more descriptive probabilities than weighting schemes that use for example, maximum or minimum values. Further, this weighting scheme improves community cohesiveness in settings where and indirect interaction may exhibit significant differential expression, but the intermediate interaction is non-significant.

The cumulative activity of a module is a squared transformation of the average weighted expression for all nodes in the module; where the weight of a given node is the maximum fold change of probes corresponding to its gene symbol. We evaluate the significance of the magnitude of expression for modules greater than three nodes by comparing the cumulative activity of the module against a random distribution. The random distribution is a sample 5000 permutations of cumulative activity estimates per module size with *n* nodes, and each permutation is generated by a random sampling of *n* fold-change values. The module score is a test statistic comparing the cumulative activity of a module against the bootstrap distribution (*(μ*_*0*_^*2*^*-μ*_*1*_^*2*^*/)σ*_err_^2^), and is used to rank high-scoring modules.

### Community analysis

Among graph-based approaches, the random walk on graphs performs well in defining distances between nodes and has been applied to find communities in networks. We utilize a random walk algorithm, *Walktrap,* developed by Pons and Latapy [32] and implemented in iGraph, [44] to simulate a random walk in the interaction network. The random walk, compared to other popular hierarchical or seed clustering methods, utilizes the structure of the network to build distance metrics, and *Walktrap* optimizes the community search using the graph-theoretic concept of modularity. The algorithm has shown high efficiency and accuracy in revealing community structure in large networks [45]. The complexity of the algorithm is generally *Ο*(*mH* log *n*), and *Ο*(*n*^3^) in sparse matrices [32] and run time statistics using the *Walktrap* are summarized in Additional file 1. Further, in benchmark testing, we found the random walk to be computationally more efficient than using edge-betweenness, spectral methods, or spanning trees, to detect communities.

The algorithm begins with graph *G* and its associated adjacency matrix *A.* In the weighted network, $A_{\text{ij}}\in R$ if *i* and *j* are connected in *G*, and *A*_*ij*_ = 0 otherwise. The random walk process starts at a vertex *i*, and at each time point in the walk of length *t*, a random step is taken to an adjacent node *j*. Here we set *t* to 3. The transition probability at each step is $P_{\mathrm{ij}}=\frac{A_{\mathrm{ij}}}{d\left(i\right)}$ where *d*_*(i)*_ is the degree of vertex *i , d*(*i*) = ∑ _*j*_*A*_*ij*_. Transition probabilities define the transition matrix ***P*** of the random walk, and powers of ***P*** determine the probability ***P***^*t*^_*ij*_ that the walker will traverse from *i* to *j* over time *t*. Structural similarity between vertices and communities are calculated using probabilities ***P***^*t*^_*ij*_ to measure the distance between nodes. The distance between the two vertices *i* and *j*, *r*_*ij*_ is computed by:

(1) $$r_{\text{ij}}=\sqrt{\sum_{k=1}^{n}\frac{{\left(P_{\text{ik}}^{t}-P_{\text{jk}}^{t}\right)}^{2}}{d\left(k\right)}}$$

Similarly, the distance between two communities *C*_*1*_ and *C*_*2*_ is:

(2) $$r_{C_{1}C_{2}}=\sqrt{\sum_{k=1}^{n}\frac{{\left(P_{c_{1}k}^{t}-P_{c_{2}k}^{t}\right)}^{2}}{d\left(k\right)}}$$

Where $P_{\text{cjk}}^{t}$ measures the probability of traversing from a node in *C*_*j*_ to node *k* (*j* = 1,2). At each step in the merge algorithm, two communities are selected to be merged if the merge minimizes the mean *σ*_*k*_ of the squared distances between each vertex and its community:

(3) $$\sigma_{k}=\frac{1}{n}\sum_{C\in R_{k}}\sum_{i\in C}r_{\mathrm{iC}}^{2}$$

After the merge step, the decrease in squared distances Δ*σ* between the communities is found by:

(4) $$\Delta_{\sigma }\left(C_{1}-C_{2}\right)=\frac{1}{n}\frac{\left|C_{1}\right|\left|C_{2}\right|}{\left|C_{1}\right|+\left|C_{2}\right|}r_{C_{1}C_{2}}^{2}$$

The merge process continues until the modularity of the network is maximized. Modularity *Q* of partition *R* compares the fraction of edges *e*_*C*_ inside the community *C* and the fraction of edges bound to the community, *a*_*C*_ :

(5) $$Q\left(R\right)=\sum_{C\in P}e_{C}-a_{C}^{2}$$

Further background and details of the *Walktrap* implementation are provided in the original work [32].

To customize the algorithm to discover significant and interpretable cancer-associated modules in *Walktrap-GM*, we implement stopping criteria to search for the optimal number of merge steps. The merge process is complete when one of the following conditions is met: 1) maximum modularity, 2) maximum size, or 3) maximum module score (Section Weights and significance scoring). We chose a maximum size of 200 nodes as the upper bound to maintain interpretability of modules, as we tested a subset of larger maximum sizes between 200 and 500 that generally resulted in modules that were too general in their functional annotation and therefore not as informative.

### Functional annotation and overlap with GSEA

Functional annotation for significant modules is assessed using *ConsensusPathDB*[46]. For top-scoring modules, we queried the list of genes in the module using overrepresentation analysis. We query these genes for overrepresentation in curated pathways from KEGG, WikiPathways [47], PID [48], HumanCyc [49] and Reactome [50]. Parameters include a minimum overlap of two genes with our input gene list and the consensus pathway and the default significance threshold, p < = .01.

We analyze each cancer phenotype using GSEA to generate a list of enriched genes. Briefly, GSEA is a commonly applied method that searches for enrichment of highly expressed genes in curated or custom gene sets to identify differential activity of a priori defined gene sets in two phenotypes. The method ranks significant genes and calculates an enrichment score for each gene set based on a weighted Kolmogorov-Smirnov-like statistic. We apply GSEA using all canonical pathways (c3.cpv2.5) in the Molecular Signatures Database, with the following parameters: number of permutations = 1000, collapse to gene symbols = TRUE, and permutations by phenotype. We assess results comparing disease versus normal in HCC and CCA and high risk versus low risk in BC. Enriched gene sets are ranked by normalized enrichment score (NES).

Overlap of top-scoring modules with results with GSEA results was evaluated by cross-validating the top 10 GSEA gene sets ranked by NES with significantly overrepresented pathways in our modules (as determined by analysis of functional annotation using *ConsensusPathDB*). To evaluate the agreement of the annotation of top-scoring modules using *Walktrap-GM* and GSEA, we reviewed the annotation of top-scoring modules for overrepresentation of the top 10 gene sets ranked by NES. We report modules that show the highest overlap with these gene sets.

### Comparison with related graph-clustering platforms

The performance of *Walktrap-GM* is compared with two highly cited platforms developed to find network modules using gene expression data in interaction networks, *jActiveModules*[20] and *Matisse*[22]. *jActiveModules* initiates module generation with seed genes and builds a high scoring subgraph by iteratively evaluating the addition of neighboring nodes based on their p-values. The annealing algorithm uses a temperature parameter simulating a cooling factor that imposes a probability to the addition of nodes that do not improve the module score and this probability decreases with each iteration until the algorithm becomes greedy. An activity score for the modules is calculated based on significance values associated with the proteins in the subnetwork. *Matisse* applies a seed clustering algorithm that uses optimization of seed data and similarity across expression profiles to cluster nodes. The algorithm uses high-scoring sets of similarly expressed nodes as seed data and iteratively improves sets of seed genes by considering the addition of connected nodes and improvement in module score. Module scores are based on the aggregate differential activity of the subnetwork.

To evaluate the ability of these tools to identify cancer-related genes and interactions, a list of cancer-related genes was extracted from OMIM, using text string matching and manual curation. We queried 6995 gene references including all genes in the clusters assessed, for cancer-related terms. The resulting list consisted of 1239 cancer-associated genes (Additional file 2). Each matching record was reviewed to confirm that the gene was a tumor suppressor, oncogene, or shown to be otherwise significantly associated with cancer (i.e., by differential expression data, functional pathway analysis, genomic mapping or SNP studies). Approximately 5% of genes did not have corresponding records in OMIM and were labeled non-cancer due to lack of evidence.

Parameters set to execute *jActiveModules* were *regional scoring*, *adjust score for size*, *overlap* = 0, and *number of modules* =1000. Parameters set for *Matisse* were *beta* = .95, *min seed size* =2, *min module size = 2*, *max module size* =200, *search strategy = all neighbors*, and *no regulation priors*. *Walktrap* modules do not include overlapping nodes; *jActiveModules* was configured to not allow overlap, while the *Matisse* algorithm is designed to include overlap. To evaluate the significance of each module, genes in the interaction network were randomly sampled to generate 5000 random distributions of class labels for each module size. The performance of each platform is assessed by calculating a cancer-enrichment score for highly-ranked modules, or the significance of the number known cancer genes in each module compared to the random distribution. We also summarize the module size of significant and non-significant modules across platforms.

## Results and discussion

### Functional annotation

Functional annotation for significant modules is determined using *ConsensusPathDB*[46]. We query genes in the top-scoring modules for overrepresentation in curated pathways. Canonical cancer pathways and pathways associated with hallmarks of cancer are enriched in each cancer dataset: cell-cycle control, DNA replication/repair, cellular adhesion/migration, apoptosis, angiogenesis, evasion of the immune response and immortality. A summary of the statistics and representative pathways for the top-scoring modules is presented in Table 2. BC modules are highly enriched with cell cycle control, growth signaling, focal adhesion, and angiogenesis control genes. A number of BC modules are also annotated with progesterone, estrogen and steroid hormone signaling; and levels of these hormones are known to correlate with BC risk. In HCC, detoxifying pathways including cytochrome P450, UBR, HSD detoxifying pathways and fatty acid metabolism, are among the most enriched pathways. Inflammation and deregulation of liver-related detoxifying pathways are frequent markers of carcinogenic toxicity, oxidative stress, and tumorigenesis. Chronic inflammation and the immune response are associated with adenoma formation in the colon; and several related pathways are over-represented in CCA, including chemokine, cytokine, T-cell receptor, fatty acid metabolism, and intestinal immunity. *Wnt* signaling is a key pathway in early stages of colorectal cancer and is enriched in CCA modules. Amino acid synthesis and metabolism pathways, associated with stability of DNA replication and repair, are over-represented across all three cancer types, although most notably in HCC.

**Table 2:** Functional overview of top scoring modules

**Breast cancer**			
**ID**	**Score**	**Size**	**Key functional annotation**
**134**	**40.20**	**16**	DNA REPLICATION, ATR SIGNALING, CELL CYCLE, SYNTHESIS OF DNA, UNWINDING OF DNA
**82**	**27.77**	**32**	VEGF AND VEGFR SIGNALING, FOCAL ADHESION, CYTOKINE RECEPTOR INTERACTIONS, MTOR SIGNALING, PI3K CASCADE, ERBB SIGNALING, IRS SIGNALING, ANGIOGENESIS, FGFR SIGNALING, GLYPICAN1 NETWORK, SYNDECAN SIGNALING, IGF1 PATHWAY, ARF6 SIGNALING
**226**	**21.26**	**16**	NUCLEAR ESTROGEN RECEPTOR ALPHA NETWORK, REGULATION OF ANDROGEN RECEPTOR
**224**	**19.08**	**27**	METABOLISM OF NUCLEOTIDES, DNA REPLICATION, APOPTOSIS PATHWAY, ARF6 PATHWAY, CAM PATHWAY, TELOMERES EXTENSTION, PLC-G1 SIGNALING, GLUCAGON SIGNALING, C-MYC TRANSCRIPTION, GNRH SIGNALING, ERBB2 SIGNALING, EGFR SIGNALING IN CANCER
**79**	**16.08**	**24**	JAK-STAT SIGNALING, INTERFERON SIGNALING, CYTOKINE SIGNALING, GROWTH HORMONE RECEPTOR SIGNALING, LEPTIN SIGNALING, INSULIN SIGNALING, PROLACTIN SIGNALING, SIGNALING BY INTERLEUKINS, SHP2 SIGNALING, ERBB2 IN SIGNAL TRANSDUCTION AND ONCOLOGY, EPO SIGNALING, CD40/CD40L SIGNALING, EGFR SIGNALING, KIT SIGNALING
**395**	**15.32**	**29**	G ALPHA SIGNALING, GPCR SIGNALING, METABOLISM OF NUCLEOTIDES, CAM PATHWAY, SIGNALING BY ERBB2, SIGNALING BY EGFR IN CANCER, GROWTH FACTOR SIGNALING
**182**	**14.59**	**12**	FOXM1 TRANSCRIPTION, PROGESTERONE-MEDIATED OOCYTE MATURATION,
**96**	**13.74**	**13**	REELIN SIGNALING, GLYCOGEN METABOLISM, SIGNALING BY INTERLEUKINS, WNT SIGNALING, PHOSPHOINOSITIDE TARGETS, IFN-GAMMA PATHWAY, REGULATION OF MICROTUBULE CYTOSKELETON, TGF-BETA SIGNALING, KIT SIGNALING, SEMAPHORIN INTERACTIONS
**321**	**10.99**	**5**	VITAMIN A AND CAROTENOID METABOLISM, CYTOCHROME P450
**145**	**10.97**	**11**	CELL CYCLE, DNA DAMAGE RESPONSE, P53 SIGNALING, P38 MAPK SIGNALING, SONIC HEDGEHOG RECEPTOR, EFP CONTROLS CELL CYCLE AND BREAST TUMORS GROWTH, TGF BETA SIGNALING, INTEGRATED BREAST CANCER PATHWAY, MAPK SIGNALING, FOXM1 TRANSCRIPTION, AMPK SIGNALING
**165**	**10.90**	**55**	NUCLEAR ESTROGEN RECEPTOR NETWORK, ATF-2 TRANSCRIPTION, RETINOIC ACID RECEPTORS-MEDIATED SIGNALING, SIGNALING MEDIATED BY P38-ALPHA AND P38-BETA, FOXA1 TRANSCRIPTION
**122**	**9.28**	**16**	BCR SIGNALING, TCR SIGNALING, NATURAL KILLER CELL CYTOTOXICITY, FC EPSILON SIGNALING, PI3K SIGNALING, JNK SIGNALING, NF-KAPPA B SIGNALING, INTERLEUKIN SIGNALING, EPO SIGNALING, CDC42 REGULATION, EGF-EGFR SIGNALING, RAC1 REGULATION, REGULATION OF RHOA
**143**	**8.97**	**11**	SKP2 DEGRADATION OF P27/P21, FOXM1 TRANSCRIPTION, P73 TRANSCRIPTION, PRL SIGNALING, ATR SIGNALING, P53 PATHWAY, RB TUMOR SUPPRESSOR/CHECKPOINT, EFP CONTROLS CELL CYCLE/ BREAST TUMOR GROWTH, AKT SIGNALING, AHR PATHWAY, NOTCH SIGNALING, ERBB SIGNALING, PI3K CASCADE, AMPK SIGNALING, C-MYC TRANSCRIPTIONAL REPRESSION, SMAD2/3 SIGNALING
**205**	**8.71**	**15**	DNA DAMAGE RESPONSE, CELL CYCLE, INTEGRATED BREAST CANCER PATHWAY, WNT SIGNALING, AURORA A SIGNALING, LKB1 SIGNALING, C-MYC TRANSCRIPTION REGULATION, BARD1 SIGNALING, ATM PATHWAY, PLK3 SIGNALING, HEDGEHOG SIGNALING, ERBB SIGNALING, P53 PATHWAY, HTERT TRANSCRIPTIONAL REGULATION, VEGFR1/ VEGFR2 SIGNALING, AP-1 TRANSCRIPTION, E2F TRANSCRIPTION, BRCA1 BRCA2 AND ATR IN CANCER, ARF INHIBITS BIOGENESIS, NUCLEAR ESTROGEN RECEPTOR ALPHA NETWORK, AMPK SIGNALING
**89**	**8.54**	**7**	REGULATION OF IGF ACTIVITY BY INSULIN-LIKE GROWTH FACTOR BINDING PROTEINS
**189**	**8.25**	**7**	C-MYB TRANSCRIPTION, TRANSCRIPTIONAL MISREGULATION IN CANCER, AP-1 TRANSCRIPTION
**348**	**8.20**	**29**	REGULATION OF ACTIN CYTOSKELETON, SHC CASCADE, FGFR SIGNALING, MAPK SIGNALING, PHOSPHOLIPASE C CASCADE, PI3K CASCADE, IRS SIGNALING, INSULIN SIGNALING, SYNDECAN SIGNALING, ERBB SIGNALING, FOCAL ADHESION, ANGIOGENESIS
**173**	**8.18**	**6**	METABOLISM OF NUCLEOTIDES, DRUG METABOLISM, E2F TRANSCRIPTION
**99**	**7.47**	**7**	P38 SIGNALING MEDIATED BY MAPKAP KINASES, CELL CYCLE, INSULIN-MEDIATED GLUCOSE TRANSPORT, PI3K SIGNALING MEDIATED BY AKT, INTEGRIN SIGNALING, MTOR SIGNALING, BETA CATENIN SIGNALING, ERBB1 SIGNALING, PDGFR-BETA SIGNALING, SIGNALING BY HIPPO
**12**	**7.25**	**23**	MAPK SIGNALING, ATF-2 TRANSCRIPTION, REGULATION OF P38-ALPHA AND P38-BETA, TOLL LIKE RECEPTOR CASCADE, ERBB1 SIGNALING, NGF SIGNALING, RAS SIGNALING
**Hepatocellular carcinoma**			
**408**	**72.64**	**24**	DRUG METABOLISM - CYTOCHROME P450, METABOLISM OF AMINO ACIDS, FATTY ACID METABOLISM GLYCOLYSIS/GLUCONEOGENESIS, ETHANOL OXIDATION, ARACHIDONIC ACID METABOLISM, TAMOXIFEN METABOLISM, VITAMIN A/CAROTENOID METABOLISM, ESTROGEN METABOLISM, AHR PATHWAY
**10**	**34.22**	**49**	DRUG METABOLISM, STEROID HORMONE BIOSYNTHESIS, RETINOL METABOLISM, CYTOCHROME P450 METABOLISM, METABOLISM OF AMINO ACIDS, TAMOXIFEN METABOLISM, FATTY ACID OXIDATION, BENZO(A)PYRENE METABOLISM, AHR PATHWAY, AFLATOXIN B1 METABOLISM, IL-10 SIGNALING
**513**	**22.92**	**4**	ALTERNATIVE COMPLEMENT PATHWAY, COMPLEMENT AND COAGULATION CASCADES
**579**	**19.55**	**13**	METABOLISM OF STEROID HORMONES AND VITAMINS A AND D, METABOLISM OF LIPIDS AND LIPOPROTEINS, MINERALOCORTICOID BIOSYNTHESIS, GLUCOCORTICOID METABOLISM
**603**	**17.58**	**6**	METABOLISM OF AMINO ACIDS
**31**	**14.24**	**14**	PPAR SIGNALING, FATTY ACID, TRIACYLGLYCEROL, AND KETONE BODY METABOLISM, ADIPOCYTOKINE SIGNALING, METABOLISM OF LIPIDS AND LIPOPROTEINS, AMPK SIGNALING
**97**	**13.93**	**5**	ONE CARBON POOL BY FOLATE, METABOLISM OF AMINO ACIDS AND DERIVATIVES
**361**	**9.55**	**16**	DNA REPLICATION, CELL CYCLE, UNWINDING OF DNA, SYNTHESIS OF DNA
**314**	**9.47**	**10**	FATTY ACID METABOLISM, GLYCEROLIPID METABOLISM, METABOLISM OF AMINO ACIDS
**34**	**9.08**	**14**	TOLL-LIKE RECEPTOR SIGNALING, HTLV-I INFECTION, ACTIVATION OF AP-1 TRANSCRIPTION FACTORS, MAPK SIGNALING, TWEAK SIGNALING, TGF BETA SIGNALING, INTERLEUKIN SIGNALING, RIG-I-LIKE RECEPTOR SIGNALING, HEPATITIS B VIRUS, IGF-1 SIGNALING, HEPATOCYTE GROWTH FACTOR RECEPTOR SIGNALING, JAK-STAT SIGNALING, FAS PATHWAY
**598**	**8.94**	**4**	KEAP1-NRF2 PATHWAY, METABOLISM OF AMINO ACIDS AND DERIVATIVES
**360**	**8.73**	**7**	INSULIN SIGNALING, GLYCOGEN METABOLISM, GLUCOSE METABOLISM, METABOLISM OF CARBOHYDRATES
**112**	**8.65**	**5**	MRNA SPLICING, MRNA PROCESSING
**515**	**8.46**	**10**	ONE CARBON POOL BY FOLATE, FOLATE METABOLISM
**257**	**8.23**	**5**	UREA CYCLE AND METABOLISM OF AMINO GROUPS, METABOLISM OF AMINO ACIDS
**153**	**7.29**	**5**	GLUCOCORTICOID & MINERALCORTICOID METABOLISM, METABOLISM OF STEROID HORMONES & VITA/D, METABOLISM OF LIPIDS & LIPOPROTEINS, PROSTAGLANDIN SYNTHESIS/ REGULATION
**123**	**7.22**	**7**	ONE CARBON FOLATE METABOLISM, METHYLATION, METABOLISM OF AMINO ACIDS
**254**	**7.03**	**6**	METABOLISM OF NUCLEOTIDES, METABOLISM OF AMINO ACIDS AND DERIVATIVES
**429**	**7.02**	**9**	SIGNAL TRANSDUCTION BY L1, MTOR SIGNALING, RSK ACTIVATION, PROSTATE CANCER, L1CAM INTERACTIONS, CREB PHOSPHORYLATION THROUGH THE ACTIVATION OF RAS, MAPK SIGNALING
**414**	**6.50**	**35**	MAPK SIGNALING, ATF-2 TRANSCRIPTION, CELL SIGNALING IN H. PYLORI INFECTION, ACTIVATION OF AP-1 TRANSCRIPTION FACTORS, FC EPSILON RI SIGNALING, NOD1/2 SIGNALING, GNRH SIGNALING, JNK SIGNALING, CD40/CD40L SIGNALING, C RIG-I-LIKE RECEPTOR SIGNALING, TGF BETA SIGNALING, VEGF SIGNALING, EGF-EGFR SIGNALING, FOSB GENE EXPRESSION
**Colorectal adenoma**			
**257**	**33.48**	**50**	CHEMOKINE SIGNALING, GPCR SIGNALING, NF-KAPPA B SIGNALING, CXCR3 SIGNALING, TOLL-LIKE RECEPTOR SIGNALING, NOD-LIKE RECEPTOR SIGNALING, INTESTINAL IMMUNE NETWORK FOR IGA PRODUCTION, INTERLEUKIN SIGNALING, CELL SIGNALING IN H. PYLORI INFECTION
**182**	**21.57**	**25**	TIGHT JUNCTION INTERACTIONS, TRANSENDOTHELIAL MIGRATION, CELL-CELL COMMUNICATION, CAMS
**158**	**18.94**	**9**	P75(NTR) SIGNALING, DEGRADATION OF THE ECM, ECM ORGANIZATION, SYNDECAN SIGNALING
**770**	**12.58**	**8**	ETHANOL OXIDATION, METABOLISM BY CYTOCHROME P450, TYROSINE METABOLISM, FATTY ACID METABOLISM, GLYCOLYSIS/GLUCONEOGENESIS, VITAMIN A/CAROTENOID METABOLISM
**14**	**11.51**	**5**	C-MYC TRANSCRIPTIONAL REPRESSION, SMAD2/3 SIGNALING, CELL CYCLE, PATHWAYS IN CANCER
**452**	**8.75**	**10**	GLYCOSPHINGOLIPID BIOSYNTHESIS, GLYCOSAMINOGLYCAN BIOSYNTHESIS
**487**	**7.16**	**28**	MAPK SIGNALING, ATF-2 TRANSCRIPTION, ACTIVATION OF AP-1 TRANSCRIPTION FACTORS, NOD-LIKE RECEPTOR SIGNALING, FC EPSILON SIGNALING, GNRH SIGNALING, TOLL-LIKE RECEPTOR SIGNALING, INTERLEUKIN SIGNALING, TGF BETA SIGNALING, VEGF SIGNALING, EGF-EGFR SIGNALING, KIT SIGNALING, RANKL-RANK SIGNALING, COLORECTAL CANCER, S1P2 PATHWAY, NONCANONICAL WNT SIGNALING, ARF6 PATHWAY, ERBB SIGNALING, TBXA2R SIGNALING
**301**	**7.06**	**7**	TRANSCRIPTIONAL MISREGULATION IN CANCER, RB REGULATION, INTERLEUKIN SIGNALING, C-MYB TRANSCRIPTION, INTERFERON SIGNALING, FOXA2/ FOXA3 TRANSCRIPTIONS, SMAD2/3 SIGNALING
**758**	**6.91**	**5**	METABOLISM OF AMINO ACIDS AND DERIVATIVES
**762**	**6.59**	**12**	WNT SIGNALING, SECRETIN FAMILY OF RECEPTORS, HTLV-I INFECTION, SIGNALING BY GPCR
**240**	**6.59**	**28**	G PROTEIN SIGNALING, CAM PATHWAY, PLC-GAMMA1 SIGNALING, NUCLEOTIDE METABOLISM, SIGNALING BY ERBB2, SIGNALING BY EGFR, SIGNALING BY FGFR, SIGNALING BY PDGF
**757**	**6.53**	**12**	METABOLISM OF STEROID HORMONES AND VITA/D, METABOLISM OF LIPIDS AND LIPOPROTEINS, GLUCOCORTICOID & MINERALCORTICOID METABOLISM, BILE ACID AND BILE SALT METABOLISM
**410**	**6.49**	**6**	JAK-STAT SIGNALING, CYTOKINE-CYTOKINE RECEPTOR INTERACTION, SHP2 SIGNALING, INTERLEUKIN SIGNALING, ROLE OF ERBB2 IN SIGNAL TRANSDUCTION AND ONCOLOGY
**412**	**6.21**	**9**	DNA REPLICATION, CELL CYCLE, UNWINDING OF DNA, ATR SIGNALING, E2F TRANSCRIPTION
**345**	**6.13**	**14**	NEUROTROPHIN SIGNALING, GNRH SIGNALING, CREB PHOSPHORYLATION, PKA ACTIVATION, CAM PATHWAY, INSULIN SIGNALING, PGC-1A REGULATION, RAS REGULATION, SMAD2/3 SIGNALING
**267**	**6.06**	**6**	METABOLISM OF PROTEINS
**334**	**6.04**	**12**	BETA-CATENIN PHOSPHORYLATION CASCADE, SIGNALING BY WNT, GLYCOGEN METABOLISM, PLATELET HOMEOSTASIS, DNA REPLICATION, CELL CYCLE, DNA DAMAGE RESPONSE
**111**	**5.74**	**11**	ECM-RECEPTOR INTERACTION, FOCAL ADHESION, INTEGRIN INTERACTIONS, NCAM INTERACTIONS, SYNDECAN SIGNALING, PROTHROMBIN ACTIVATION PATHWAY, PDGF SIGNALING, VEGFR3 SIGNALING
**54**	**5.73**	**4**	NONE
**125**	**5.67**	**20**	CHEMOKINE SIGNALING, G ALPHA SIGNALING, SIGNALING BY GPCR, ACTIVATION OF PKA, INTESTINAL IMMUNE NETWORK FOR IGA, CELL SIGNALING IN HELICOBACTER PYLORI INFECTION
**183**	**5.41**	**6**	BETA-CATENIN PHOSPHORYLATION CASCADE, CTLA4 INHIBITORY SIGNALING, GLYCOGEN METABOLISM, WNT SIGNALING, DNA REPLICATION, CELL CYCLE, IMMUNE SYSTEM, DNA DAMAGE RESPONSE
**156**	**5.38**	**4**	HEMATOPOIETIC CELL LINEAGE
**144**	**5.35**	**16**	CELL CYCLE, P38/MAPKAP SIGNALING, LKB1 SIGNALING, INSULIN-MEDIATED GLUCOSE TRANSPORT, PI3K/AKT SIGNALING, INTEGRIN SIGNALING, FOXO FAMILY SIGNALING, MTOR SIGNALING, ERBB1 SIGNALING, PDGFR-BETA SIGNALING, ATR SIGNALING, PLK1 SIGNALING, RB TUMOR SUPPRESSOR/CHECKPOINT, RAP1 SIGNALING, INTEGRATED CANCER PATHWAY, ATM PATHWAY, SHC SIGNALING, ARMS-MEDIATED ACTIVATION, IGF1 PATHWAY, IRS SIGNALING

### Breast cancer

BC fold-change measurements were filtered below an FDR-adjusted p-value of .01 and data associated with the remaining 2074 probes were used to weigh the network. The merge process reached a maximum size at step 2069, and the community search resulted in 8116 singletons, 206 pairs, 77 triplets and 174 modules (module size (3 > *size* ≤ 200).

**Figure 2:**
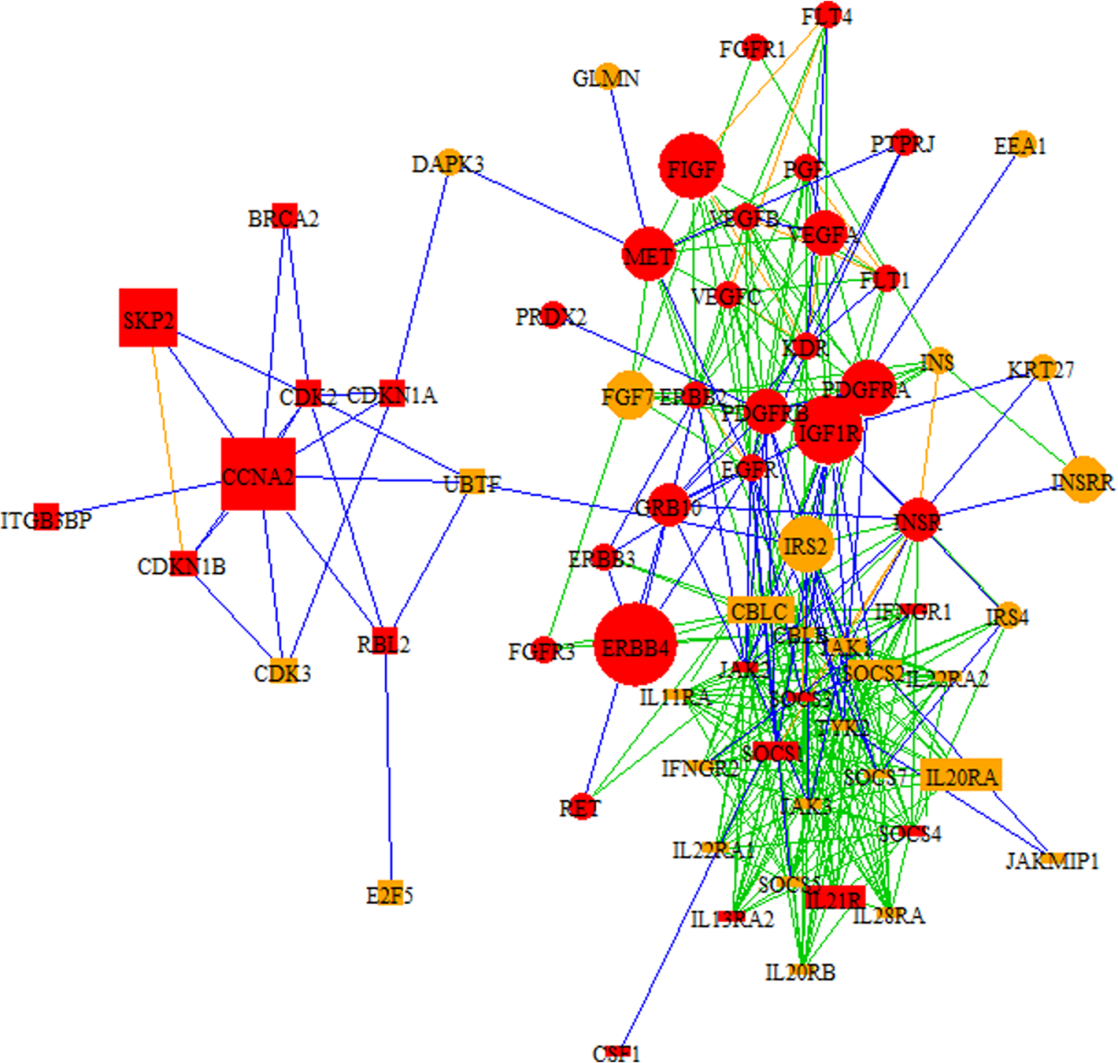
**Intersection of BC modules 143, 79 and 82.** Module 143, designated by square nodes, shows interactions among cyclins, *SKP2* and *BRCA2*. Module 79, designated by rectangular nodes shows interactions among cytokines, *SOCS* genes and genes in the *JAK-STAT* pathway. The *JAK-STAT* pathway is associated with B-cell growth and proliferation and a number of genes in this pathway are related to cancer. Module 82, designated by circular nodes, shows interactions among the *MET* oncogene and critical cancer-associated growth factors including *IGF1R, PDGFRA, VEGFA,* and *ERBB4*. Among genes in this module, *IRS2* and *FGF7* are differentially regulated and may be interesting targets for further research. Red nodes designate cancer-associated genes based on descriptions in OMIM. Node sizes correspond to the absolute values of the fold change of differentially regulated genes (up- or down-regulated). Blue edges are derived from HPRD, green from KEGG, and orange from both databases.

We examined the top-scoring modules in more detail by reviewing functional annotation and referring to visualizations of the modules (Table 2, Additional file 3). These modules were investigated to identify candidate genes, interactions with known cancer genes, and interactions between pathways. Among these top modules, we found highly differentially expressed candidate genes and relevant cancer interactions in modules 143, 79 and 82 (Figure 2). Module 143 is composed of cyclins regulating the cell cycle and a link to telomere formation (*E2F5*). *SKP2* is a known oncogene and interacts with cyclins to promote cell proliferation and evade apoptosis [51]. *SKP2* and cyclin *CCNA2* show significantly altered activity and interact with G2-phase cell cycle checkpoint genes *BRCA2* via *CDK2*. Module 79 involves interactions between cytokines and *JAK/STAT* regulation of signal transduction, cellular proliferation and differentiation. *SOCS1*, *SOCS2*, *SOCS3* and *CBLC* are involved in mediating *JAK/STAT* signaling, in the inflammatory response and in cellular growth. Differentially expressed genes include *SOC2*, *SOC3*, *CBLC,* and the interleukin receptor *IL20RA*; and the altered expression and coordinated interaction and of these genes suggest a concerted role in BC progression. Module 82 shows interaction between a number of growth factors and receptors, including *FIGF*, *IGFIR*, *PDGFRA*, *EGFR*, the *MET* oncogene, and tumor regulator *ERBB4*. The oncogene *MET* interacts with several growth factors, including *FGF7,* which mediates epithelial proliferation and has a potential role in gastric cancer [52]. *VEGFA* is a known metastatic vascular growth marker and a therapeutic target for breast cancer survival. *IRS2* affects proliferation and regeneration of cells, expression of this gene is critical during development and growth, and the gene may influence cancer survival [53,54]. *IRS2* and *FGF7* represent interesting candidate disease genes given their key functions and clinical relevance in aberrant cellular growth and proliferation.

### Hepatocellular carcinoma

HCC data included 16,360 probes after filtering by p-value. The maximal score was reached at 2393 steps, resulting in 7666 singletons, 352 pairs, 128 triplets, and 198 modules. At this step size, the maximum module size was 54 (module size (3 > *size* ≤ 54).

**Figure 3:**
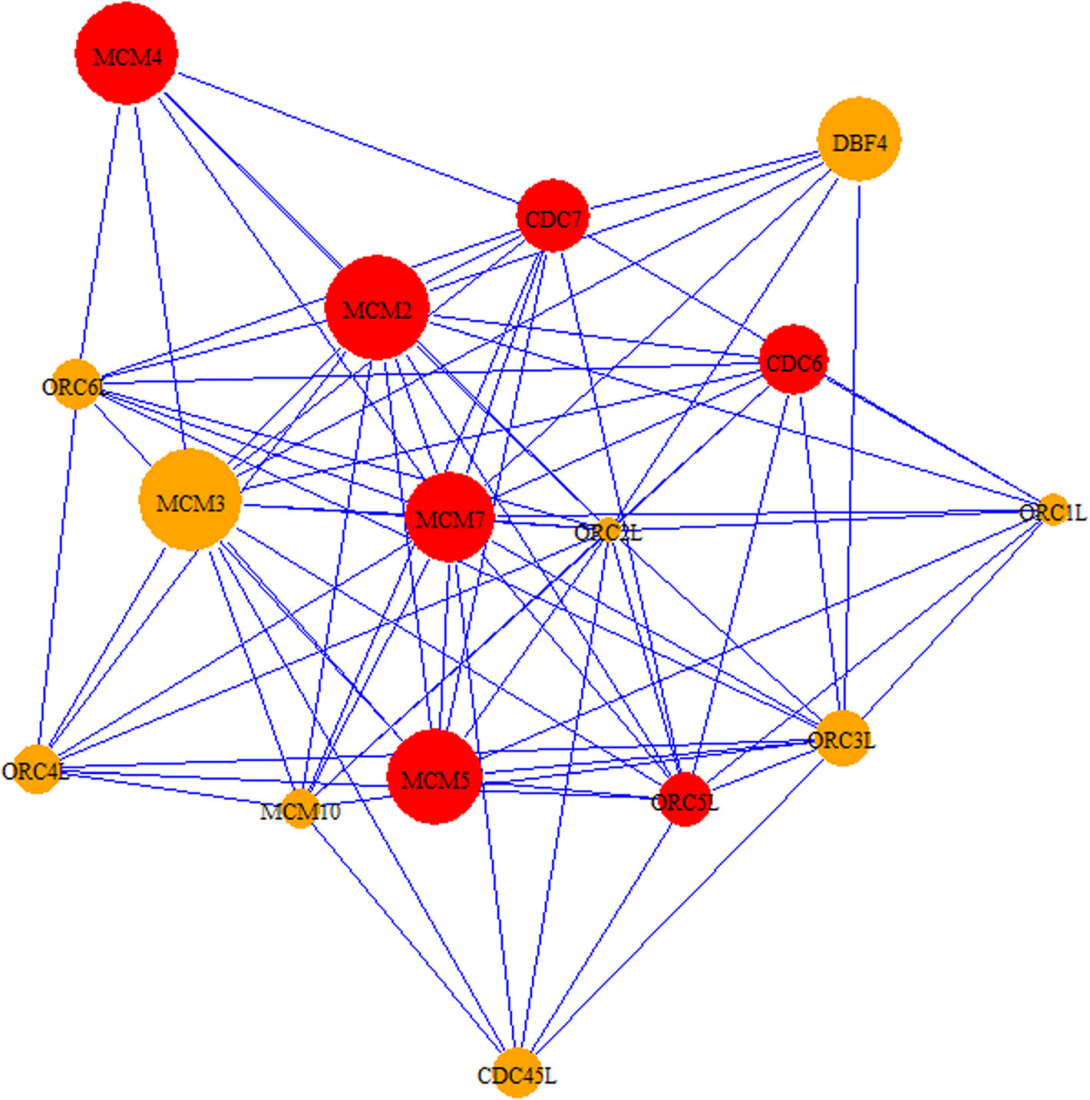
**HCC module 361.** Module 361 shows interactions among *MCM*, *ORC* genes involved in cell-cycle control, and *DBF4*. A number of *MC*M genes are known to be involved in cancer, and *DBF4* appears to play an interesting role in the cell cycle via interactions presented in this network and with other critical cell-cylce control genes. Red nodes designate cancer-associated genes based on descriptions in OMIM. Node sizes correspond to the absolute values of the fold change of differentially regulated genes (up- or down-regulated). Blue edges are derived from HPRD.

**Figure 4:**
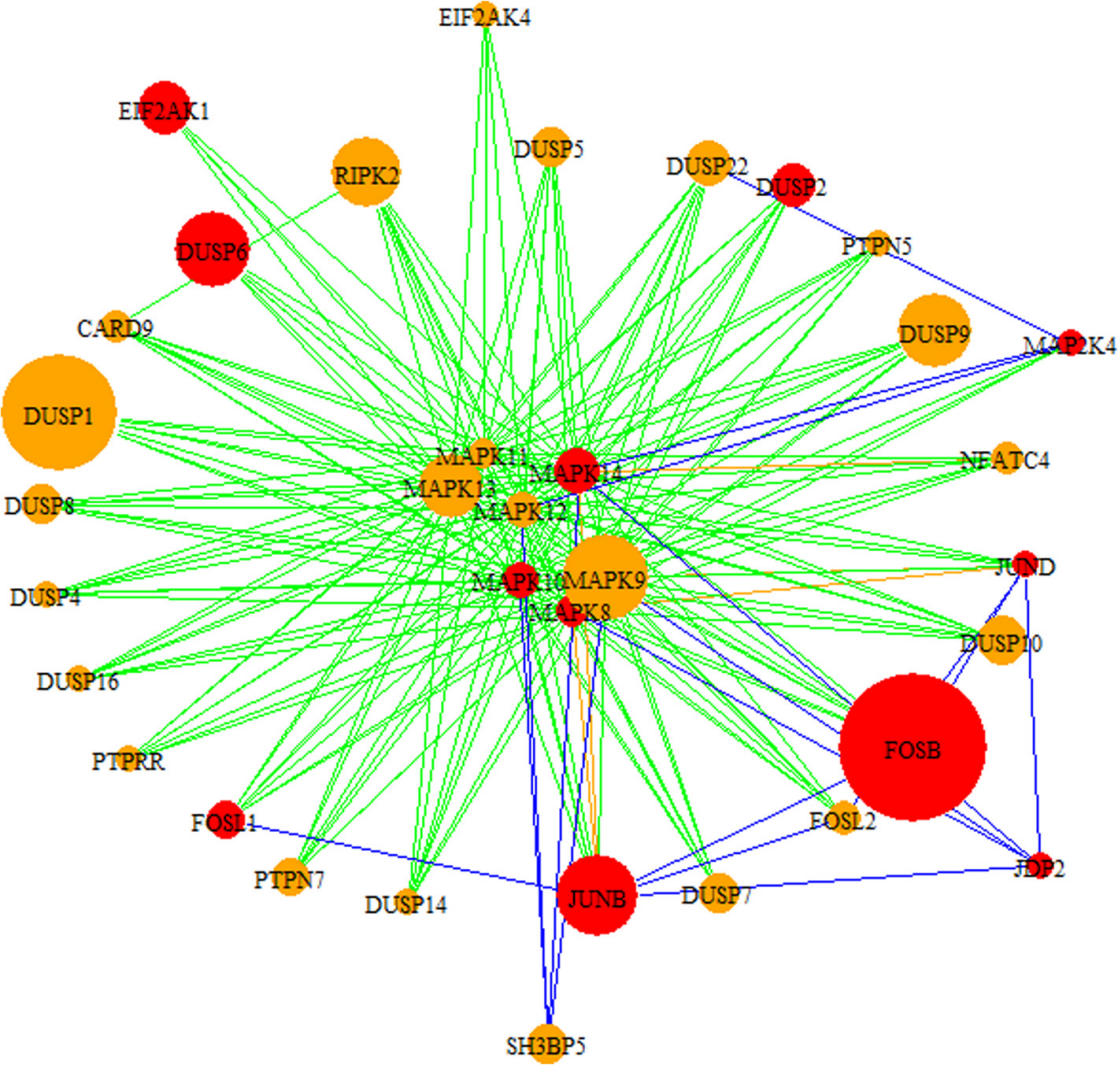
**HCC module 414.** Module 414 shows interactions among *MAPK*, *DUSP* genes and *FOSB* and *JUNB* oncogenes. The DUSP family of genes is known to regulate the activity of *MAP* kinases, a number of which play a role in cancer. This module presents interactions among *MAPK* genes and the oncogene *JUNB*, protooncogene *FOSB*, and *RIPK2*. *RIPK2* is not well-described, but appears to play a role in apoptosis. Red nodes designate cancer-associated genes based on descriptions in OMIM. Node sizes correspond to the absolute values of the fold change of differentially regulated genes (up- or down-regulated). Blue edges are derived from HPRD, green from KEGG, and orange from both databases.

The top-scoring modules are summarized in Table 2 and Additional file 4. We reviewed highly expressed candidate genes and interactions modules 361, 429 and 414 in greater detail. Module 361 (Figure 3) consists of interactions between a family of cyclins, origin recognition complexes, and minchromosome maintenance genes. Kinase activation of *CDC7* is dependent on expression of *DBF4*, and both genes are highly expressed in cancer [55]. *MCM5* forms a complex with *MCM2*[56], a candidate oncogene phosphorylated by *CDC7*. *ORC5L* associates with *CDC7* and *MCM5* in the network and this group of genes display altered expression in HCC tissue. These genes exhibit high differential expression and have implications in tumor formation due to their role in regulating the cell-cycle and cellular proliferation. Module 429 (Additional file 4), shows upregulation of *IGFI,* which is known to alter cancer risk [57], and interaction with the oncogene *NOV*, and transcription factors *RPS6KA2* and *RPS6KA6*. These transcription factors are associated with the *RSK* family of genes, involved in activating map kinase growth signaling, cell cycle control and differentiation. Given their importance in cellular development, their potential implication in cancer [58,59] and association with *IGFI* and *NOV*, these *RSK* transcription factors are compelling candidate cancer genes. Module 414 (Figure 4) shows the interaction between *MAPK* signaling genes, *DUSP* genes and *FOS* and *JUND* oncogenes. The *DUSP* genes regulate the activity of *MAPK* signaling cascades, and several map kinase targets are known to be involved in aberrant proliferation in cancer. The kinase *RIPK2* has an important function in apoptosis and interactions with the *MAPK* signaling and high differential expression in this module suggest a potential role in tumorigenesis. Due to their association with known cancer genes and high differential expression, *DUSP1*, *DUSP9*, *MAPK9* and *RIPK2* genes are promising targets for therapeutic research.

### Colorectal cancer

CCA data included 21,648 probes after filtering by p-value. The maximal score was reached at 2967 steps. The resulting community structure included 6879 singletons, 385 pairs, 149 triplets and 253 modules. The maximum module size at this step was 160 (module size (3 > *size* ≤ 160).

**Figure 5:**
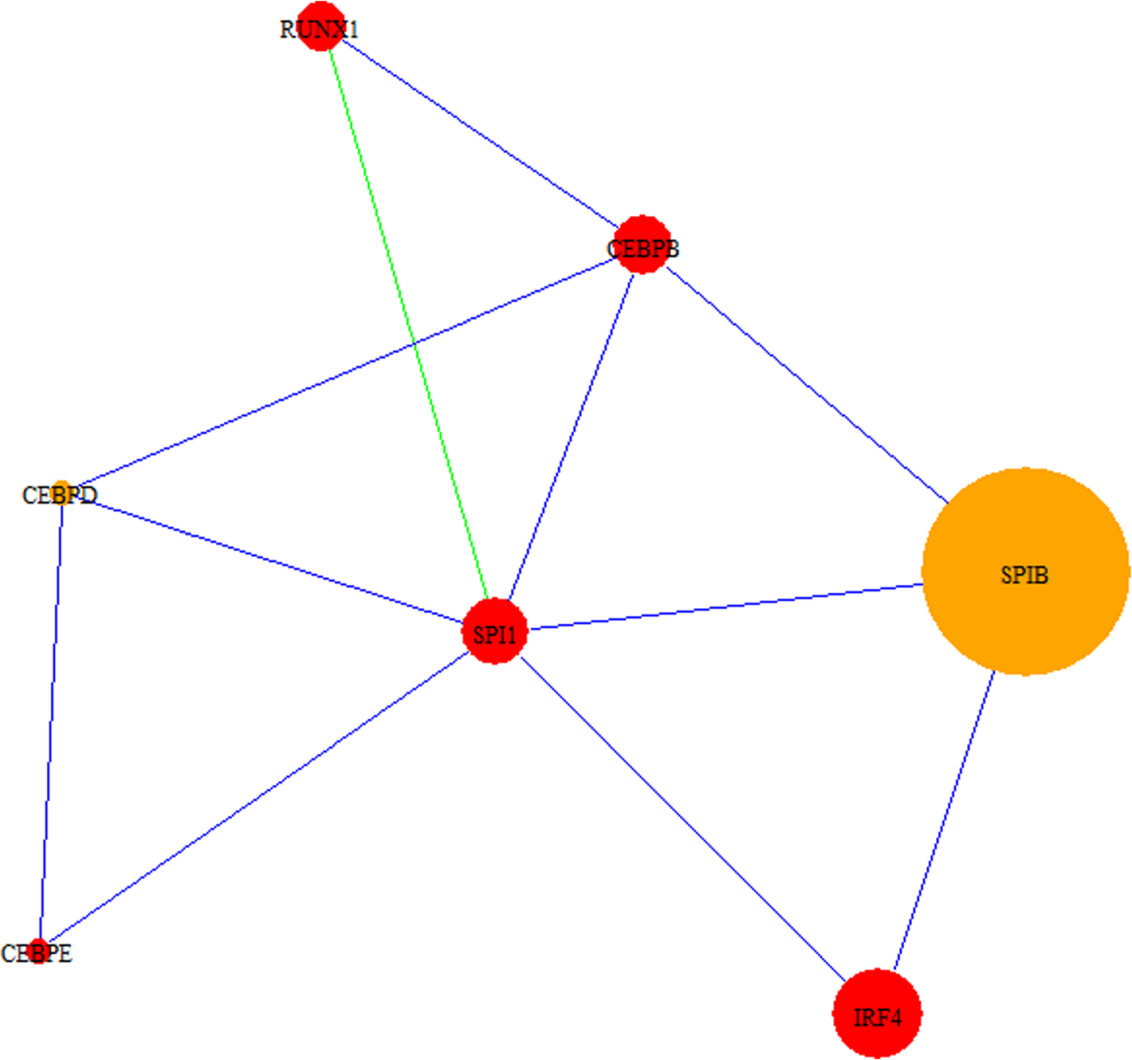
**CCA module 301.** Module 301 shows interactions among cancer-related transcription factors. The role of *SPIB* in cancer is of interest is as this transcription factor is highly differentially regulated in this module and interacts closely with known cancer genes. Node sizes correspond to the absolute values of the fold change of differentially regulated genes (up- or down-regulated). Blue edges are derived from HPRD, green from KEGG, and orange from both databases.

**Figure 6:**
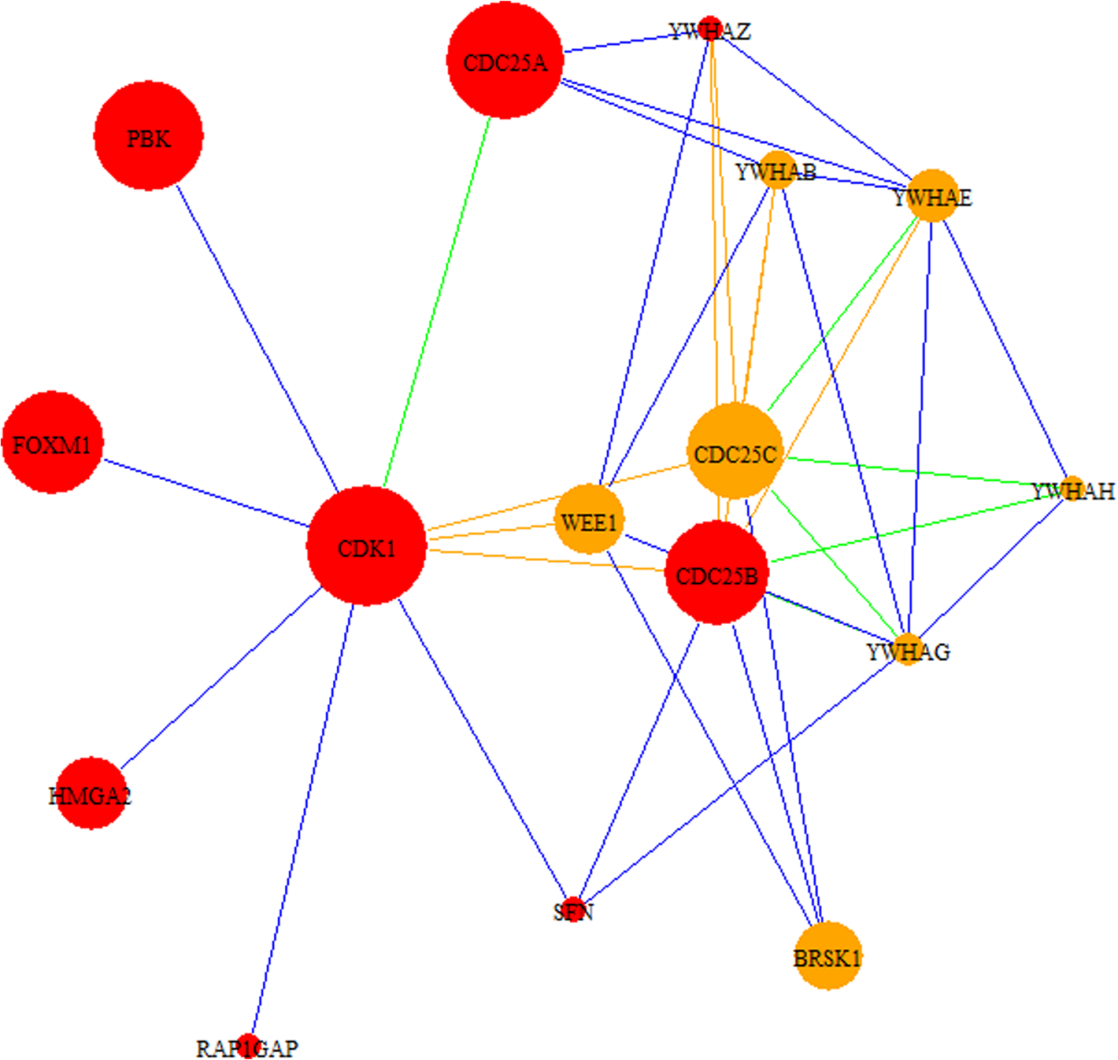
**CCA module 144.** Module 144 shows interactions among cell cycle regulatory genes and *FOXM1* oncogene. *WEE1*, *CDC25C*, *YWHAE* and *BRSK1* are also involved in cell cycle control and interact closely with cancer-associated genes, but are not themselves well-described as cancer genes. Also of note, *WEE1* and *CDC25C* are known to play an antagonistic role in regulating the cell cycle. Red nodes designate cancer-associated genes based on descriptions in OMIM. Node sizes correspond to the absolute values of the fold change of differentially regulated genes (up- or down-regulated). Blue edges are derived from HPRD, green from KEGG, and orange from both databases.

The top-scoring modules are summarized in Table 2 and Additional file 5. We reviewed modules 301, 144, and 762 in detail based on the differential expression of potential cancer-associated genes and relevance of their functional annotation in cancer. There are three known oncogenes in module 301 (Figure 5): *SPI1*, *RUNX1,* and *IRF4*. *CEBPB* and *CEBPE* interact with these oncogenes, affect cellular proliferation, and alter tumor development and cancer risk [60,61]. Transcription factors *SPI1*and *RUNX1* participate in hematopoietic stem cell formation and can lead to the development of multiple cell lineages in cancer [62,63]. These genes show altered expression in the network, and specifically, the role of the highly differentially regulated transcription factor *SPIB* in colorectal cancer is an interesting area for further research. Module 144 (Figure 6) shows interactions between *CDK1*, a key regulator of the cell cycle and proliferation, and genes associated cellular division and growth in cancer: *PBK*, *HMGA2,* and *FOXM1*. Putative candidates among neighboring genes include *BRSK1*, *WEE1,* and *CDC25C*, which are involved in cell-cycle checkpoints and are overexpressed in CCA. Specifically, *WEE1* and *CDC25C* are significantly differentially regulated and are known to play a mutually antagonistic role in cell-cycle control. *BRSK1* is not well described, but exhibits key interactions with genes involved in cell-cycle control. Module 762 (Additional file 5) consists of interactions among *SFRP1* and *SFRP2* genes and *FZD* genes in the *Wnt* pathway. The *Wnt* pathway is involved in cell polarity and malignant cell transformation in colorectal cancer, and the *SFRP1* and *SFRP2*[64] genes interfere with normal *Wnt* signaling. *SFRP* genes and most frizzled *FZD* genes in the module show altered expression. Given the topology of *SFRP1* and *SFRP2* as hubs in this module, these genes appear to play a central role the *Wnt* pathway and CCA development. Descriptions of genes highlighted in BC, HCC and CCA modules are summarized in Additional file 6.

### Overlap with GSEA

We analyzed BC, HCC and CCA data using GSEA and canonical pathways in MSigDB. The overlap of top-scoring *Walktrap-GM* annotation with GSEA results was evaluated by cross-validating the top 10 GSEA pathways with pathways significantly overrepresented in our dataset (p ≤ .01). Notably in the BC data, module 205 overlaps with the following highest-ranking GSEA pathways, Cell Cycle (p = 1.56 × 10^-08^), Ubiquitin-mediated Proteolysis (p = 7.66 × 10^-05^), DNA Replication (p = .0085), G1-S Phase (p = 4.23 × 10^-08^), the ATR-BRCA pathway (p = .0005), and Apoptosis (p = .0074). Module 224 exhibits significant overrepresentation in Pyrimidine Metabolism (p = 1.37 × 10^-24^) Apoptosis (p = 3.08 × 10^-06^) and DNA Replication (p = 7.43 × 10 ^-05^) pathways which are among the top 10 enriched pathways in GSEA.

HCC module 408 shows significant enrichment with the 10 highest-ranking GSEA results, including Tryptophan (p = 6.89 × 10^-08^), Tyrosine (p = 2.92 × 10^-29^), Beta-Alanine (p = 6.05 × 10^-07^), and Arachidonic Acid (p = 2.15 × 10^-14^) metabolism. Significant pathways overrepresented in Module 314 that overlap with the top GSEA modules are Tryptophan (p = 6.10 × 10^-13^), Propanoate (p = 6.34 × 10^-11^), Tyrosine (p = .0007), Beta-Alanine (p = 8.43 × 10^-20^), Valine, Leucine and Isoleucine (p = 3.40 × 10^-10^), Lysine (1.94 × 10^-12^), Phenylalanine (.0001), and Glycerolipid (9.86 × 10^-10^) metabolism.

In CCA, module 144 overlaps with the top 10 ranked pathways in GSEA, including the ATM (p = 1.78 × 10^-05^), Cell Cycle (p = 9.92 × 10^-19^), P53 (p = .0046), and ATR (p = 2.1 × 10^-07^) pathways, and module 412 shows overlap with the Cell Cycle (p = 8.58 × 10^-16^) and G1 to S Phase (p = 6.91 × 10^-21^). Overall, the consistency observed with GSEA suggests that processes similar to those highlighted by GSEA are also detected by highly-ranked *Walktrap-GM* network modules.

### Comparison with related graph-clustering platforms

Properties of *Walktrap-GM* are compared to those of several other approaches in Table 3, including heuristics for clustering, learning methods and parameter tuning. Compared with *MCL*[65], *Affinity-Propagation*[66]*,* and *Netwalker*[31] that use similarity values to cluster nodes, *Walktrap-GM* clustering can be implemented using similarity or significance values, here we use significance scores to perform a supervised search for modules that are associated with the phenotype of interest. The *Matisse* algorithm clusters by similarity, using the iterative addition of significant seed genes to find optimal scoring modules. *jActiveModules* uses differential expression to build subnetworks; however, the platform is restricted to using only p-values. Among these approaches, *Walktrap-GM* allows for semi-supervised learning of non-overlapping modules using significant differential expression as edge weights.

**Table 3:** Comparison of approaches to module-finding in biological networks

**Approach**	**Method**	**Heuristic for subgraph search**	**Cluster learning method**	**Cluster overlap**	**Tuning parameters**	**Weighted networks**	**Platform**
**Walktrap-BM**	Random walk	Differential expression or pairwise similarity	Semi-supervised	No	Modularity, size, score	Yes	R on Unix, Windows, Mac
**jActiveModules**[20,21]	Simulated annealing	Differential expression (P-values only)	Semi-supervised	Optional	K-modules, number of paths, iterations	Yes	Cytoscape plugin on Windows, Mac, Linux
**Matisse**[22]	Seed clustering	Pairwise similarity and significant seed nodes	Semi-supervised	Yes	Module size, seed number	Yes	Linux, Windows
**NetWalker**[23,31]	Random walk	Differential expression	Unsupervised	Optional	K-Modules	Yes	Windows, Mac
**Affinity Propagation**[66]	Seed-based message propagation	Pairwise similarity	Unsupervised	Yes	Preference values, seed number	Yes	Matlab, R on Windows and Linux
**MCL**[65]	Random walk	Pairwise similarity	Unsupervised	No	Granularity	Yes	UNIX platforms

**Figure 7:**
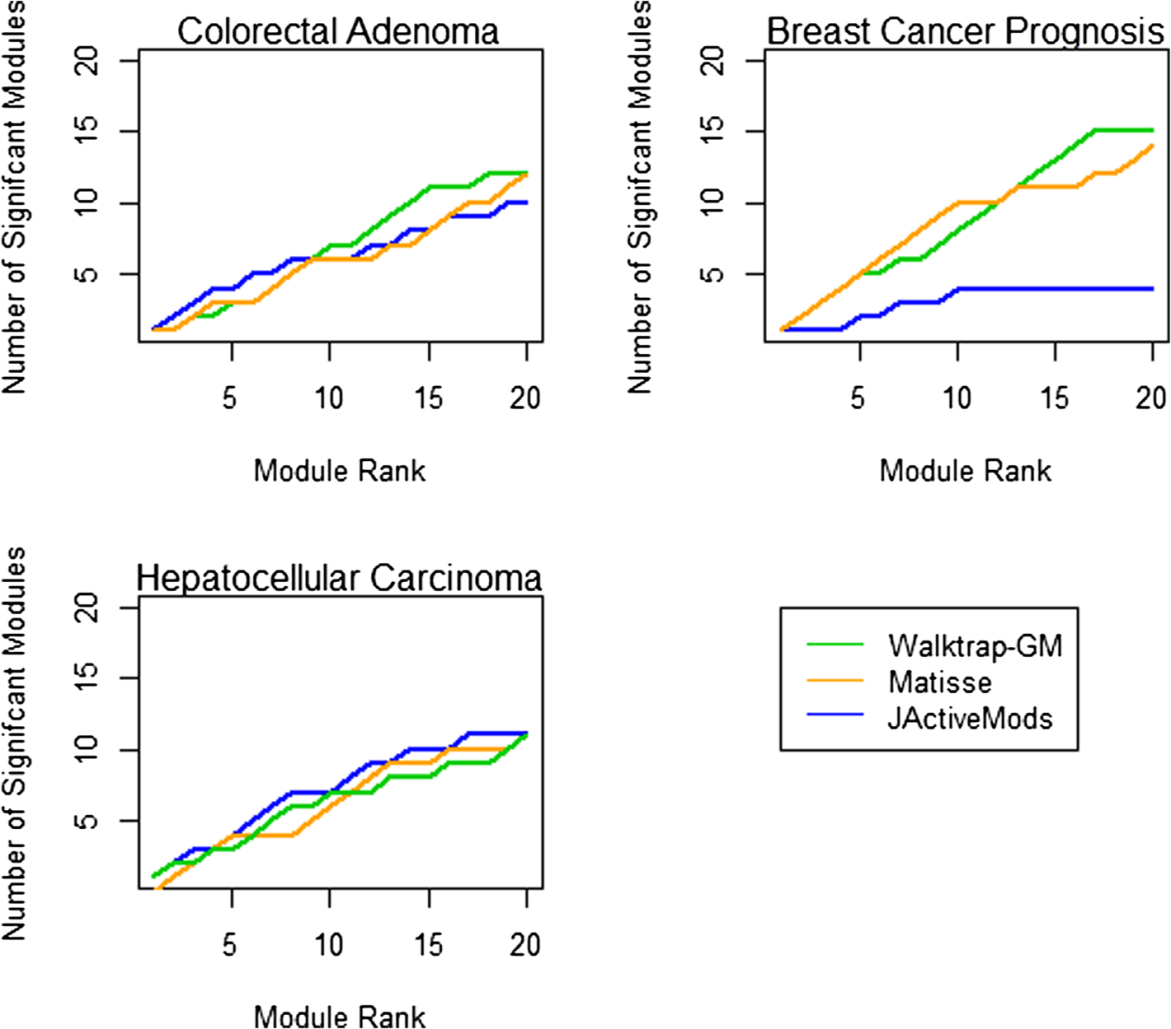
**Comparison of top modules from *Walktrap-GM*, *Matisse, jActiveModules*.** Performance in finding modules significantly enriched with known cancer genes,across breast cancer (BC) and hepatocellular carcinoma (HCC) and colorectal cancer data (CCA). Green lines show *Walktrap-GM* performance, blue *jActiveModules,* and orange *Matisse*. *Walktrap-GM* performs as well as or better than the other approaches across datasets. In the BC data, blue *jActiveModules* resulted in one very large and significant module of 981 nodes, but few significant modules overall. *Matisse* includes overlapping significant genes within its modules where *Walktrap-GM* does not and *jActiveModules* is configured not to inlcude overlap.

**Figure 8:**
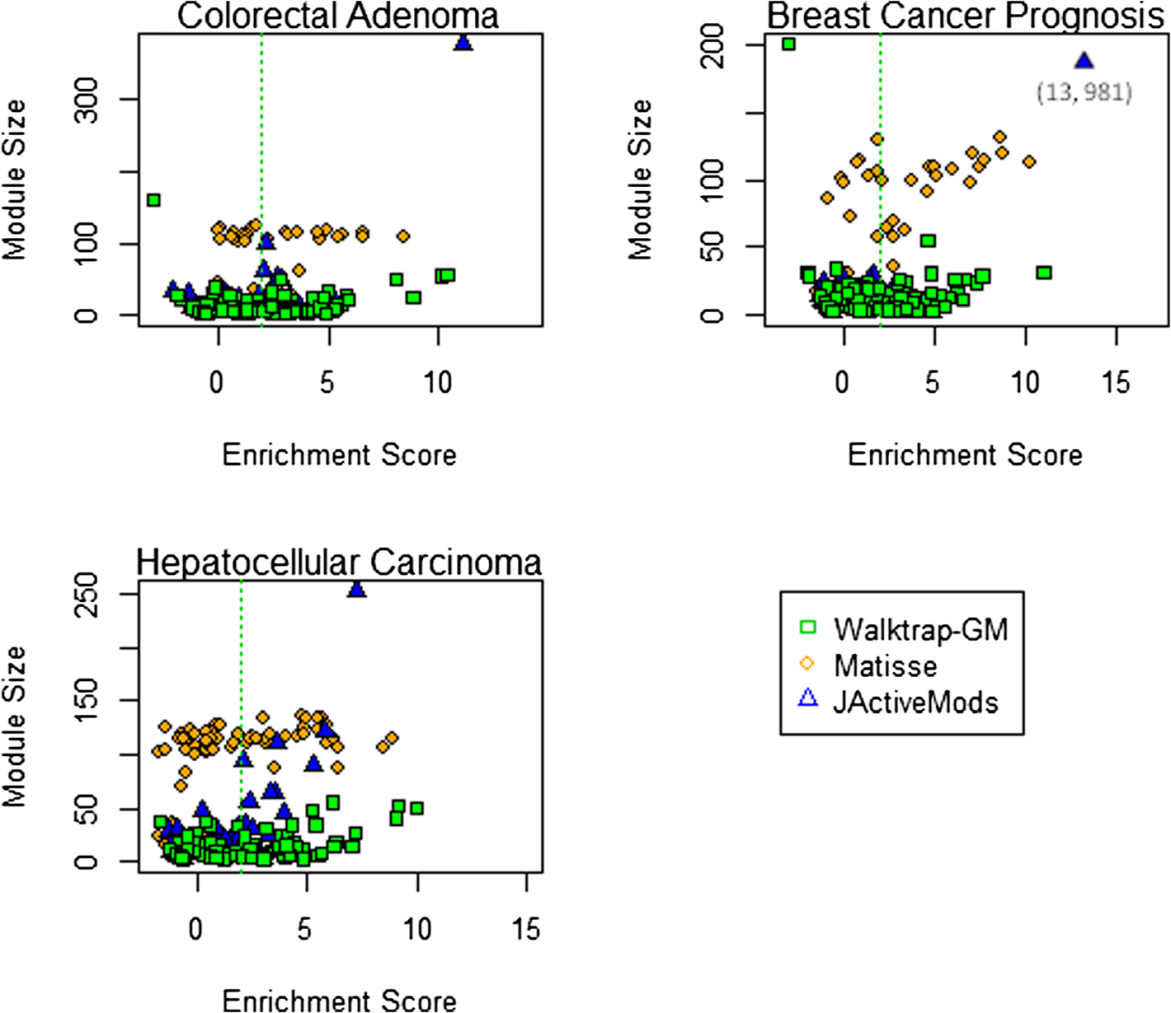
**Distribution of module sizes by score for each dataset.***Walktrap-GM* markers are noted in green, *Matisse* in orange, and *jActiveModules* in blue. *Walktrap-GM* includes a size threshold of 200, and identifies significant modules that are generally smaller. Smaller modules tend to have more specific and informative functional interpretation; the functional annotation of large modules may be too general to be meaningful.

We evaluate the performance of *Walktrap-GM* with two platforms widely used to find network modules using gene expression data in interaction networks, *jActiveModules*[20] and *Matisse*[22]. Results are summarized in Figure 7. *Walktrap-GM* generally performed as well or better than *Matisse* or *jActiveModules* using the HCC and CCA data and performs consistently well overall. *Matisse* modules include overlap, so the corresponding set of top modules display greater coverage of significant interactions, but redundant sets of significant genes. By excluding overlapping genes, *Walktrap-GM* focuses the search for unique interactions across modules. We also consider module size; distribution of module sizes for each dataset and platform are shown in Figure 8. *jActiveModules* generated several large modules, including a module of 981 nodes in BC and a module of 377 nodes in CCA. The majority of significant modules generated by *Matisse* include more than 100 nodes. Generally, large clusters demand further mining to discover the most relevant interactions and genes in each module. The smaller distribution of module sizes associated with *Walktrap-GM* highlights a more specific and informative set of biological interactions that facilitates interpretation of modules; where the functional annotation of larger modules that may otherwise be too general to be meaningful. Further, the time required to run the *Walktrap* algorithm (summarized in Additional file 1) compared favorably to the other tools, on a scale of minutes for each dataset on a 64-bit, 8 GB. 2.8 GHz, machine, compared to several hours running *jActiveModules* and *Matisse*.

## Conclusions

Network analysis provides a framework to search for communities of genes associated with disease by modeling their coordinated behavior and biological knowledge of their interactions. We use a random walk algorithm optimized to search for communities in large networks, to mine for disease genes in a weighted interaction network. The network is weighted with differential gene expression corresponding to adenoma development, tumor growth, and cancer progression. This approach is used to discover cancer-associated modules in a network of biological interactions weighted by differential gene expression of breast cancer, hepatocellular carcinoma and colorectal cancer data.

*Walktrap-GM* identifies modules relevant to the etiology of multiple cancer outcomes, and suggests interactions among promising candidate genes for further study of their role in cancer or potential therapeutic intervention. Functional analysis of modules discovered in this analysis reveals strong enrichment of cancer-related pathways and known cancer genes. Pathways enriched across the BC, HCC and CCA data include those involved in cell cycle control, DNA replication, DNA damage and repair, amino acid metabolism, inflammation, and cell adhesion and migration. Specifically, several genes may represent targets for further research, including *CBLC* or *IRS2,* which influence breast cancer survival; transcription factors *RPS6KA2* and *RPS6KA6*, the interaction among *MCM/CDC* and *ORC* cell cycle control genes, and *DUSP1*, *DUSP9*, *RIPK2* and *MAPK9* in the onset of hepatocellular carcinoma; or cell-cycle genes *BRSK1*, *WEE1*, *CDC25C,* and the transcription factor *SPIB* in colorectal adenoma development. Significant interactions among these candidate genes can be used to generate hypotheses and experimentally validate the functional significance and therapeutic value of these targets in cancer.

This network analysis approach has potential applications to a diverse body of biological data, for example, protein complex prediction, functional prediction, gene variation, and regulatory interactions. Similar functional annotation or correlation expression can be applied to the network to predict protein complexes or functional annotation of unknown genes. SNP or eQTL data can be integrated to search for modules that demonstrate significant genetic variation in case–control data. Transcription factor, methylation or miRNA data can be coupled with their regulatory targets to discover significant regulatory modules. Text-mining applications can highlight significant relationships among terms by mapping the co-occurrence of their expressions in the literature. Related edge weighting schemes may include correlation coefficients, significance values based on association with phenotypes of interest, or confidence scores that reflect the level of certainty corresponding to a biological interaction.

These findings show that *Walktrap-GM* identifies biologically relevant modules associated with cancer and performs well compared with other module search platforms, *Matisse* and *jActiveModules*. Strong performance combined with smaller, more specific, and non-overlapping modules, facilitates biological interpretation of these results. These modules reflect known pathways in cancer and present hypotheses for clinical studies. Future work may include an analysis across additional cancer and other complex disease data, or apply these methods to integrate more classes of genomic data such as SNP, miRNA or next generation sequencing data.

## Availability and requirements

∎Project name: Walktrap-GM

∎Project home page: http://github.com/petrochilos/walktrap-GM.git

∎Operating system(s): platform independent

∎Programming Language: R

∎Other Requirements: recommended minimum requirements include 8 MB RAM, 2.8 GHz processor, 64-bit system

∎License: GNU GPL

## Abbreviations

BC: Breast cancer; HCC: Hepatocellular carcinoma; CCA: Colorectal cancer adenoma.

## Competing interests

The authors have no competing interests to declare.

## Authors’ contributions

DP conceived of the study and carried out data, graph, and statistical analysis. NA, AS, and JG participated in the design of the study, including technical and statistical evaluation, and review of the manuscript. All authors read and approved the final manuscript.

## Supplementary Material

Additional file 1 Walktrap performance by Network Size and Density. Click here for file

Additional file 2 List of labeled cancer genes curated from Online Mendelian Inheritance in Man database (OMIM). Click here for file

Additional file 3 Visualization of top ranked BC modules. Click here for file

Additional file 4 Visualization of top ranked HCC modules. Click here for file

Additional file 5 Visualization of top ranked CCA modules. Click here for file

Additional file 6 Key genes described in BC, HCC and CCA modules. Click here for file
