# Supplementary material for: Using random walks to identify cancer-associated modules in expression data
Source: BioData Min. 2013 Oct 15;6:17. doi: 10.1186/1756-0381-6-17 (PMC4015830; doi:10.1186/1756-0381-6-17)
Supplement: Additional file 1 — Walktrap performance by Network Size and Density. [file 1756-0381-6-17-S1.docx]

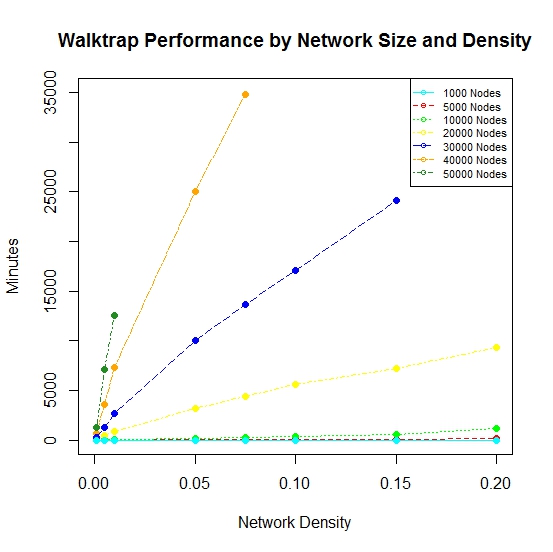


**Plot of Network Size and Density.** This graph summarizes runtime performance of Walktrap random walk community search algorithm, generating the dendrogram for community membership. Tests were run on a 64-bit, 16GB Amazon Cloud server with 2 EC2 Compute Units (4 virtual cores with 2 EC2 Compute Units each). Network sizes are shown by colored lines, and plotted values show run time in minutes for the range of densities (.001-.2). Points not plotted did not have sufficient memory (50000 nodes above .01 density, 40000 nodes above .075 density, and 30000 nodes above .15 density).
