# Supplementary figures and images for: Using random walks to identify cancer-associated modules in expression data
Source: BioData Min. 2013 Oct 15;6:17. doi: 10.1186/1756-0381-6-17 (PMC4015830; doi:10.1186/1756-0381-6-17)

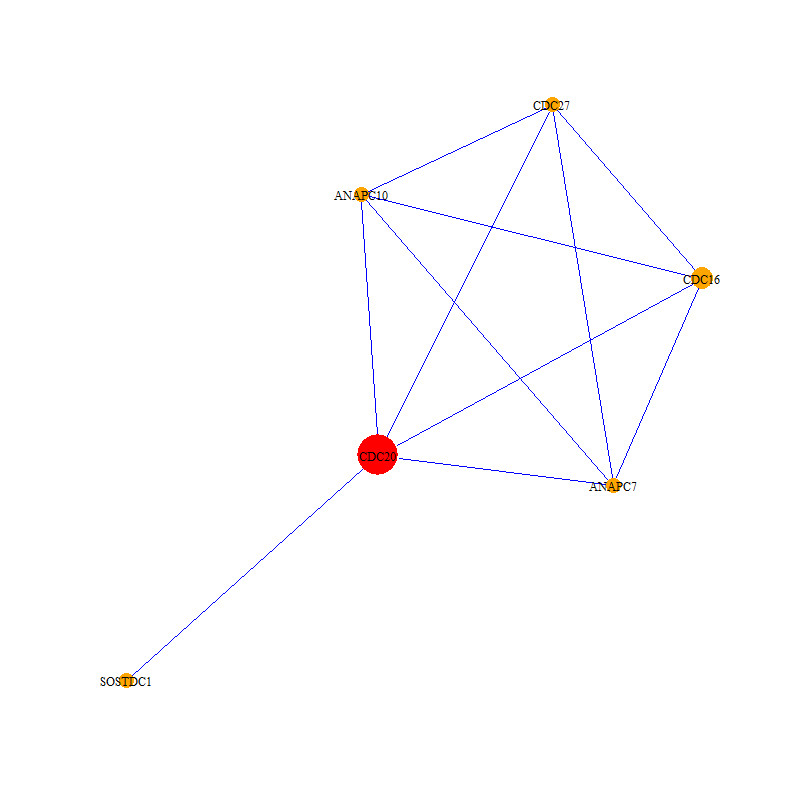

Supplement: Additional file 3 — Visualization of top ranked BC modules. [file 1756-0381-6-17-S3.zip › BCModules/plotBC.101.tif]

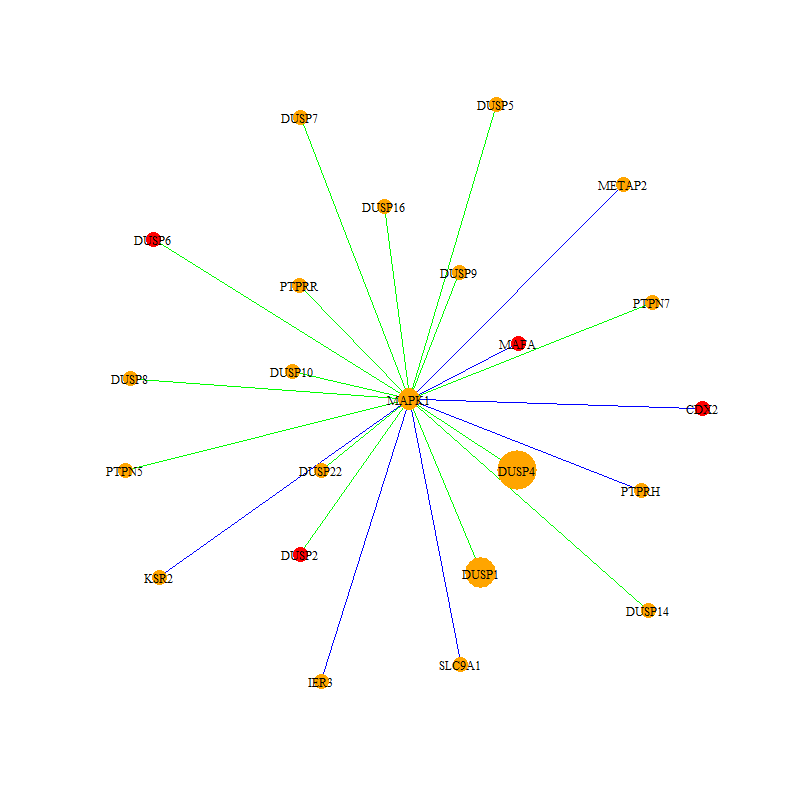

Supplement: Additional file 3 — Visualization of top ranked BC modules. [file 1756-0381-6-17-S3.zip › BCModules/plotBC.12.tif]

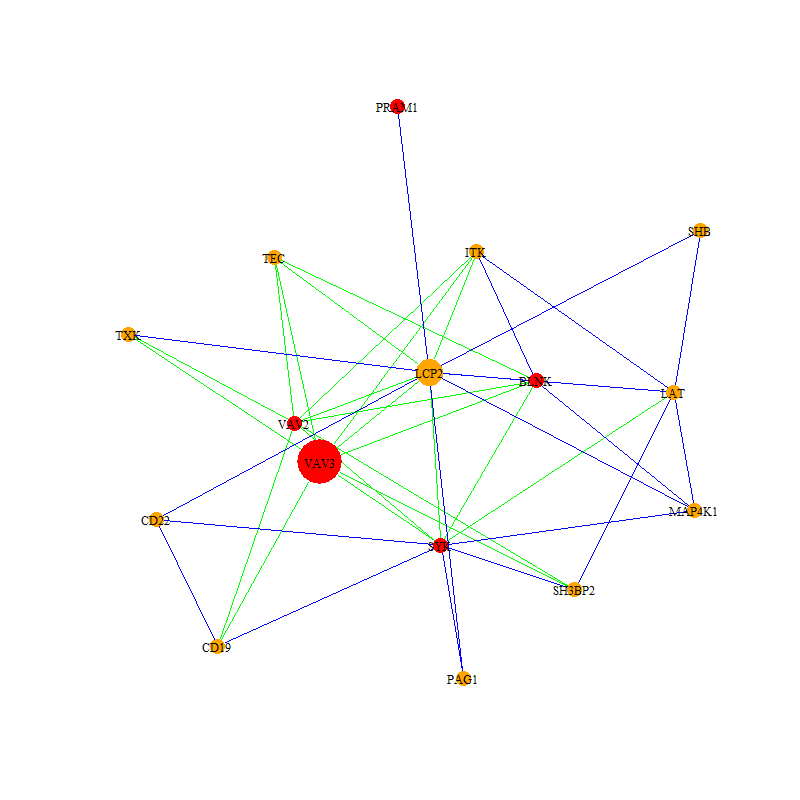

Supplement: Additional file 3 — Visualization of top ranked BC modules. [file 1756-0381-6-17-S3.zip › BCModules/plotBC.122.tif]

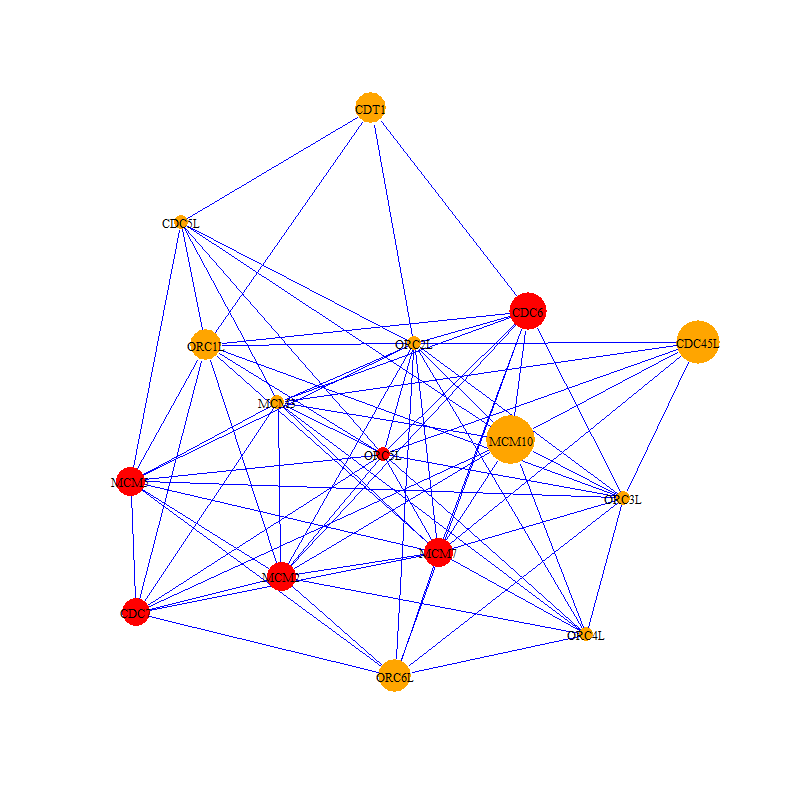

Supplement: Additional file 3 — Visualization of top ranked BC modules. [file 1756-0381-6-17-S3.zip › BCModules/plotBC.134.tif]

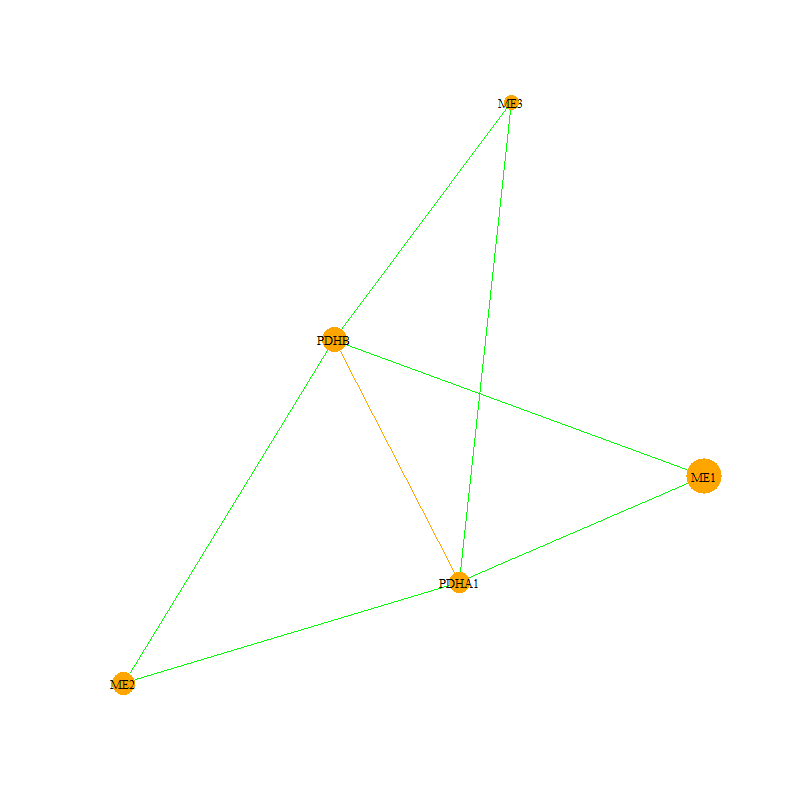

Supplement: Additional file 3 — Visualization of top ranked BC modules. [file 1756-0381-6-17-S3.zip › BCModules/plotBC.141.tif]

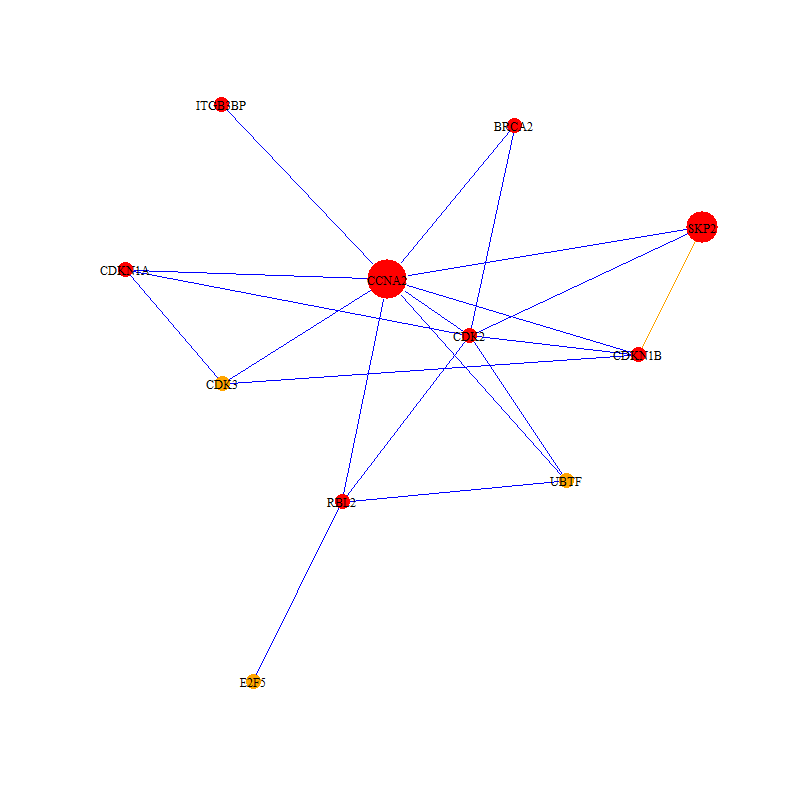

Supplement: Additional file 3 — Visualization of top ranked BC modules. [file 1756-0381-6-17-S3.zip › BCModules/plotBC.143.tif]

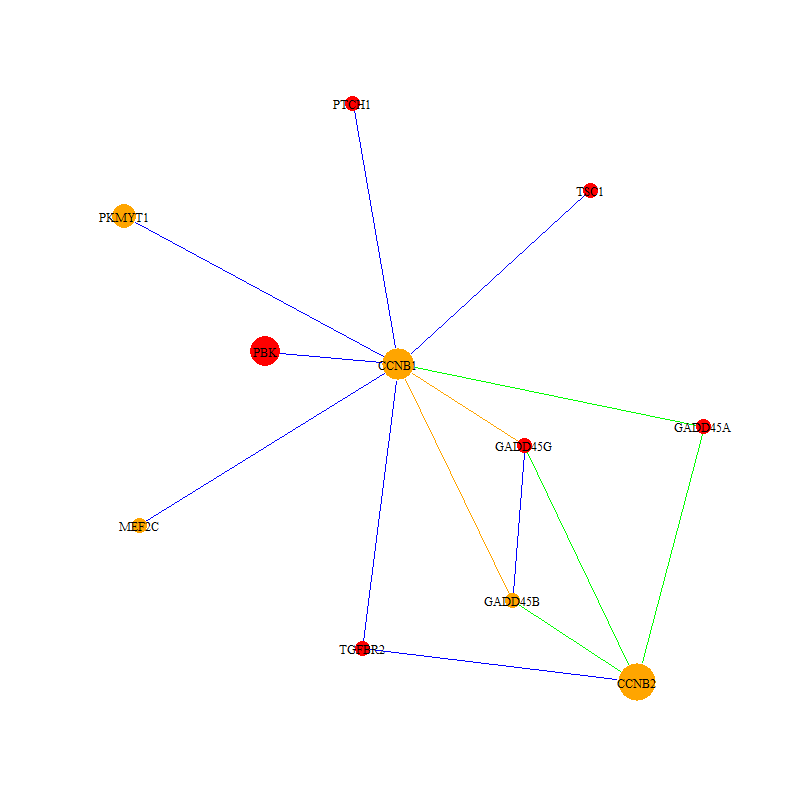

Supplement: Additional file 3 — Visualization of top ranked BC modules. [file 1756-0381-6-17-S3.zip › BCModules/plotBC.145.tif]

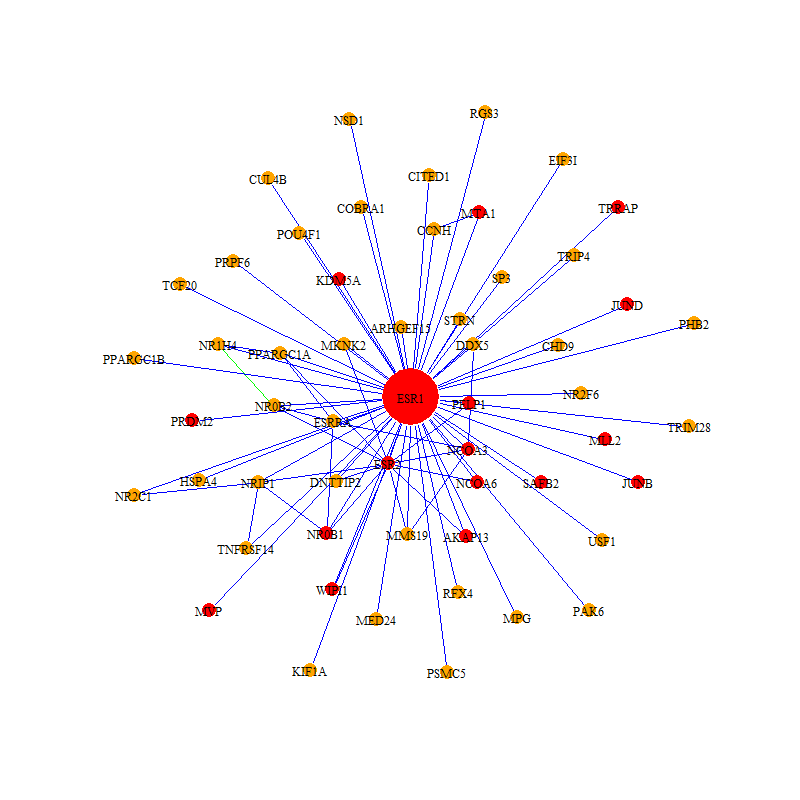

Supplement: Additional file 3 — Visualization of top ranked BC modules. [file 1756-0381-6-17-S3.zip › BCModules/plotBC.165.tif]

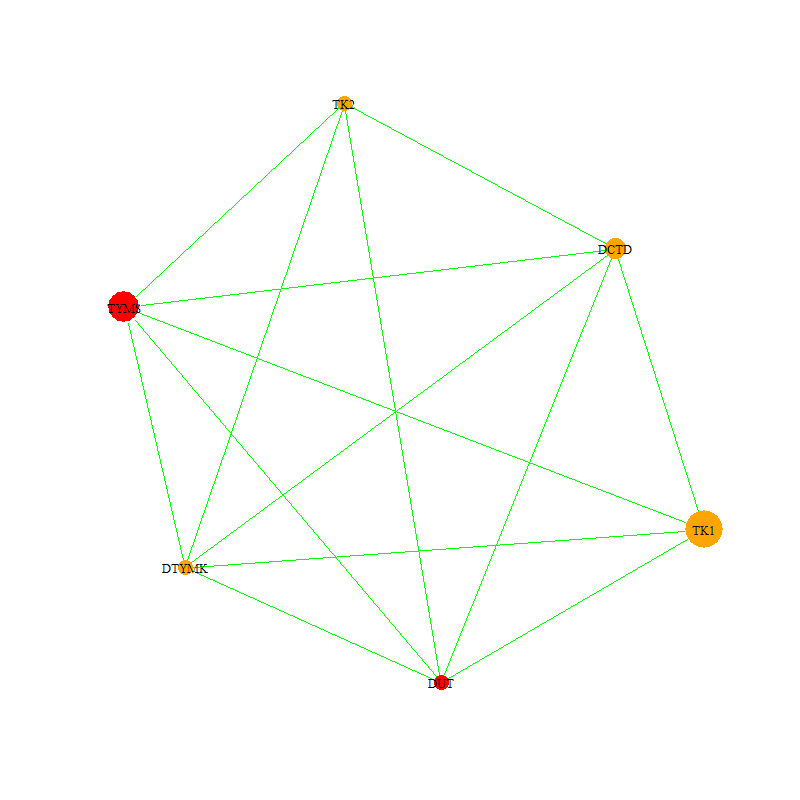

Supplement: Additional file 3 — Visualization of top ranked BC modules. [file 1756-0381-6-17-S3.zip › BCModules/plotBC.173.tif]

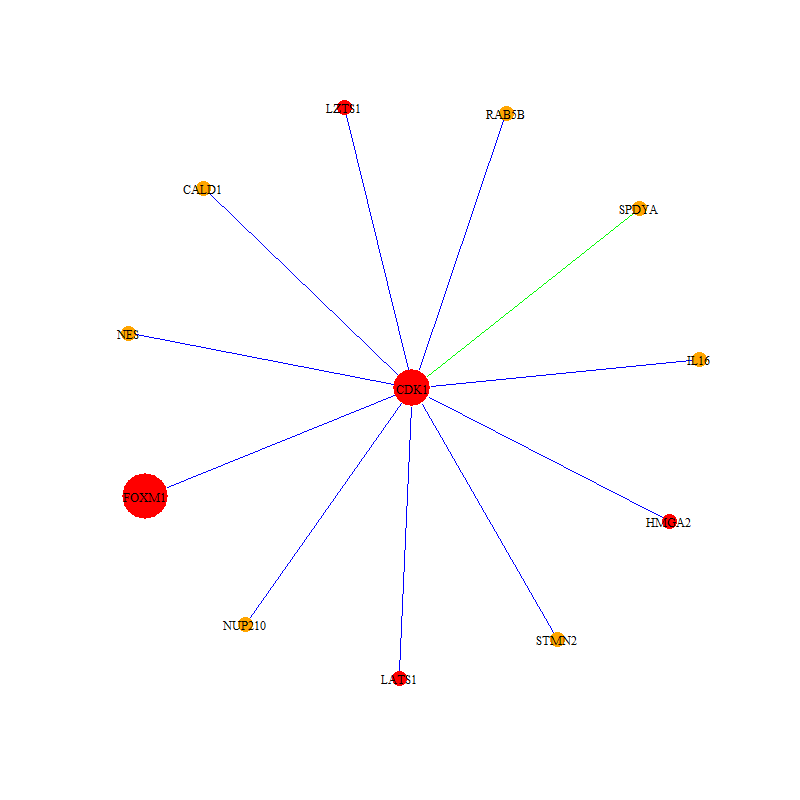

Supplement: Additional file 3 — Visualization of top ranked BC modules. [file 1756-0381-6-17-S3.zip › BCModules/plotBC.182.tif]

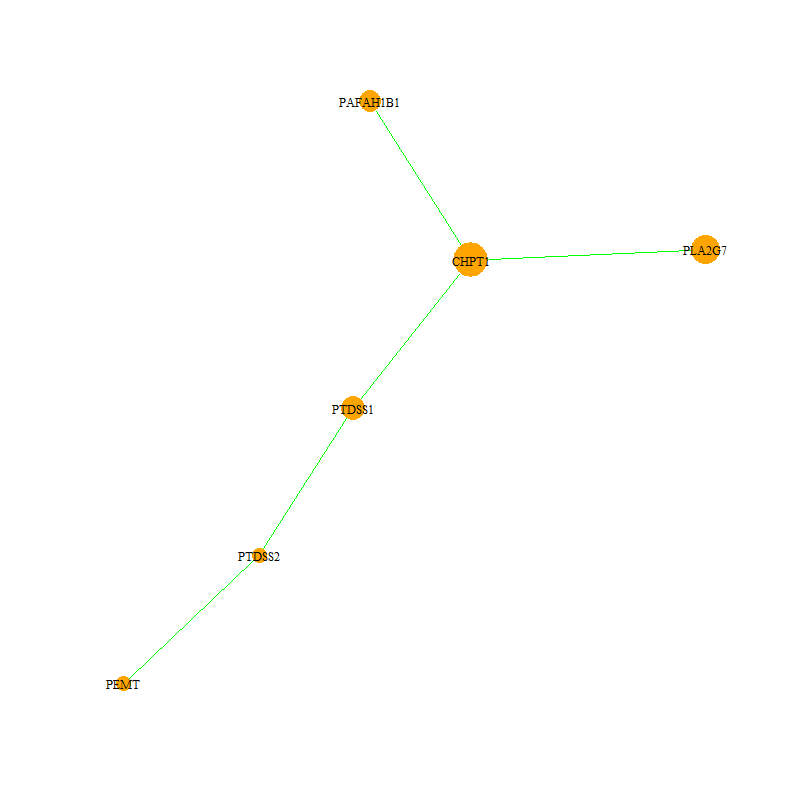

Supplement: Additional file 3 — Visualization of top ranked BC modules. [file 1756-0381-6-17-S3.zip › BCModules/plotBC.184.tif]

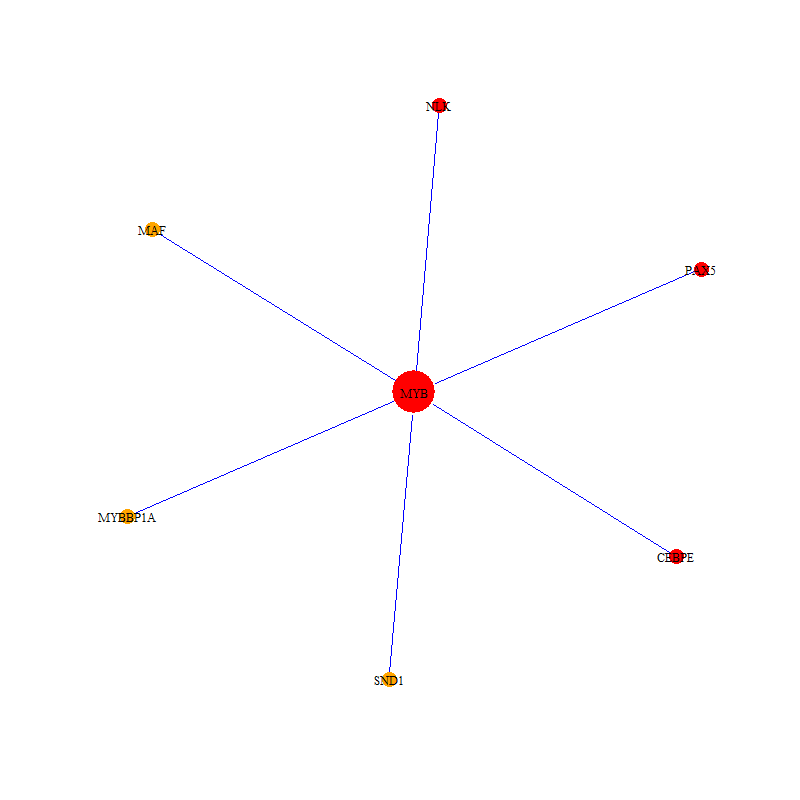

Supplement: Additional file 3 — Visualization of top ranked BC modules. [file 1756-0381-6-17-S3.zip › BCModules/plotBC.189.tif]

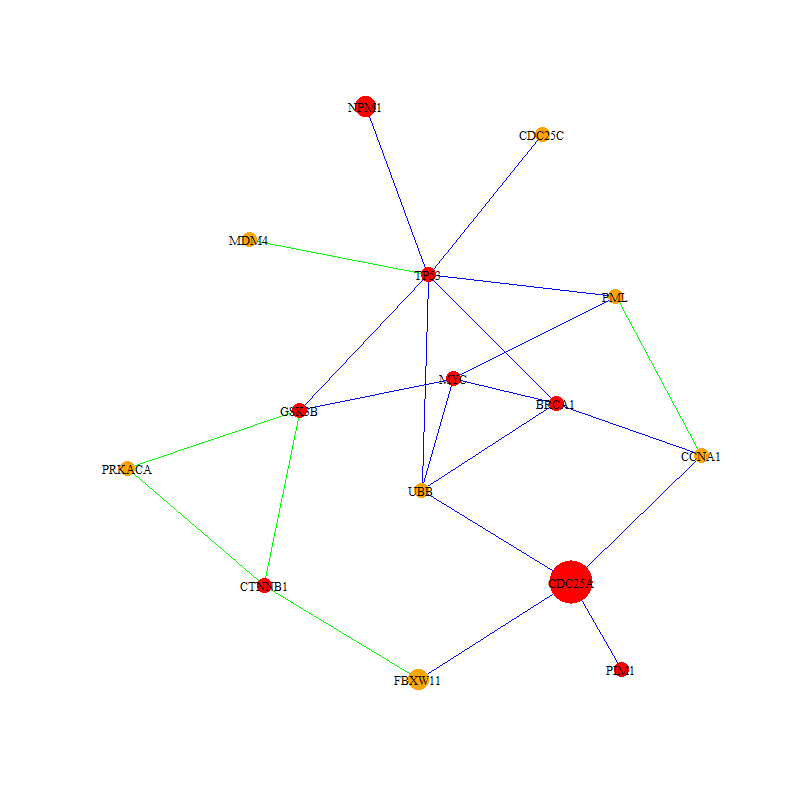

Supplement: Additional file 3 — Visualization of top ranked BC modules. [file 1756-0381-6-17-S3.zip › BCModules/plotBC.205.tif]

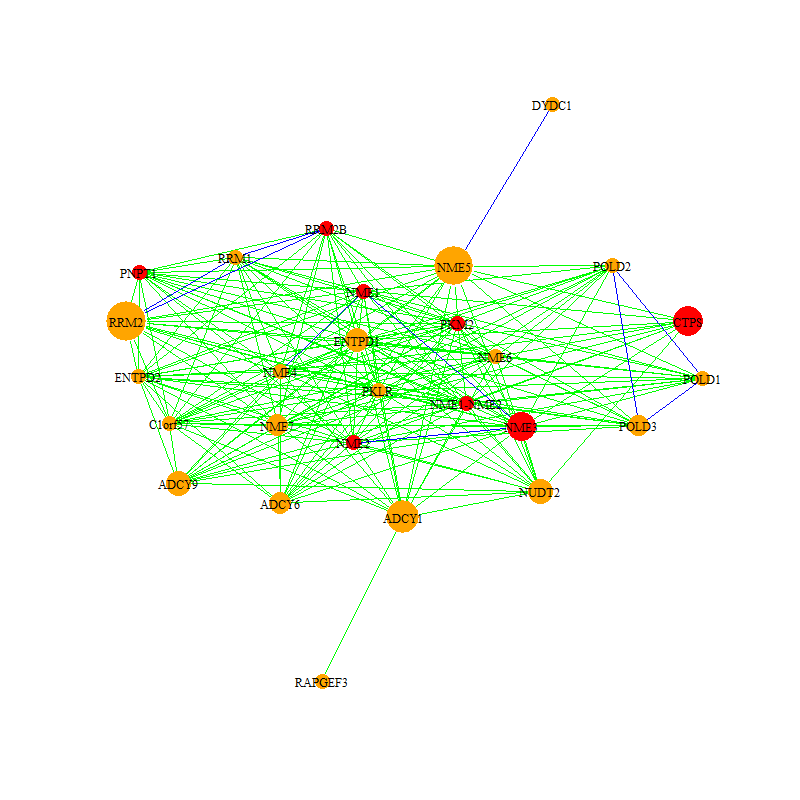

Supplement: Additional file 3 — Visualization of top ranked BC modules. [file 1756-0381-6-17-S3.zip › BCModules/plotBC.224.tif]

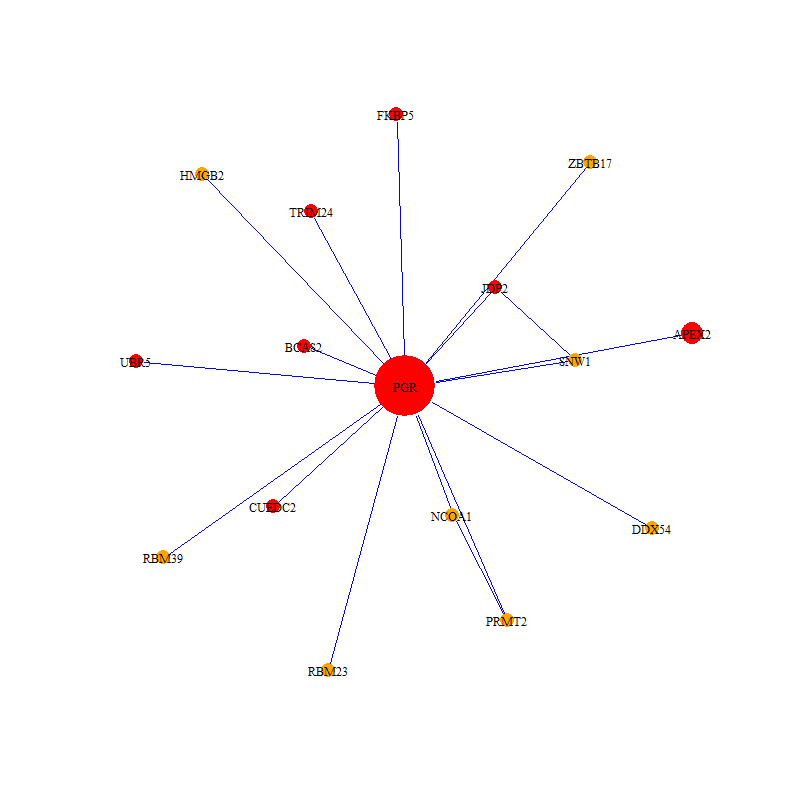

Supplement: Additional file 3 — Visualization of top ranked BC modules. [file 1756-0381-6-17-S3.zip › BCModules/plotBC.226.tif]

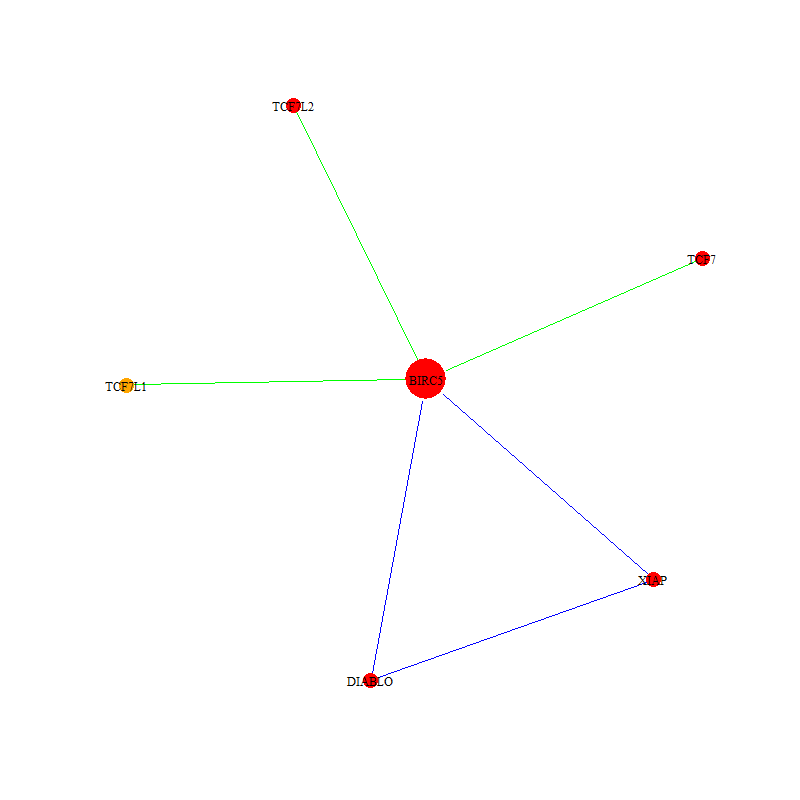

Supplement: Additional file 3 — Visualization of top ranked BC modules. [file 1756-0381-6-17-S3.zip › BCModules/plotBC.242.tif]

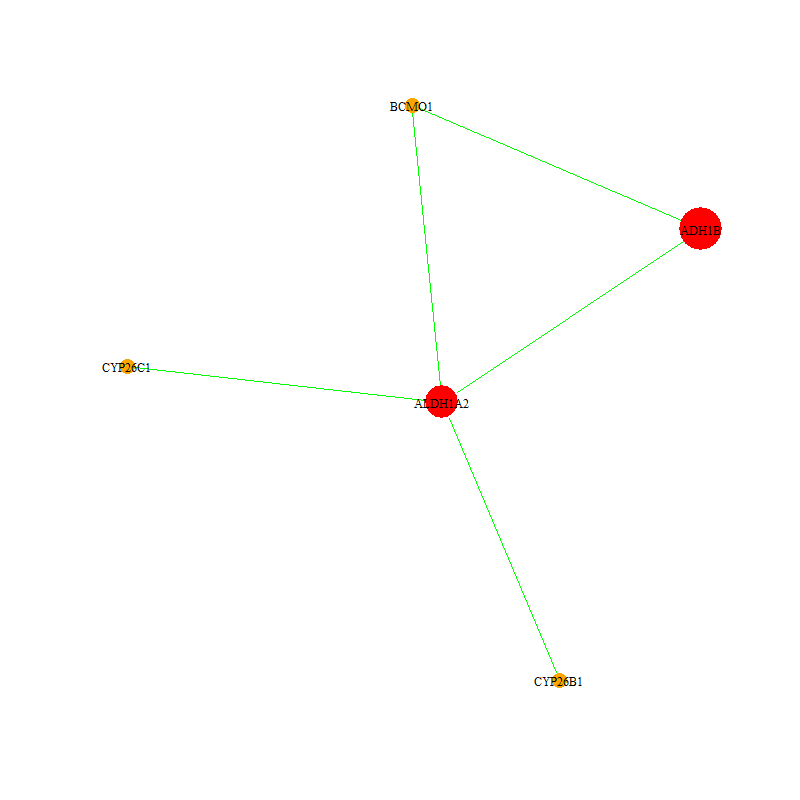

Supplement: Additional file 3 — Visualization of top ranked BC modules. [file 1756-0381-6-17-S3.zip › BCModules/plotBC.321.tif]

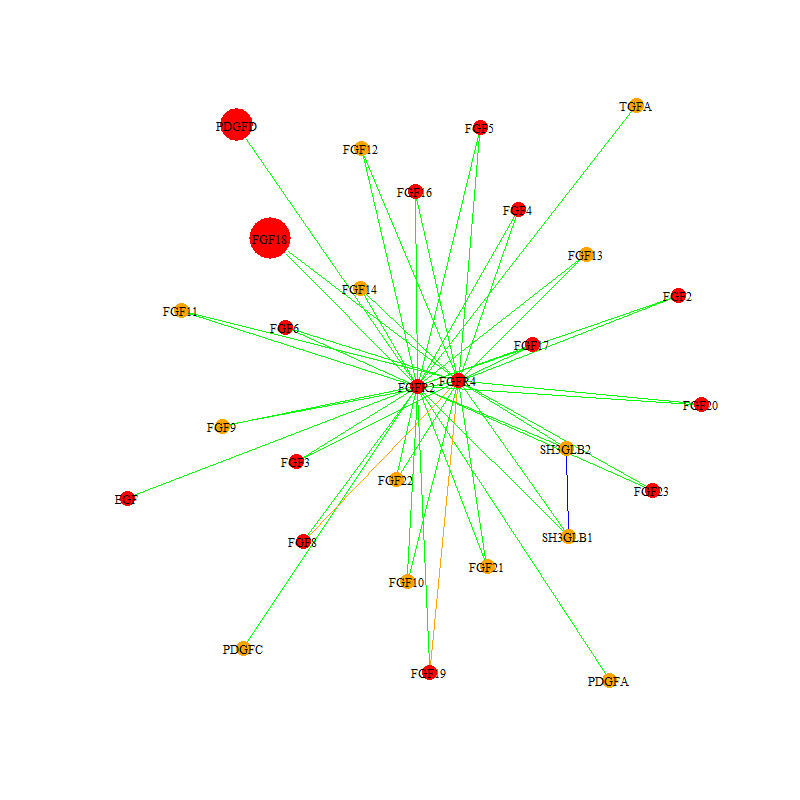

Supplement: Additional file 3 — Visualization of top ranked BC modules. [file 1756-0381-6-17-S3.zip › BCModules/plotBC.348.tif]

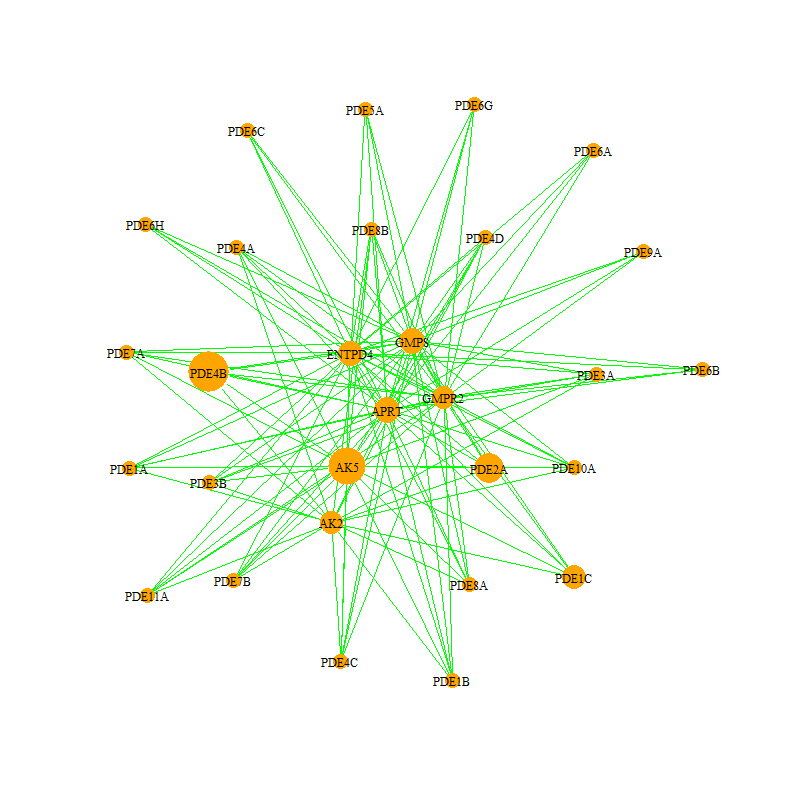

Supplement: Additional file 3 — Visualization of top ranked BC modules. [file 1756-0381-6-17-S3.zip › BCModules/plotBC.395.tif]

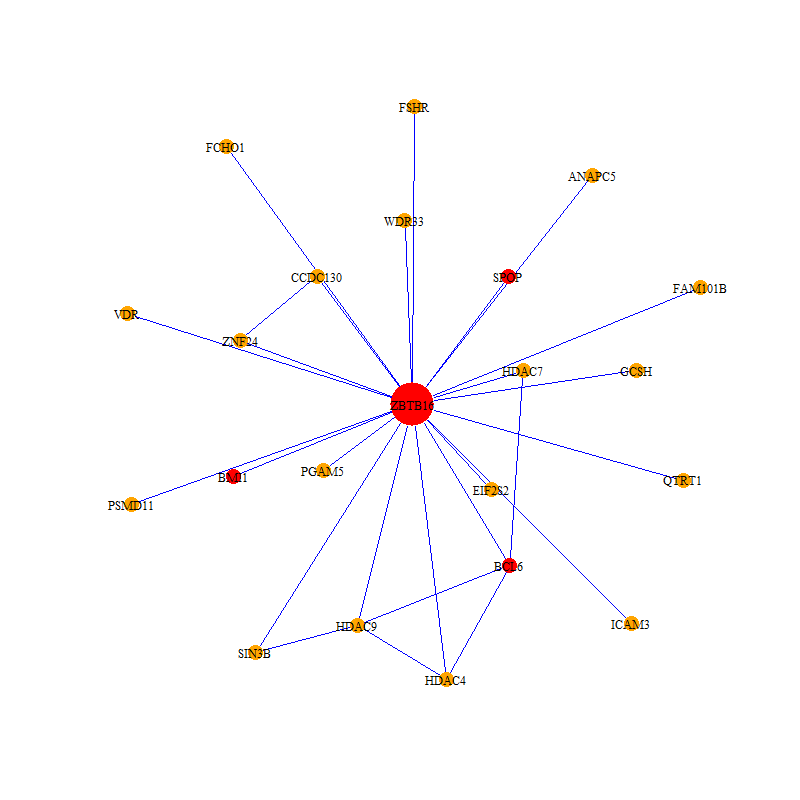

Supplement: Additional file 3 — Visualization of top ranked BC modules. [file 1756-0381-6-17-S3.zip › BCModules/plotBC.49.tif]

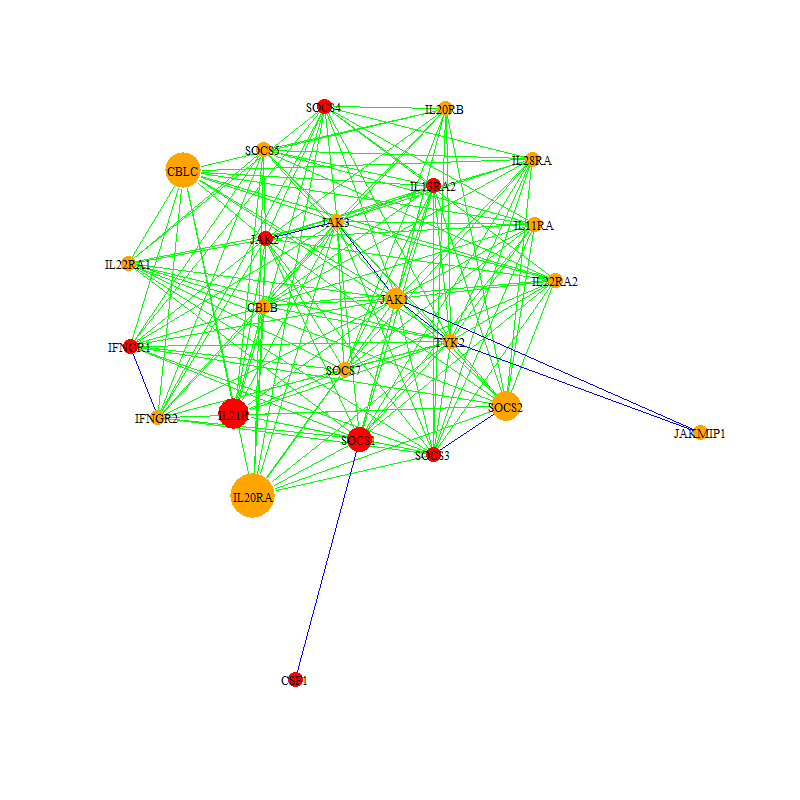

Supplement: Additional file 3 — Visualization of top ranked BC modules. [file 1756-0381-6-17-S3.zip › BCModules/plotBC.79.tif]

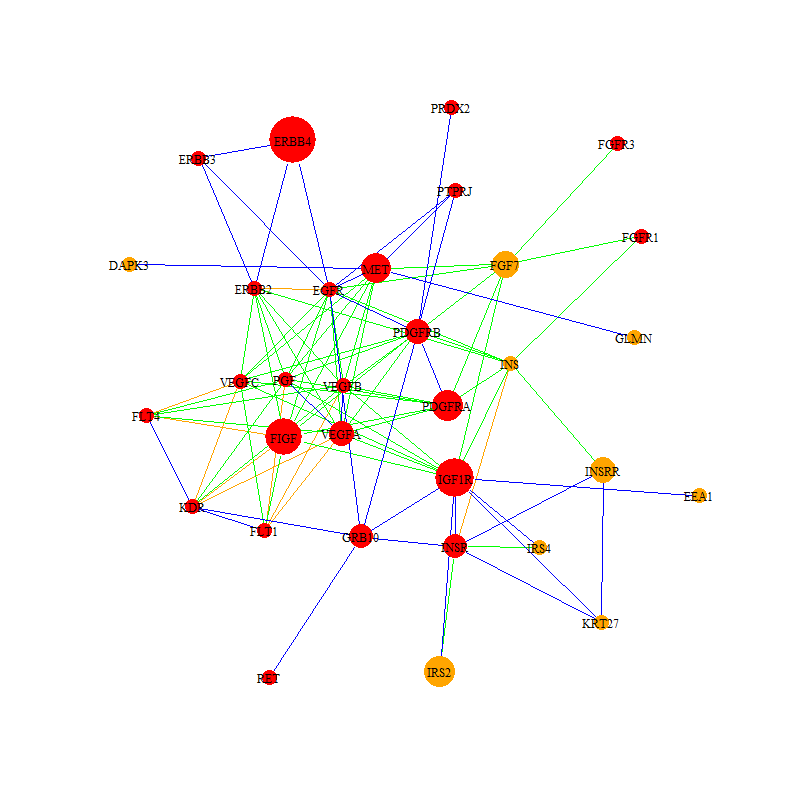

Supplement: Additional file 3 — Visualization of top ranked BC modules. [file 1756-0381-6-17-S3.zip › BCModules/plotBC.82.tif]

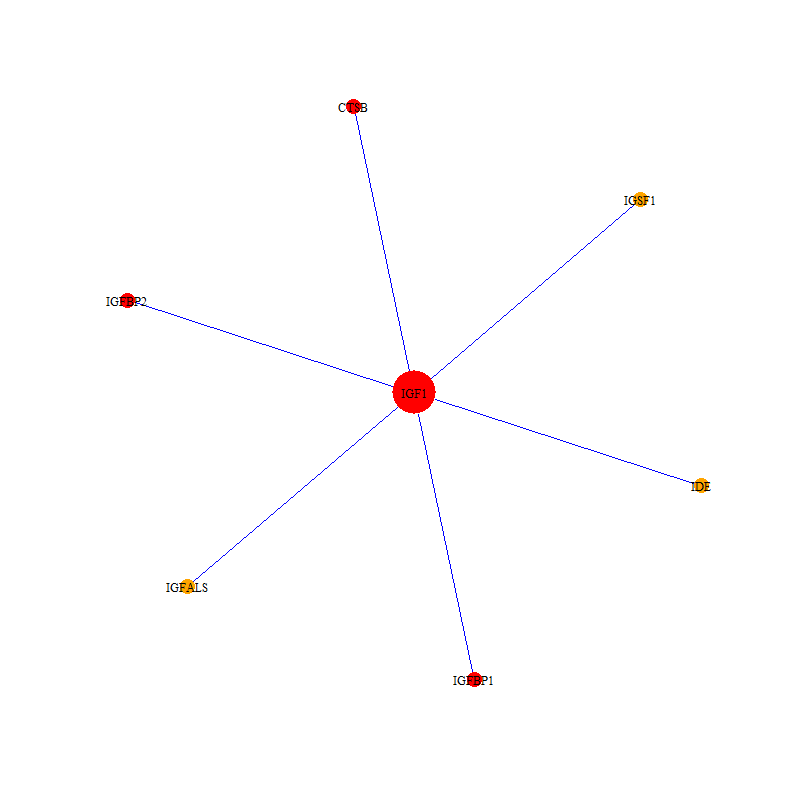

Supplement: Additional file 3 — Visualization of top ranked BC modules. [file 1756-0381-6-17-S3.zip › BCModules/plotBC.89.tif]

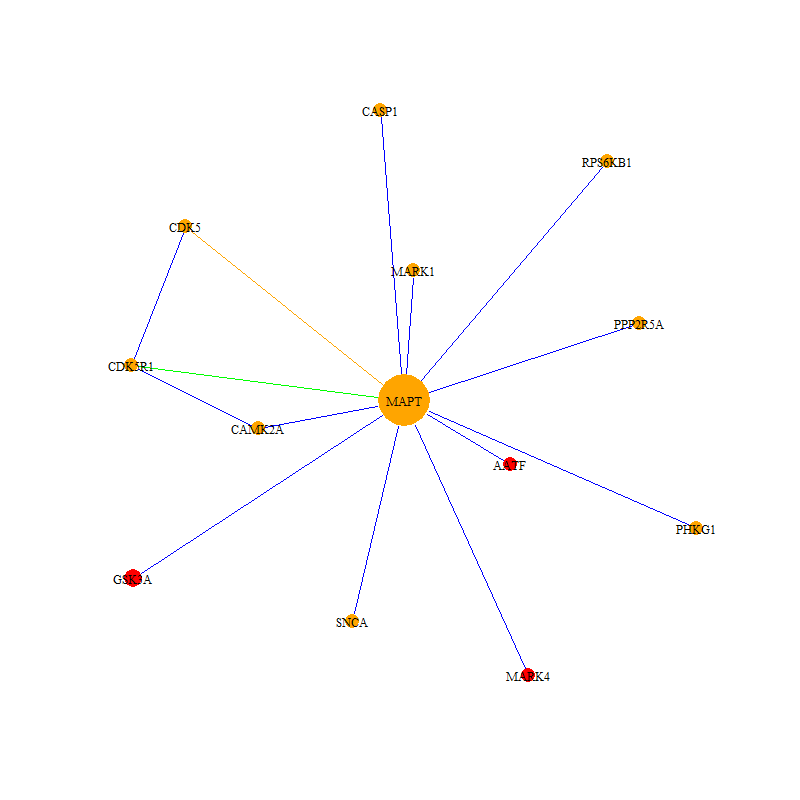

Supplement: Additional file 3 — Visualization of top ranked BC modules. [file 1756-0381-6-17-S3.zip › BCModules/plotBC.96.tif]

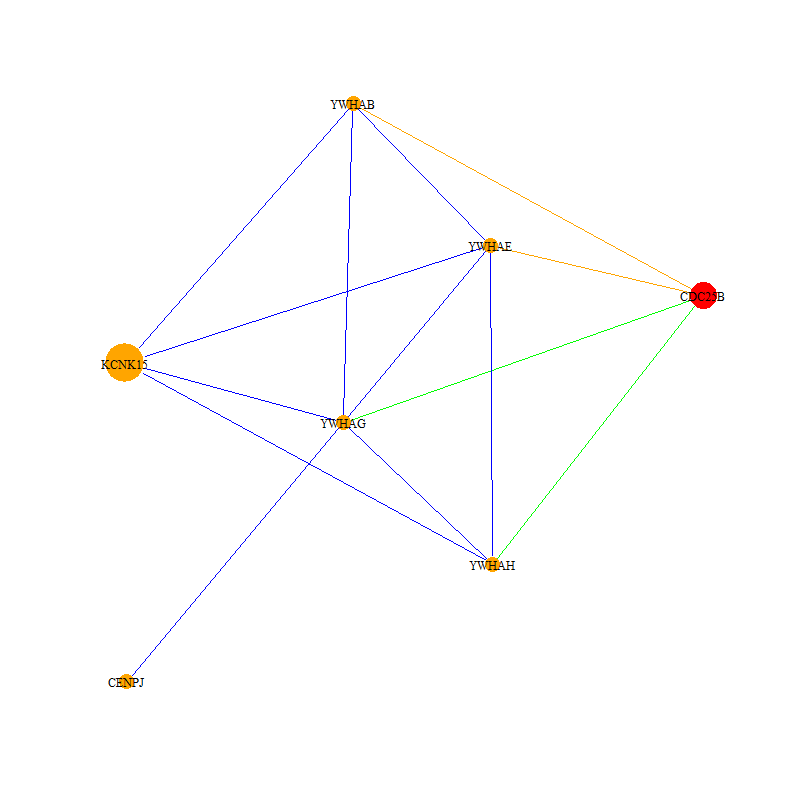

Supplement: Additional file 3 — Visualization of top ranked BC modules. [file 1756-0381-6-17-S3.zip › BCModules/plotBC.99.tif]

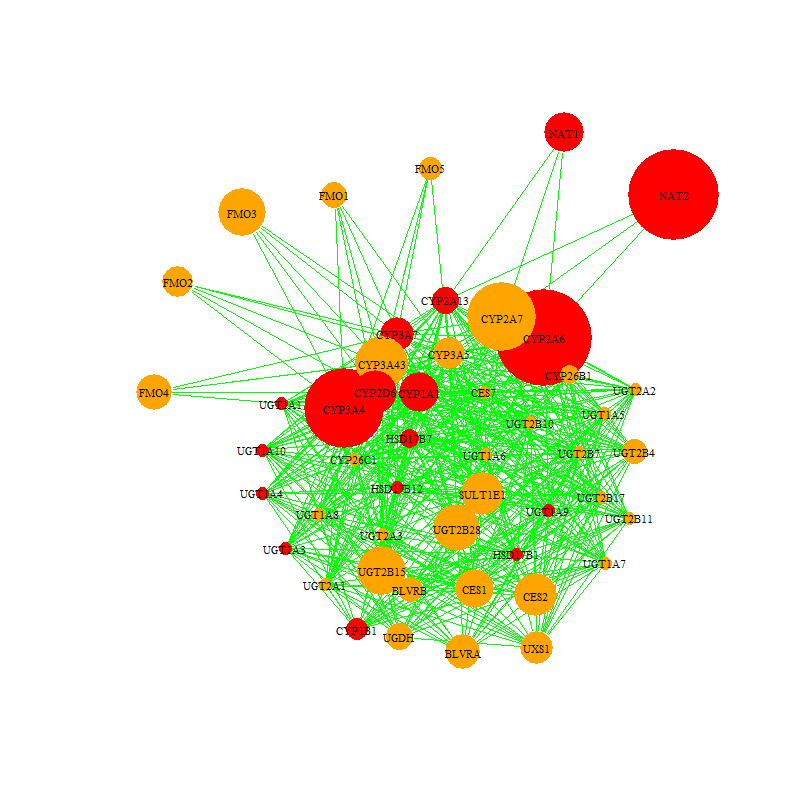

Supplement: Additional file 4 — Visualization of top ranked HCC modules. [file 1756-0381-6-17-S4.zip › HCCModules/plotHCC.10.tif]

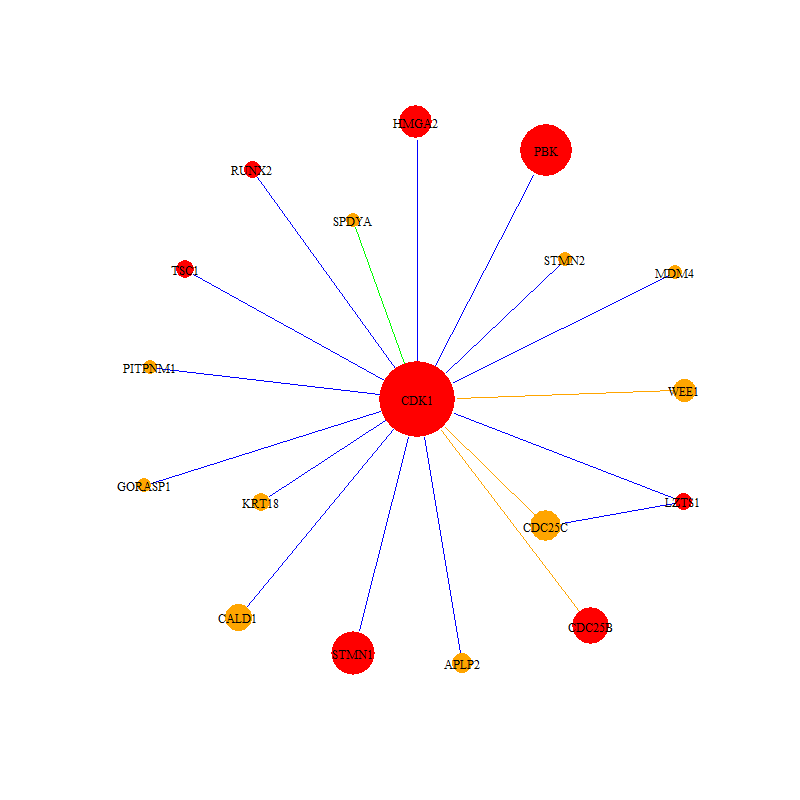

Supplement: Additional file 4 — Visualization of top ranked HCC modules. [file 1756-0381-6-17-S4.zip › HCCModules/plotHCC.100.tif]

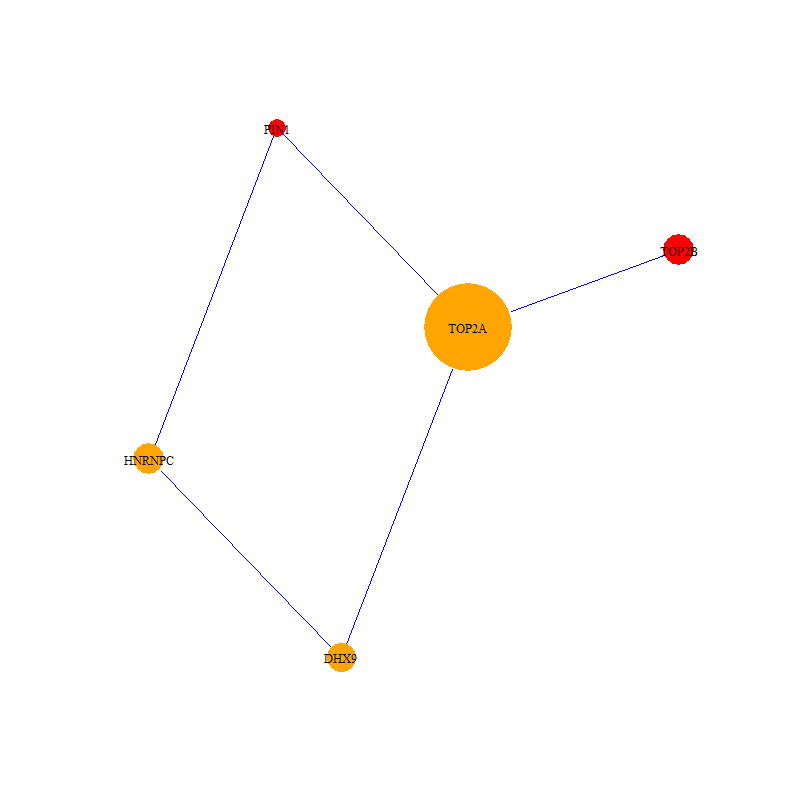

Supplement: Additional file 4 — Visualization of top ranked HCC modules. [file 1756-0381-6-17-S4.zip › HCCModules/plotHCC.112.tif]

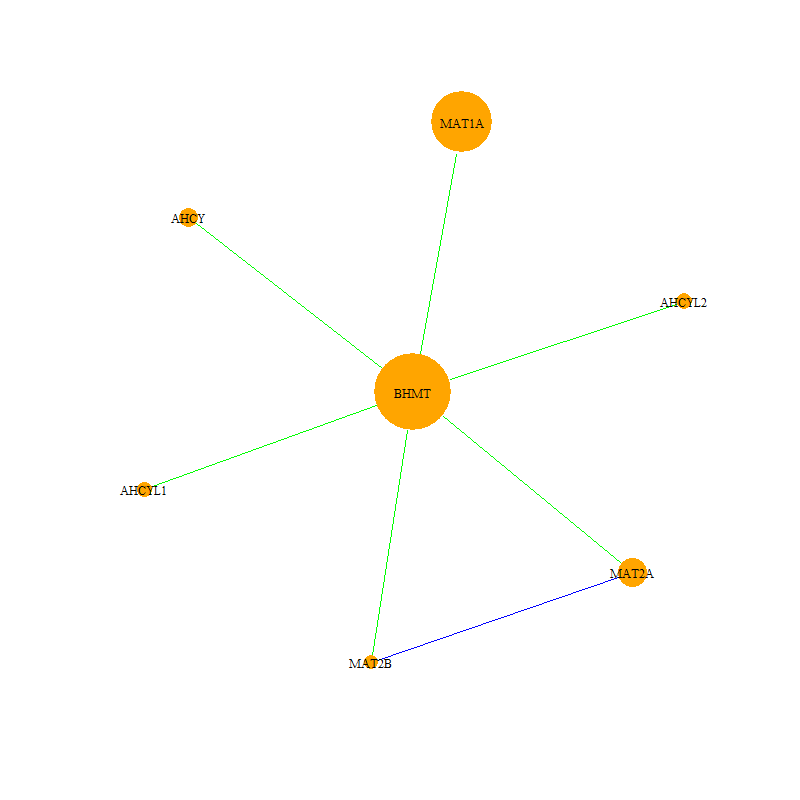

Supplement: Additional file 4 — Visualization of top ranked HCC modules. [file 1756-0381-6-17-S4.zip › HCCModules/plotHCC.123.tif]

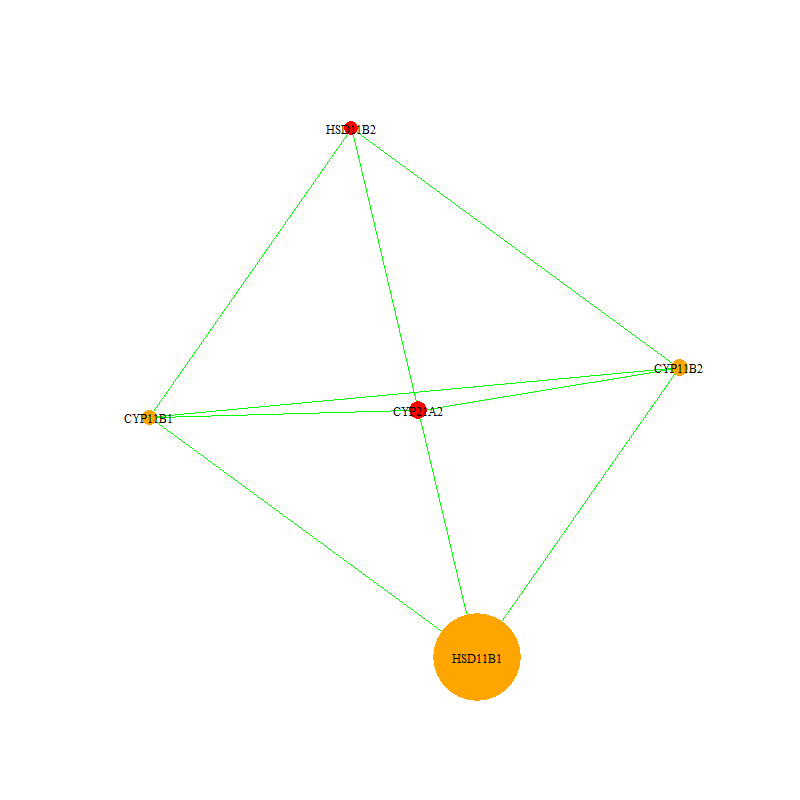

Supplement: Additional file 4 — Visualization of top ranked HCC modules. [file 1756-0381-6-17-S4.zip › HCCModules/plotHCC.153.tif]

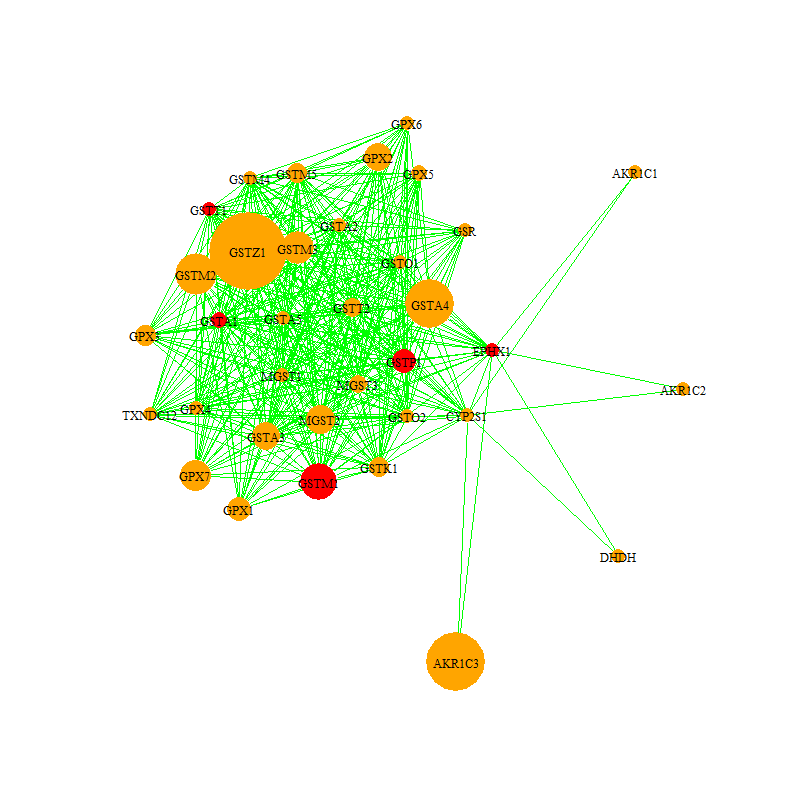

Supplement: Additional file 4 — Visualization of top ranked HCC modules. [file 1756-0381-6-17-S4.zip › HCCModules/plotHCC.220.tif]

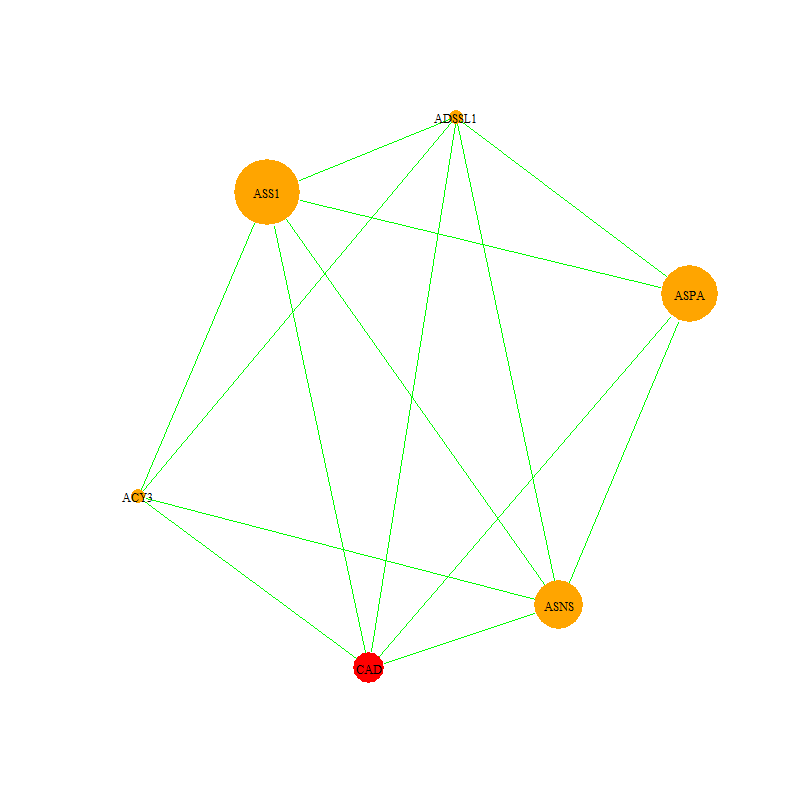

Supplement: Additional file 4 — Visualization of top ranked HCC modules. [file 1756-0381-6-17-S4.zip › HCCModules/plotHCC.254.tif]

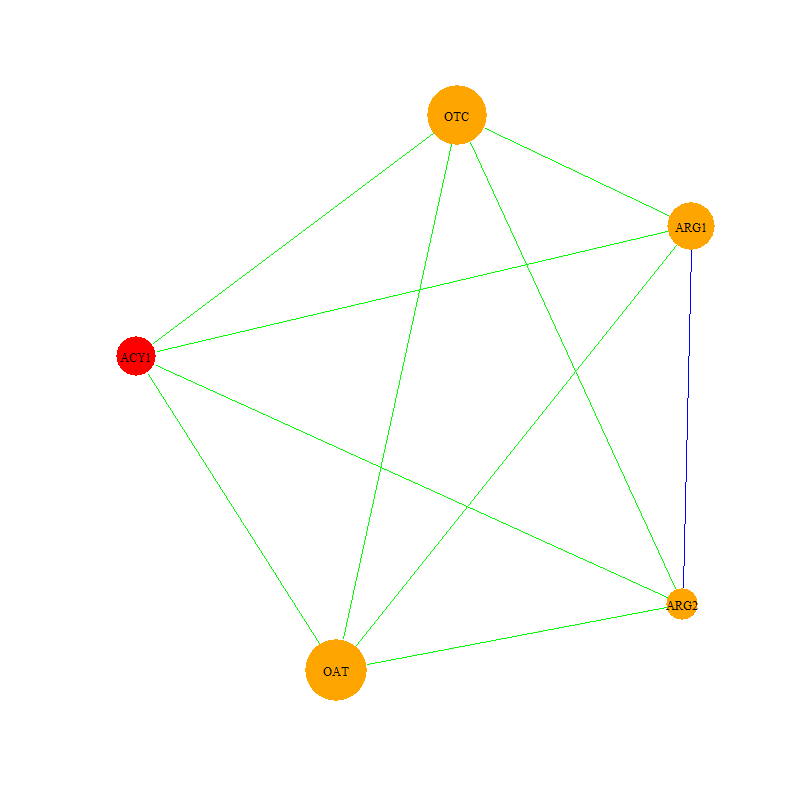

Supplement: Additional file 4 — Visualization of top ranked HCC modules. [file 1756-0381-6-17-S4.zip › HCCModules/plotHCC.257.tif]

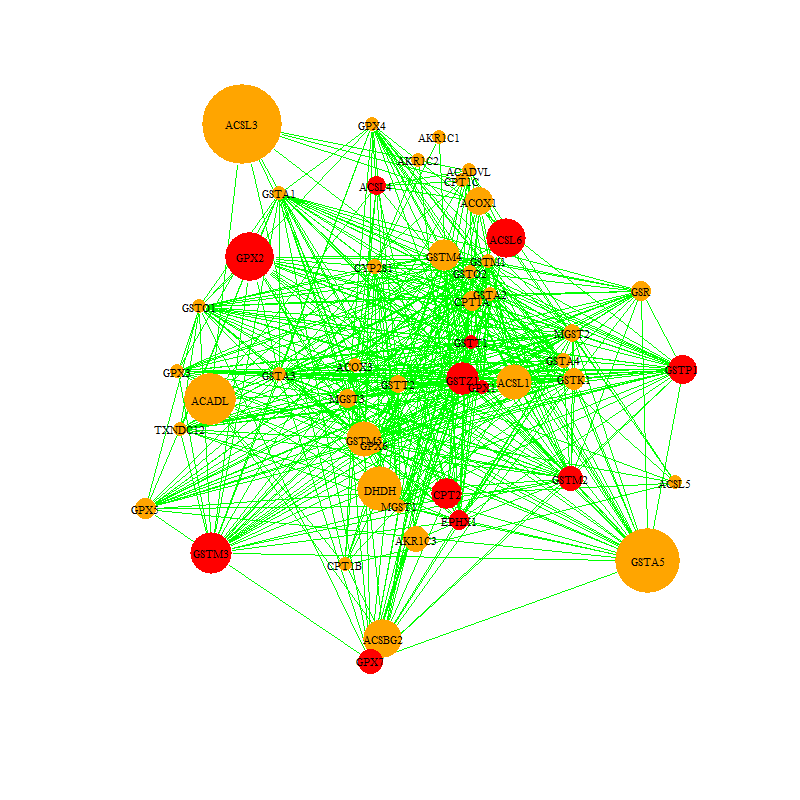

Supplement: Additional file 4 — Visualization of top ranked HCC modules. [file 1756-0381-6-17-S4.zip › HCCModules/plotHCC.31.tif]

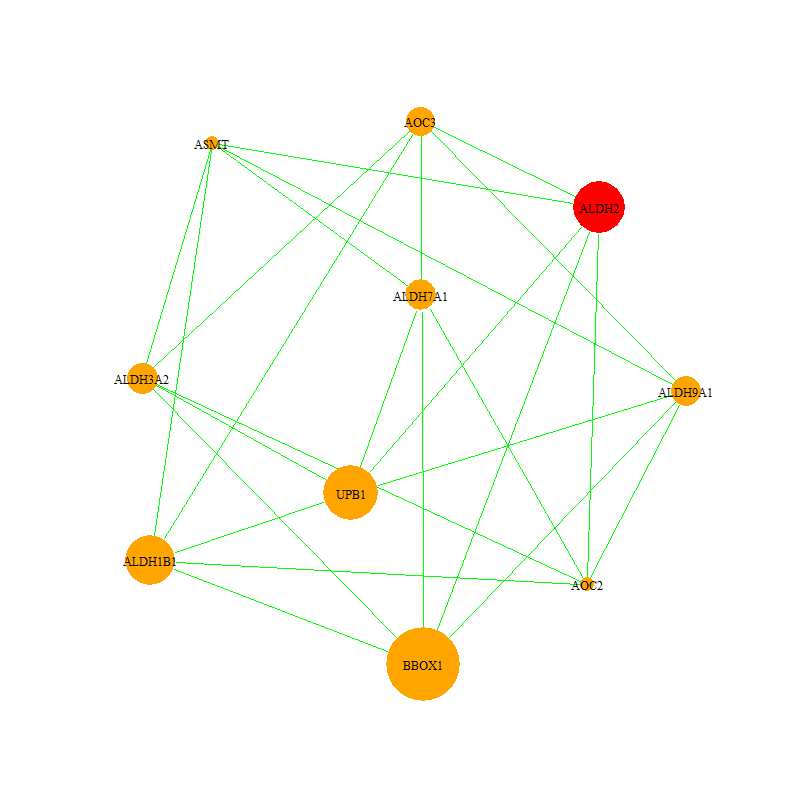

Supplement: Additional file 4 — Visualization of top ranked HCC modules. [file 1756-0381-6-17-S4.zip › HCCModules/plotHCC.314.tif]

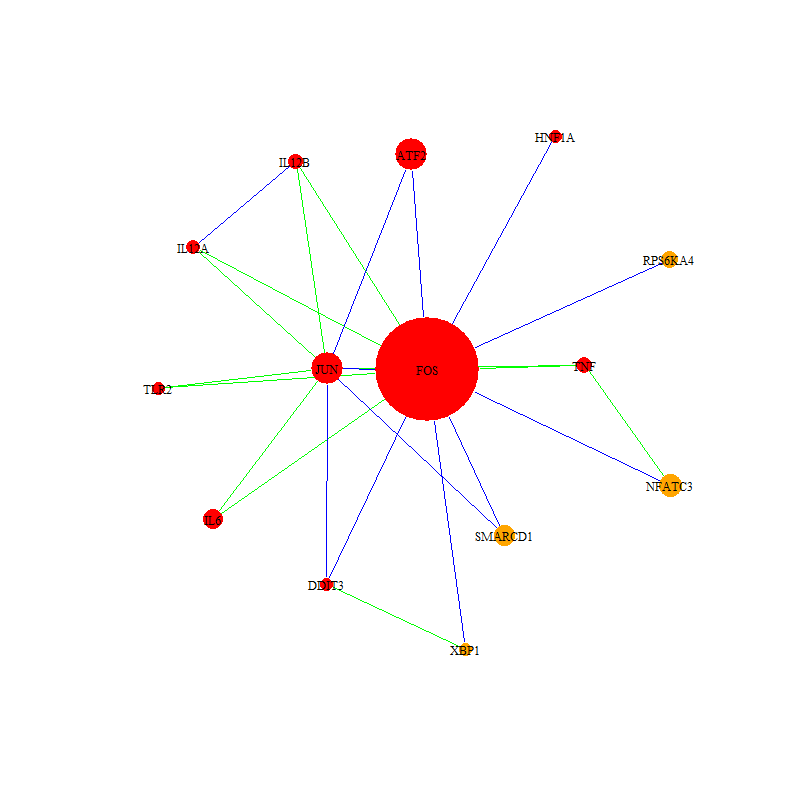

Supplement: Additional file 4 — Visualization of top ranked HCC modules. [file 1756-0381-6-17-S4.zip › HCCModules/plotHCC.34.tif]

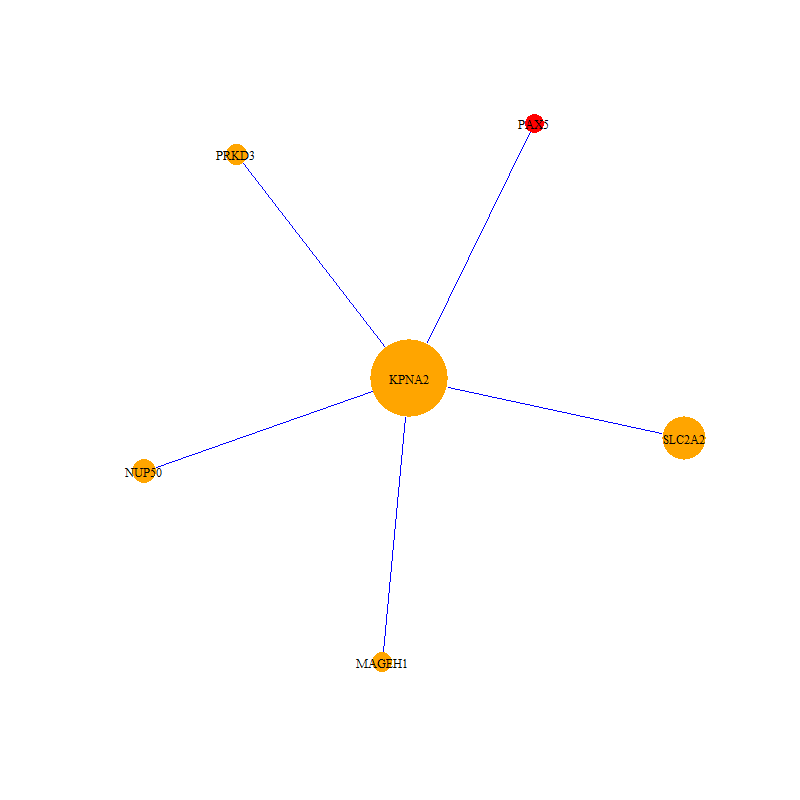

Supplement: Additional file 4 — Visualization of top ranked HCC modules. [file 1756-0381-6-17-S4.zip › HCCModules/plotHCC.36.tif]

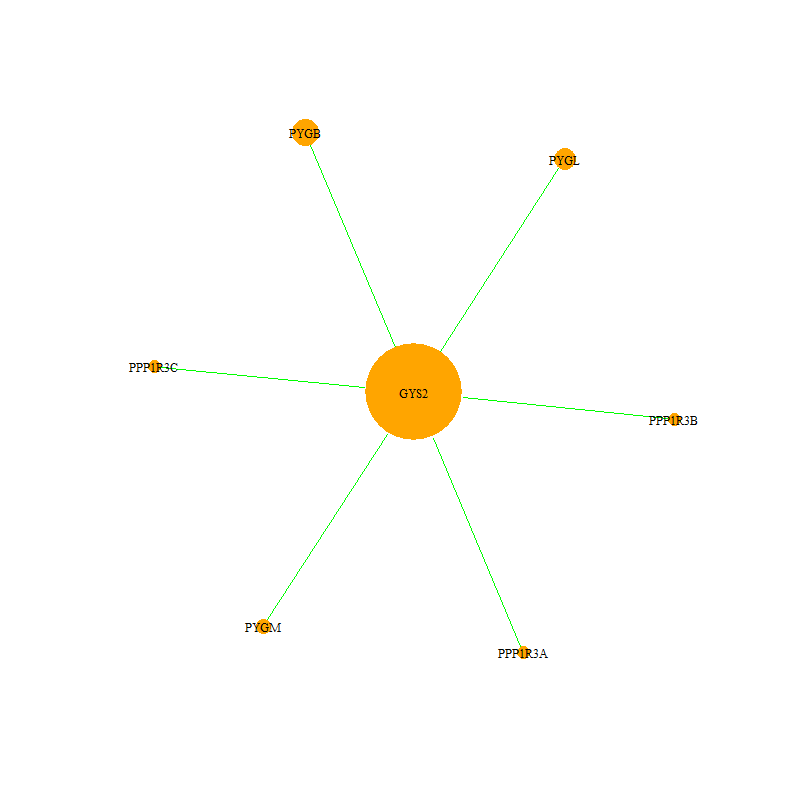

Supplement: Additional file 4 — Visualization of top ranked HCC modules. [file 1756-0381-6-17-S4.zip › HCCModules/plotHCC.360.tif]

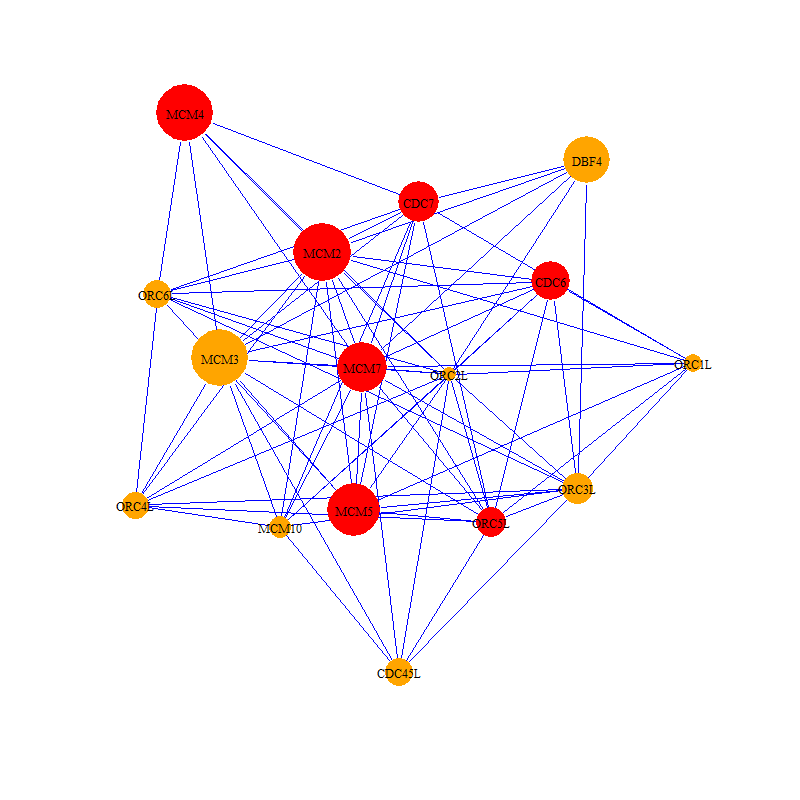

Supplement: Additional file 4 — Visualization of top ranked HCC modules. [file 1756-0381-6-17-S4.zip › HCCModules/plotHCC.361.tif]

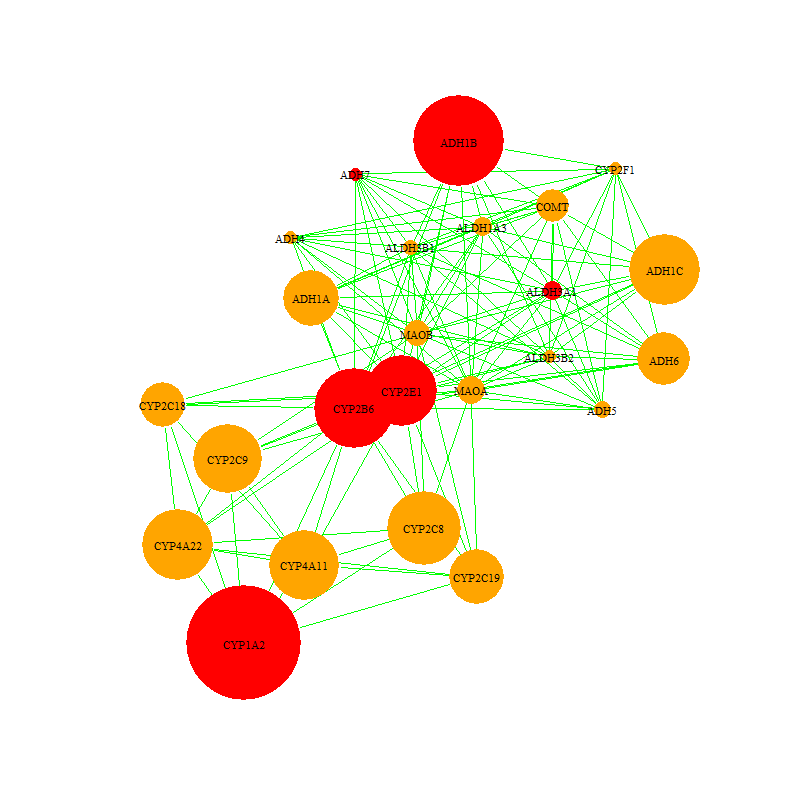

Supplement: Additional file 4 — Visualization of top ranked HCC modules. [file 1756-0381-6-17-S4.zip › HCCModules/plotHCC.408.tif]

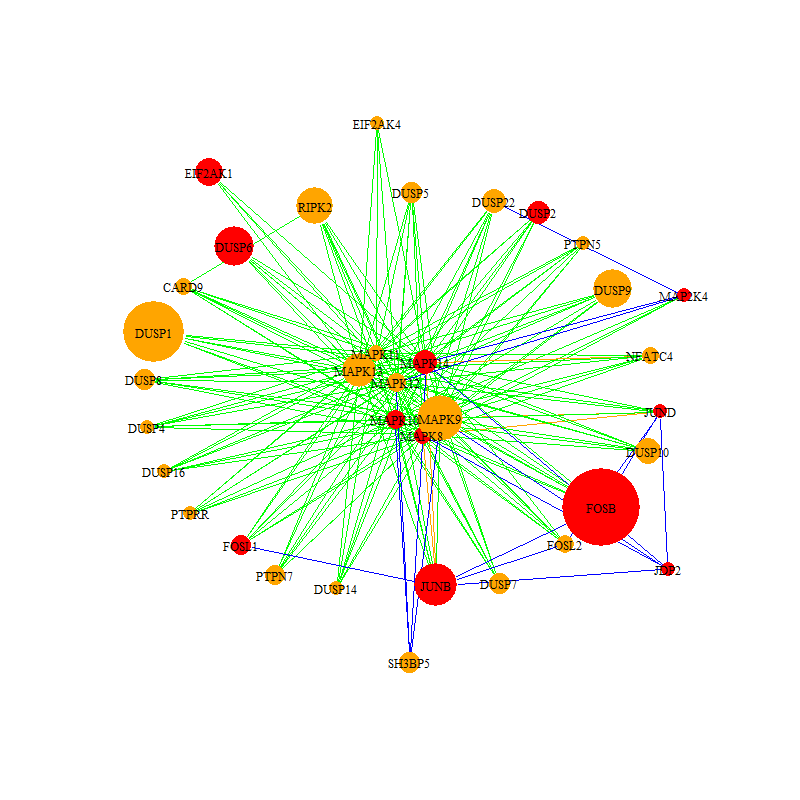

Supplement: Additional file 4 — Visualization of top ranked HCC modules. [file 1756-0381-6-17-S4.zip › HCCModules/plotHCC.414.tif]

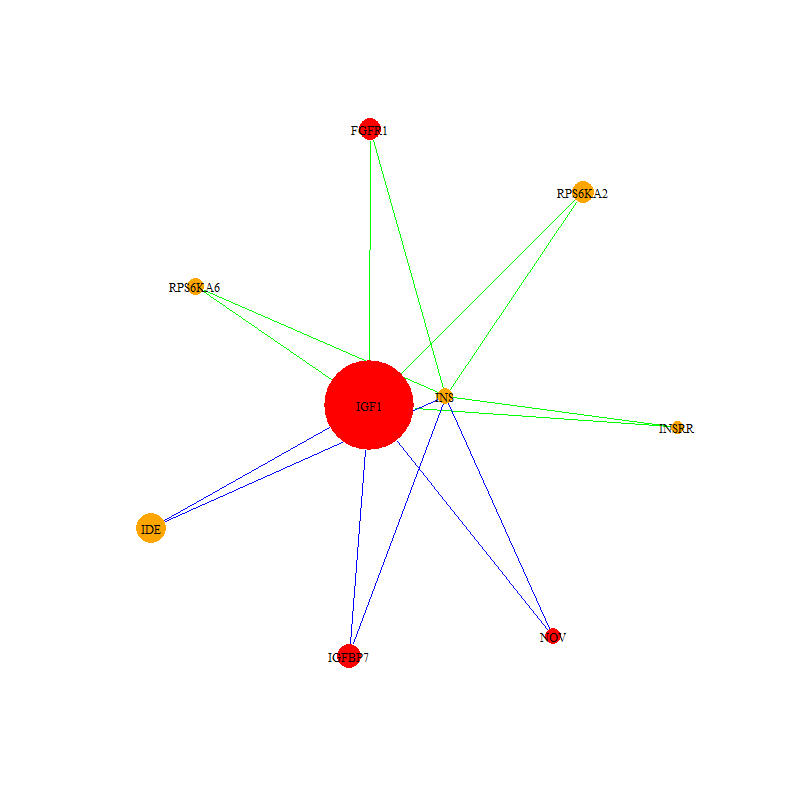

Supplement: Additional file 4 — Visualization of top ranked HCC modules. [file 1756-0381-6-17-S4.zip › HCCModules/plotHCC.429.tif]

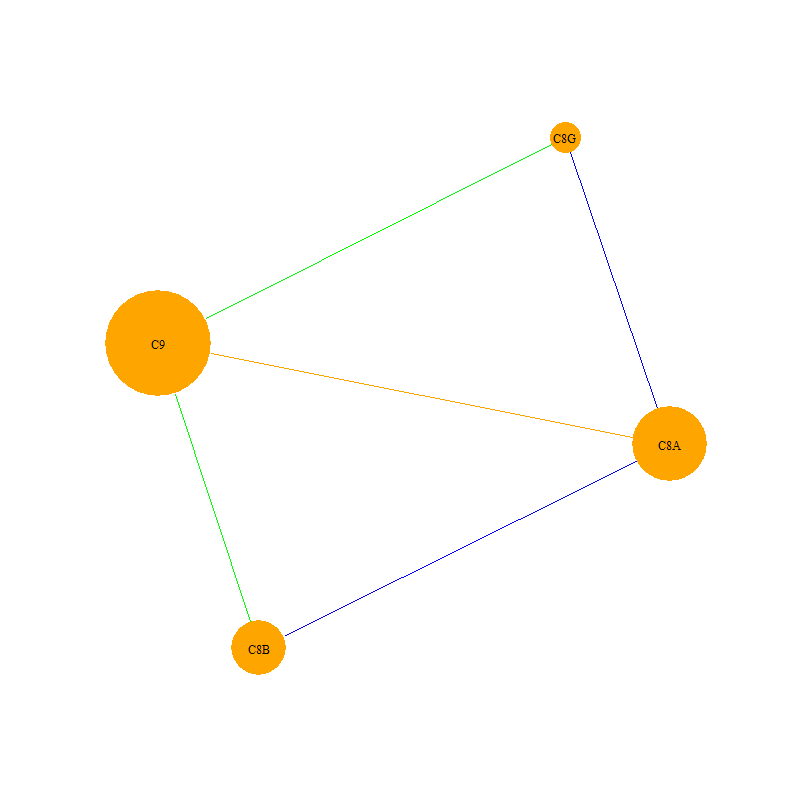

Supplement: Additional file 4 — Visualization of top ranked HCC modules. [file 1756-0381-6-17-S4.zip › HCCModules/plotHCC.513.tif]

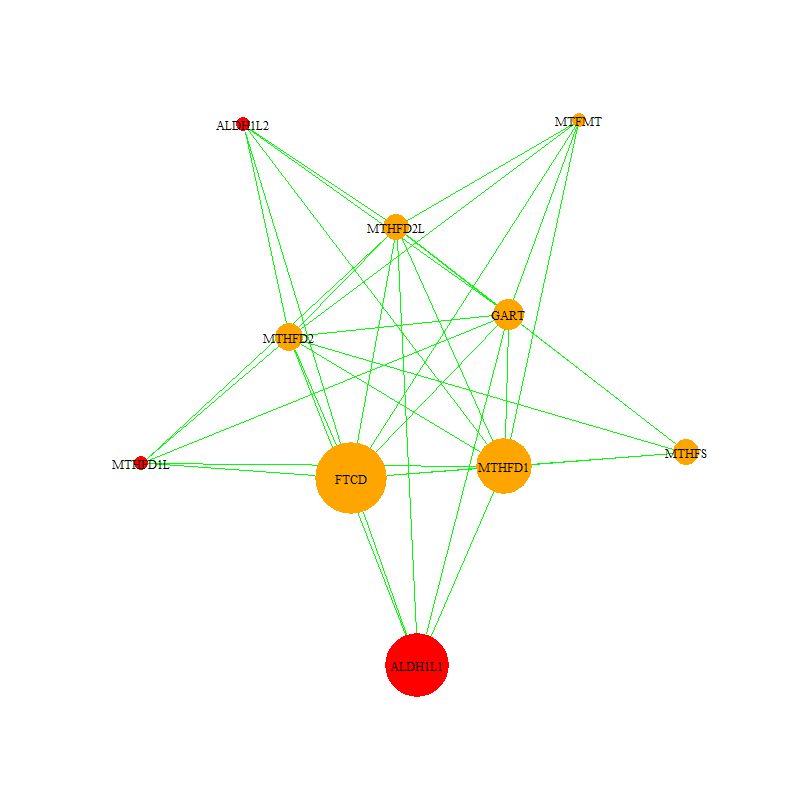

Supplement: Additional file 4 — Visualization of top ranked HCC modules. [file 1756-0381-6-17-S4.zip › HCCModules/plotHCC.515.tif]

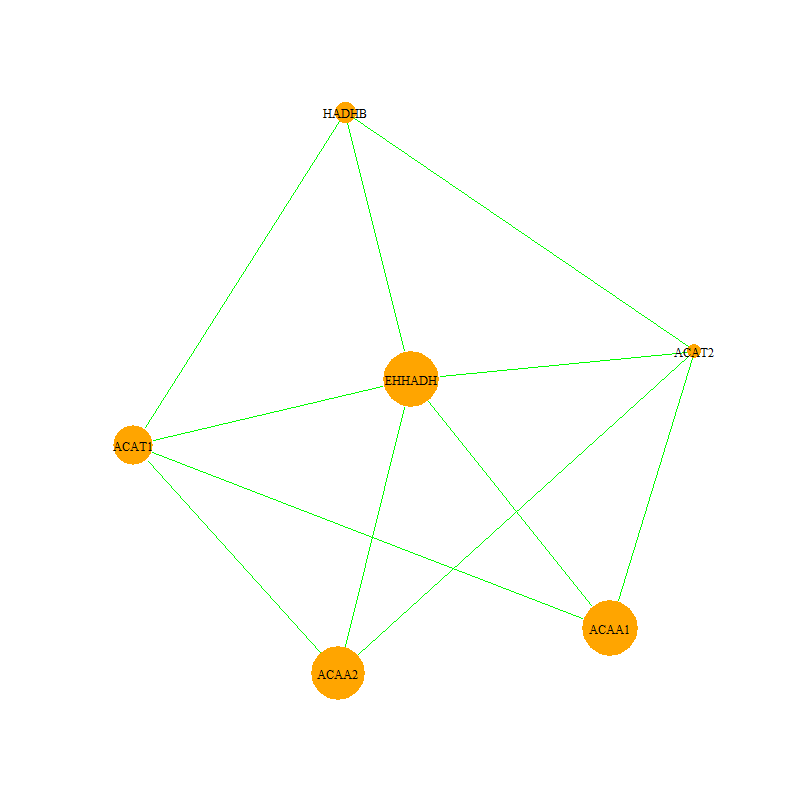

Supplement: Additional file 4 — Visualization of top ranked HCC modules. [file 1756-0381-6-17-S4.zip › HCCModules/plotHCC.524.tif]

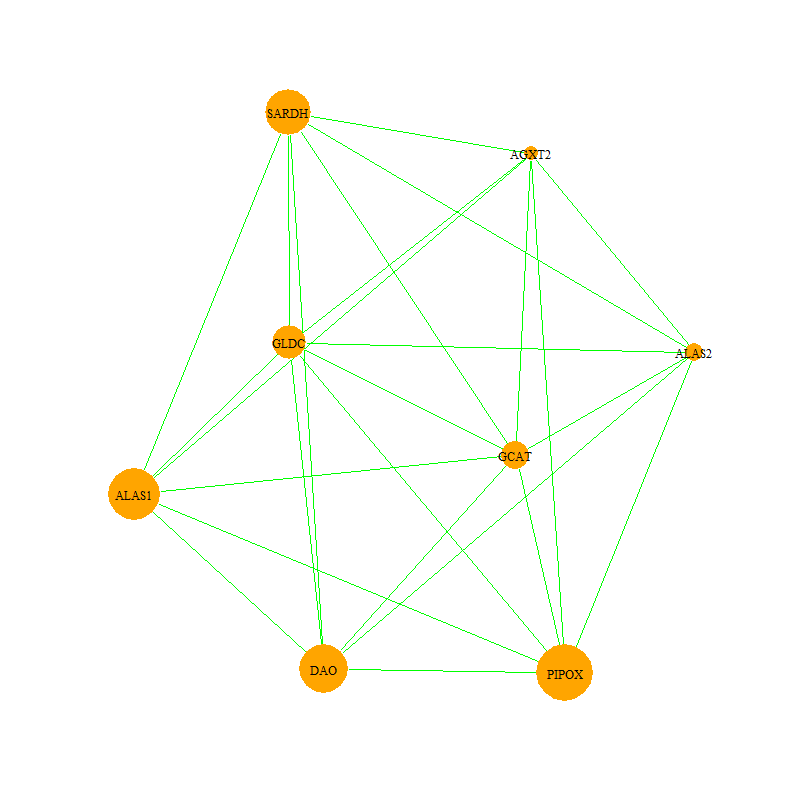

Supplement: Additional file 4 — Visualization of top ranked HCC modules. [file 1756-0381-6-17-S4.zip › HCCModules/plotHCC.578.tif]

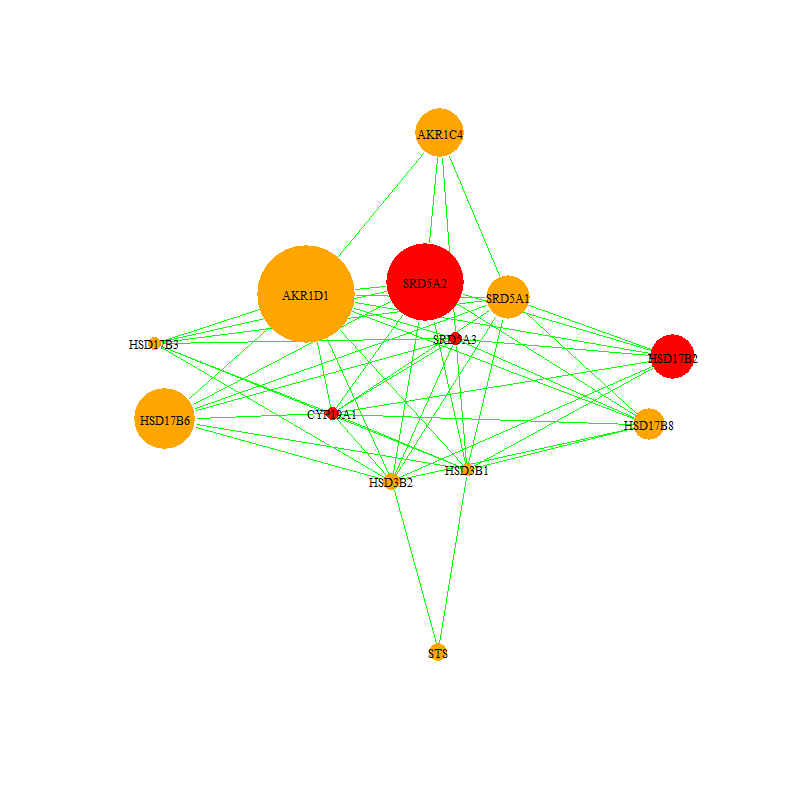

Supplement: Additional file 4 — Visualization of top ranked HCC modules. [file 1756-0381-6-17-S4.zip › HCCModules/plotHCC.579.tif]

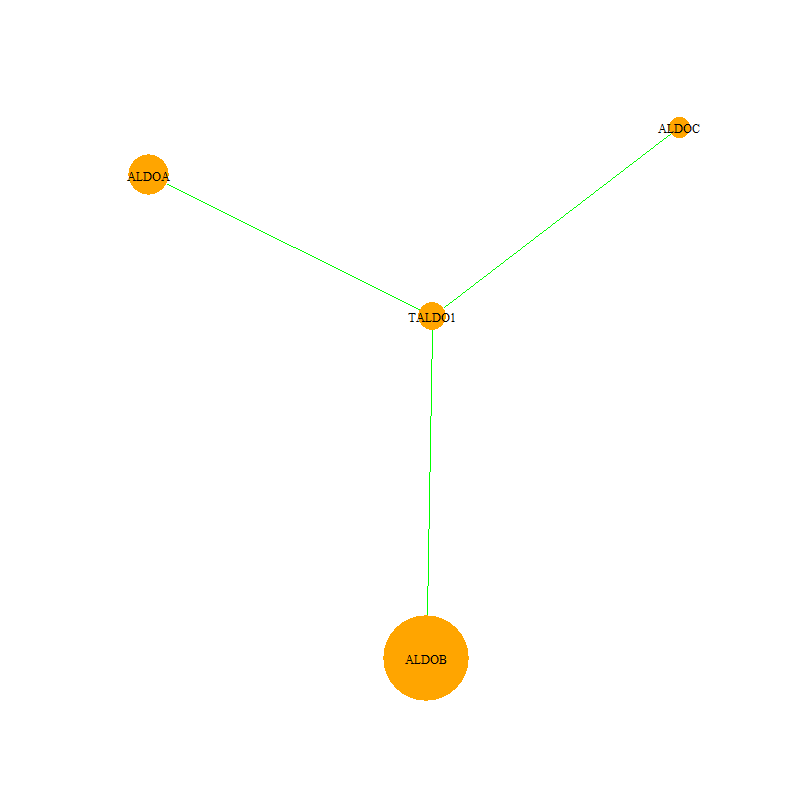

Supplement: Additional file 4 — Visualization of top ranked HCC modules. [file 1756-0381-6-17-S4.zip › HCCModules/plotHCC.598.tif]

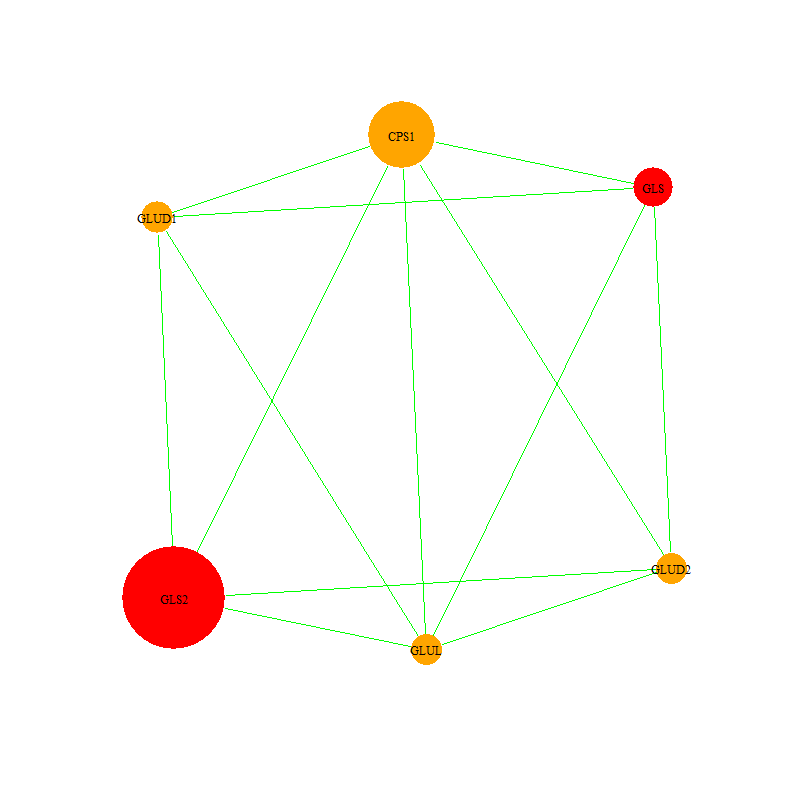

Supplement: Additional file 4 — Visualization of top ranked HCC modules. [file 1756-0381-6-17-S4.zip › HCCModules/plotHCC.603.tif]

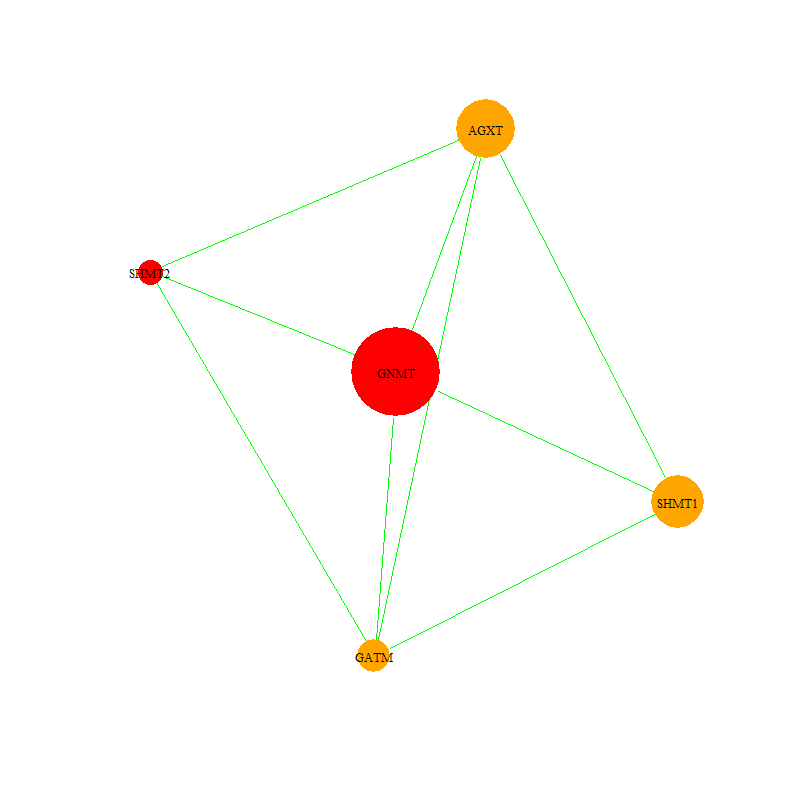

Supplement: Additional file 4 — Visualization of top ranked HCC modules. [file 1756-0381-6-17-S4.zip › HCCModules/plotHCC.97.tif]

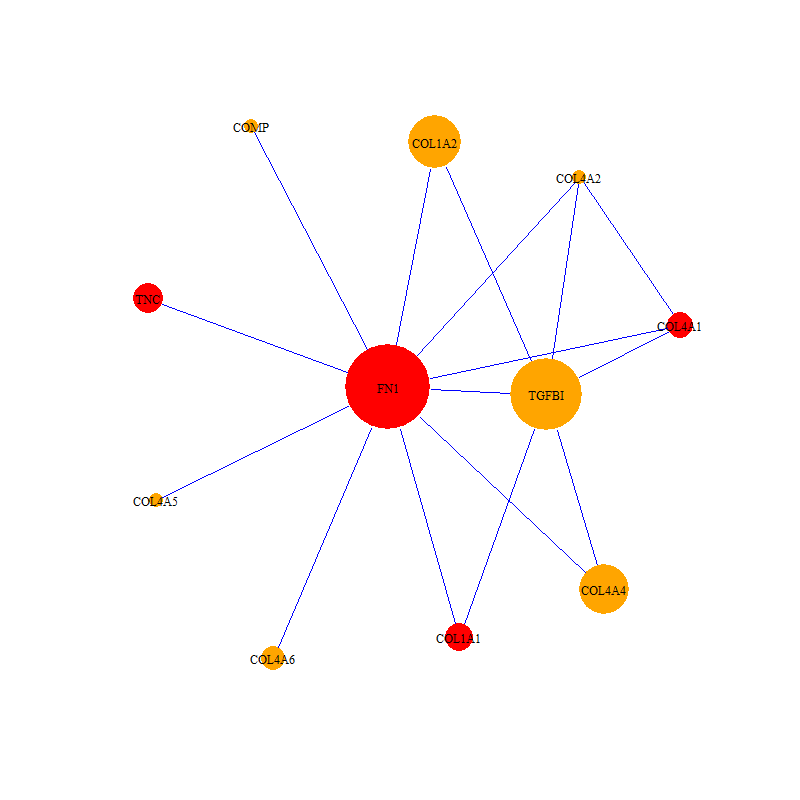

Supplement: Additional file 5 — Visualization of top ranked CCA modules. [file 1756-0381-6-17-S5.zip › CCAModules/plotCCA.111.tif]

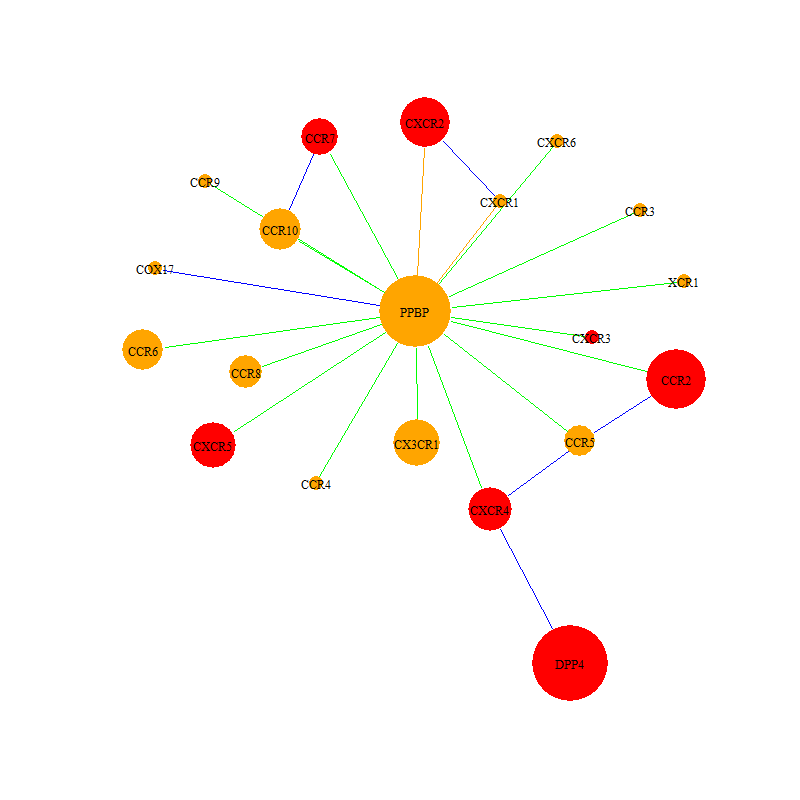

Supplement: Additional file 5 — Visualization of top ranked CCA modules. [file 1756-0381-6-17-S5.zip › CCAModules/plotCCA.125.tif]

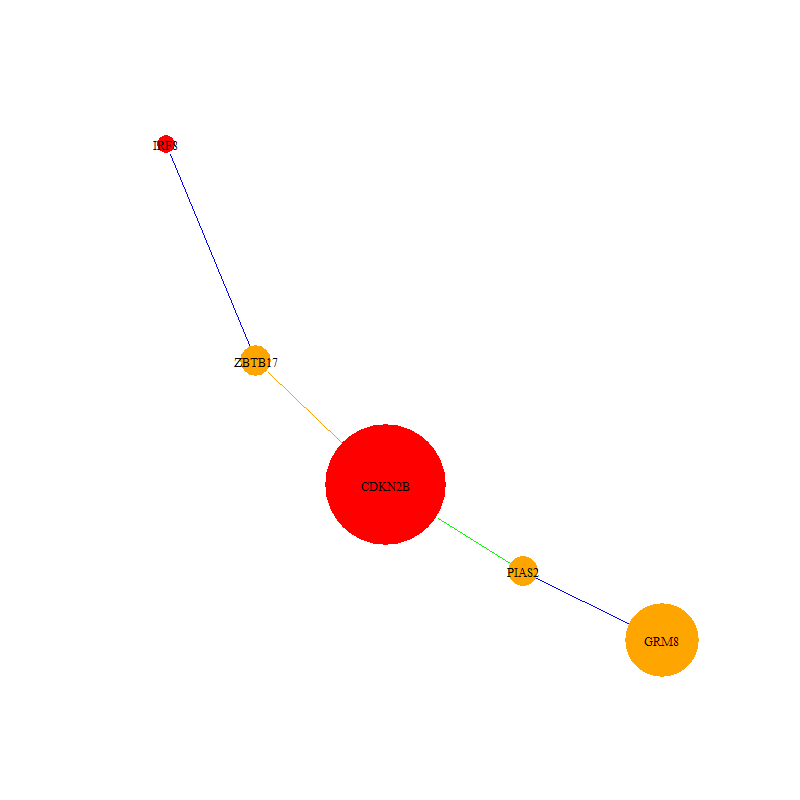

Supplement: Additional file 5 — Visualization of top ranked CCA modules. [file 1756-0381-6-17-S5.zip › CCAModules/plotCCA.14.tif]

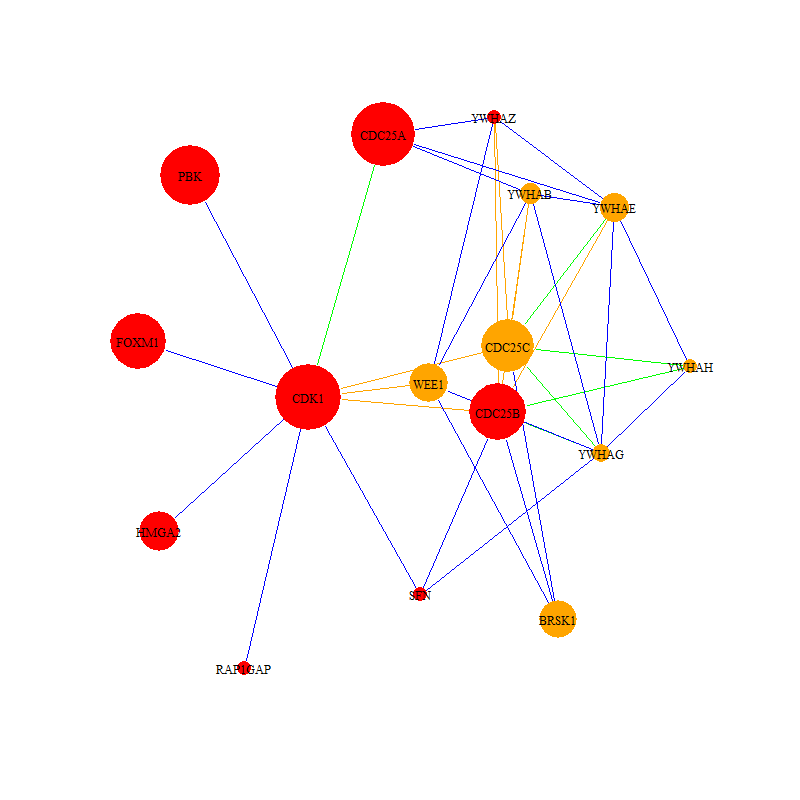

Supplement: Additional file 5 — Visualization of top ranked CCA modules. [file 1756-0381-6-17-S5.zip › CCAModules/plotCCA.144.tif]

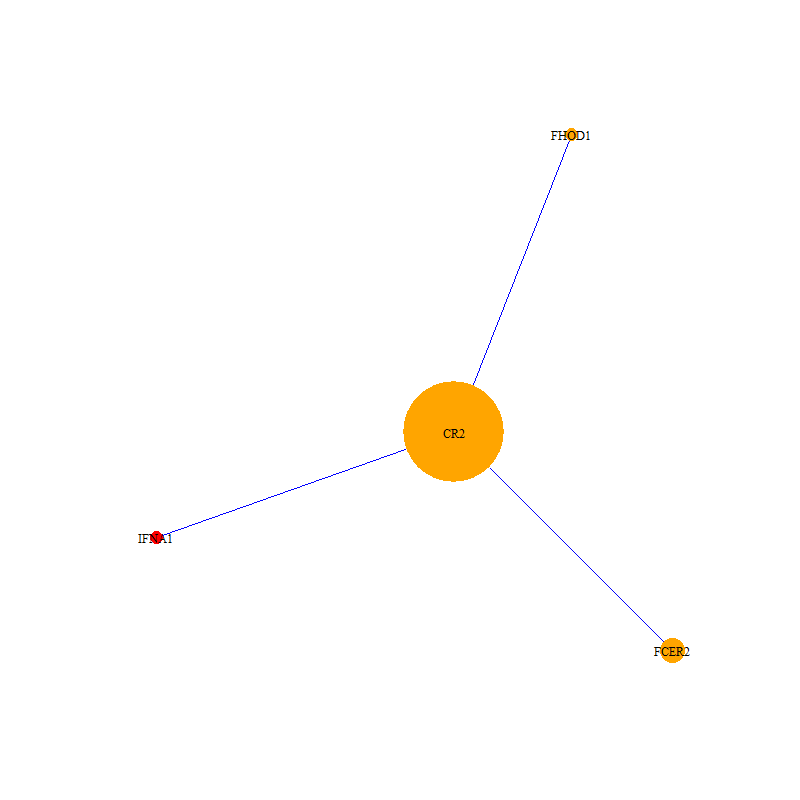

Supplement: Additional file 5 — Visualization of top ranked CCA modules. [file 1756-0381-6-17-S5.zip › CCAModules/plotCCA.156.tif]

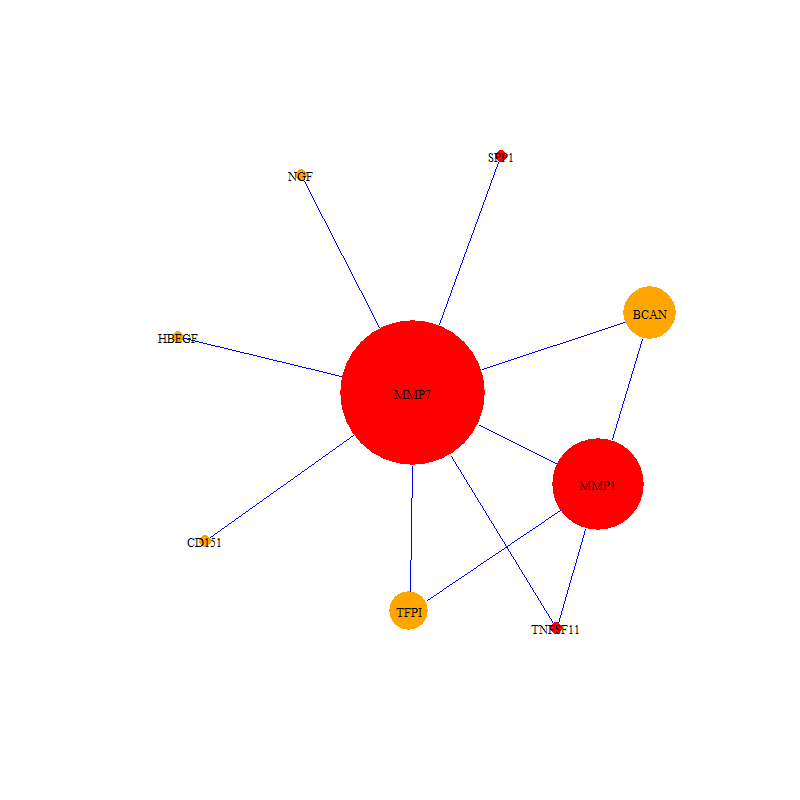

Supplement: Additional file 5 — Visualization of top ranked CCA modules. [file 1756-0381-6-17-S5.zip › CCAModules/plotCCA.158.tif]

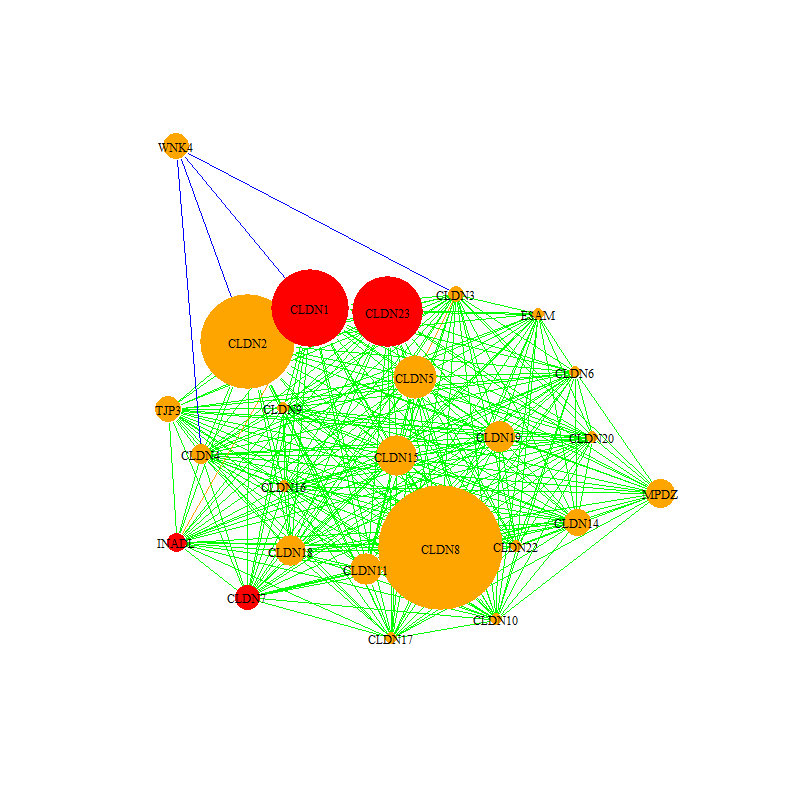

Supplement: Additional file 5 — Visualization of top ranked CCA modules. [file 1756-0381-6-17-S5.zip › CCAModules/plotCCA.182.tif]

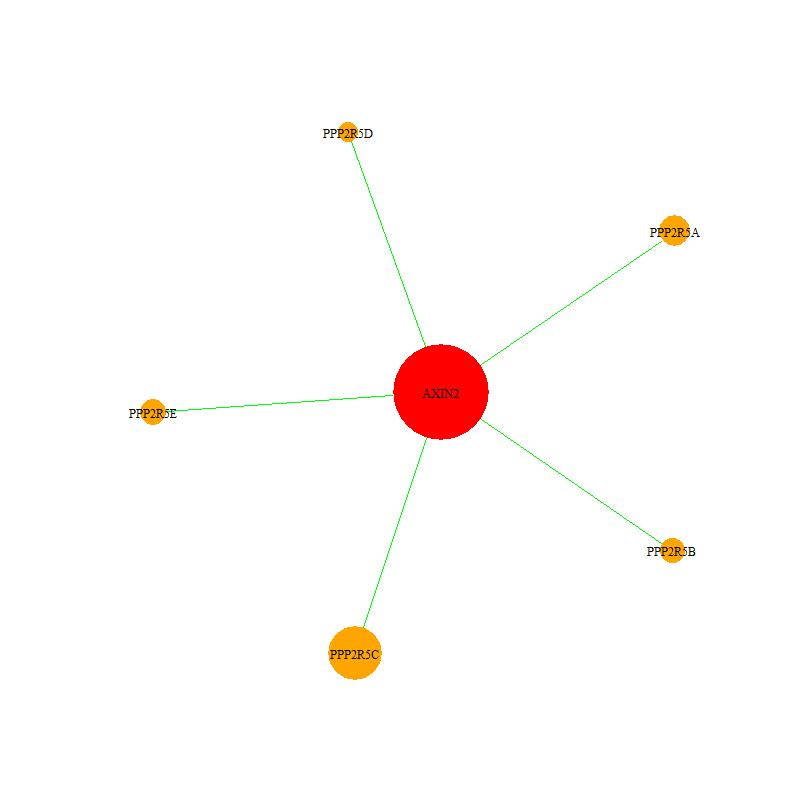

Supplement: Additional file 5 — Visualization of top ranked CCA modules. [file 1756-0381-6-17-S5.zip › CCAModules/plotCCA.183.tif]

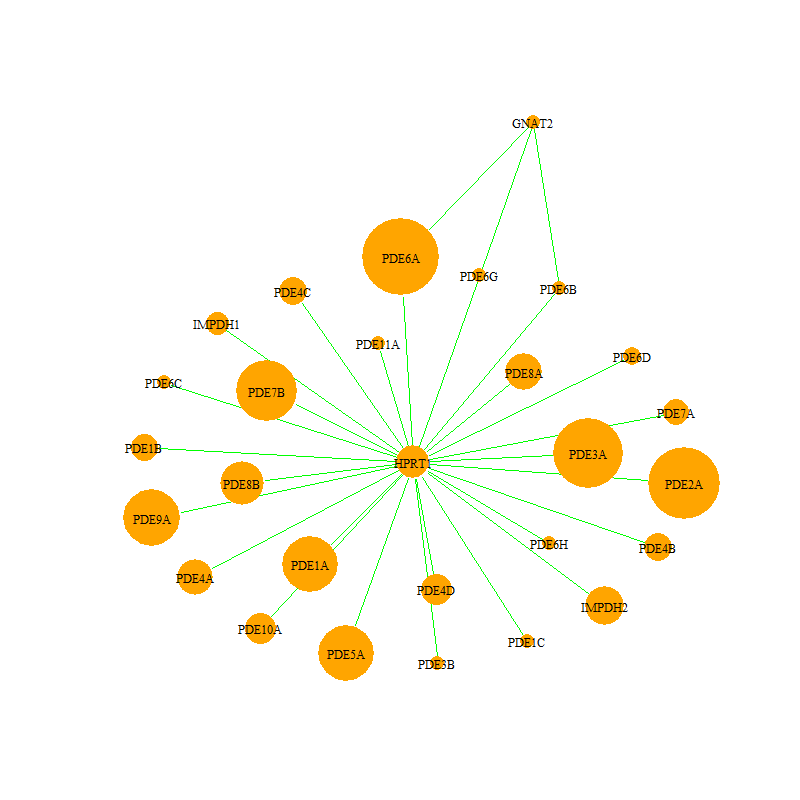

Supplement: Additional file 5 — Visualization of top ranked CCA modules. [file 1756-0381-6-17-S5.zip › CCAModules/plotCCA.240.tif]

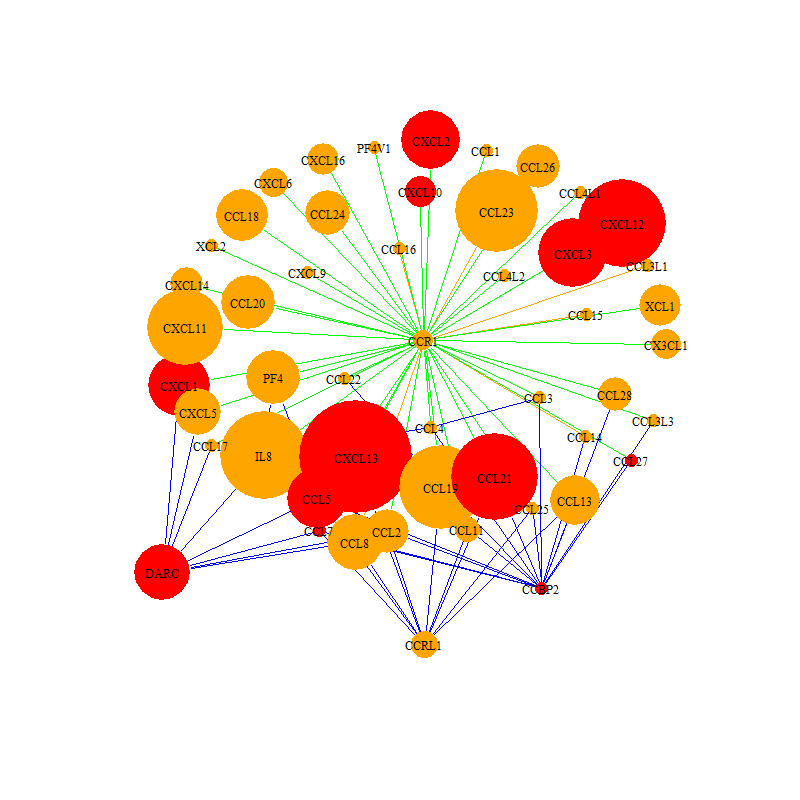

Supplement: Additional file 5 — Visualization of top ranked CCA modules. [file 1756-0381-6-17-S5.zip › CCAModules/plotCCA.257.tif]

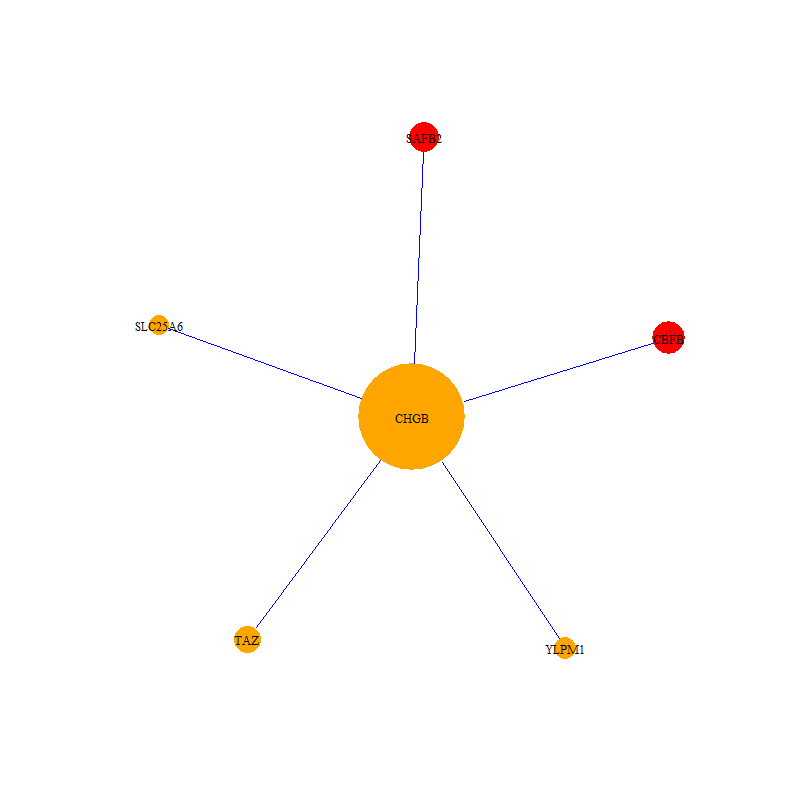

Supplement: Additional file 5 — Visualization of top ranked CCA modules. [file 1756-0381-6-17-S5.zip › CCAModules/plotCCA.267.tif]

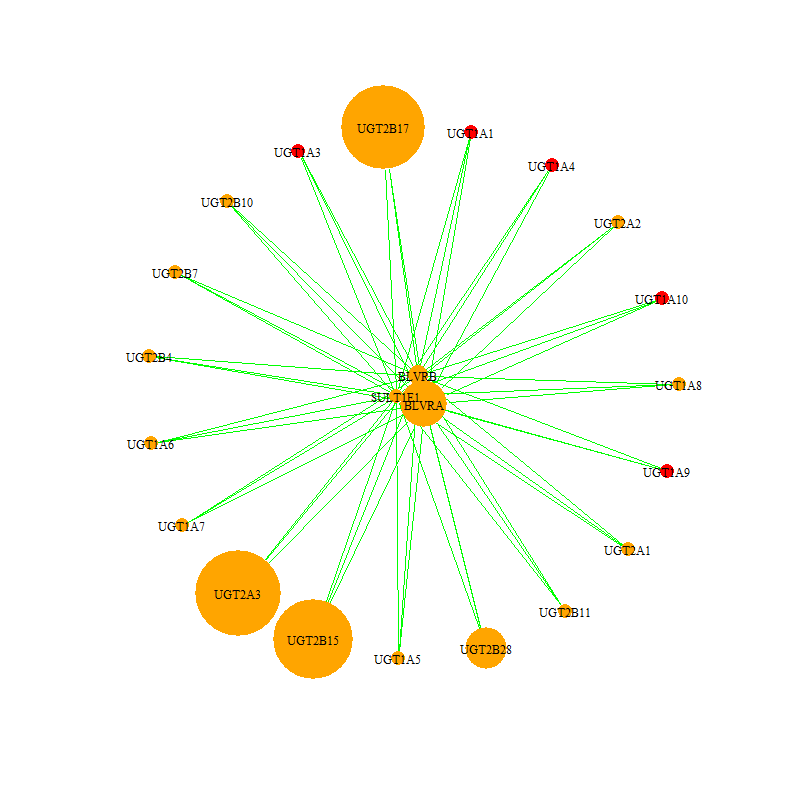

Supplement: Additional file 5 — Visualization of top ranked CCA modules. [file 1756-0381-6-17-S5.zip › CCAModules/plotCCA.290.tif]

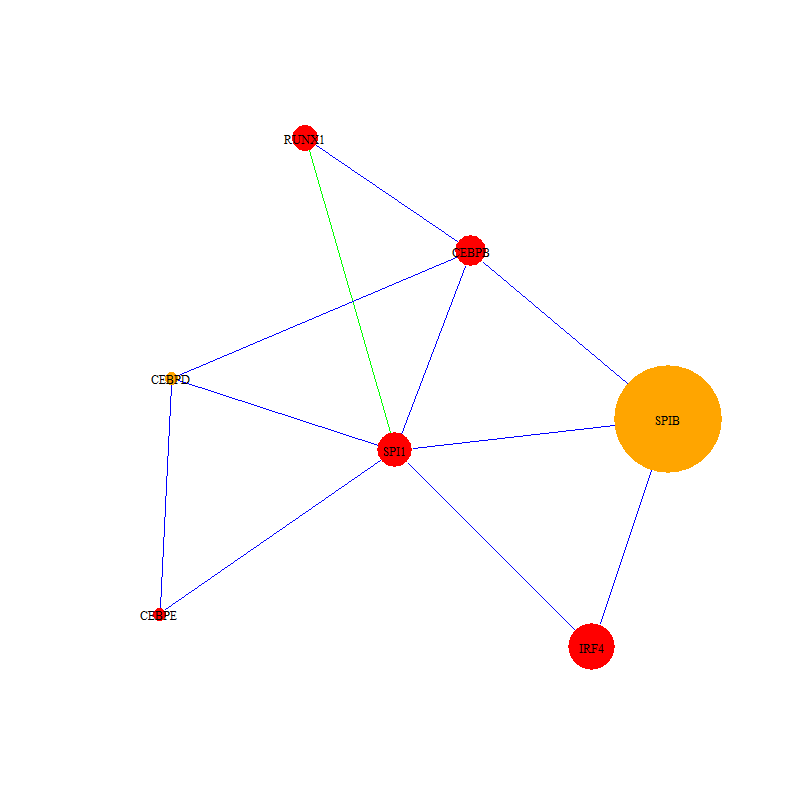

Supplement: Additional file 5 — Visualization of top ranked CCA modules. [file 1756-0381-6-17-S5.zip › CCAModules/plotCCA.301.tif]

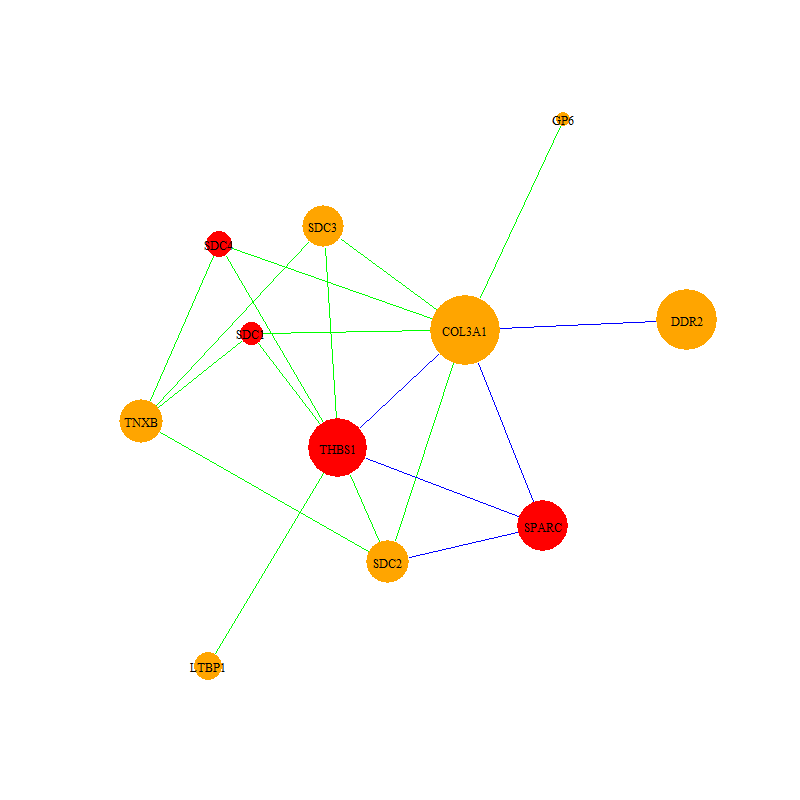

Supplement: Additional file 5 — Visualization of top ranked CCA modules. [file 1756-0381-6-17-S5.zip › CCAModules/plotCCA.328.tif]

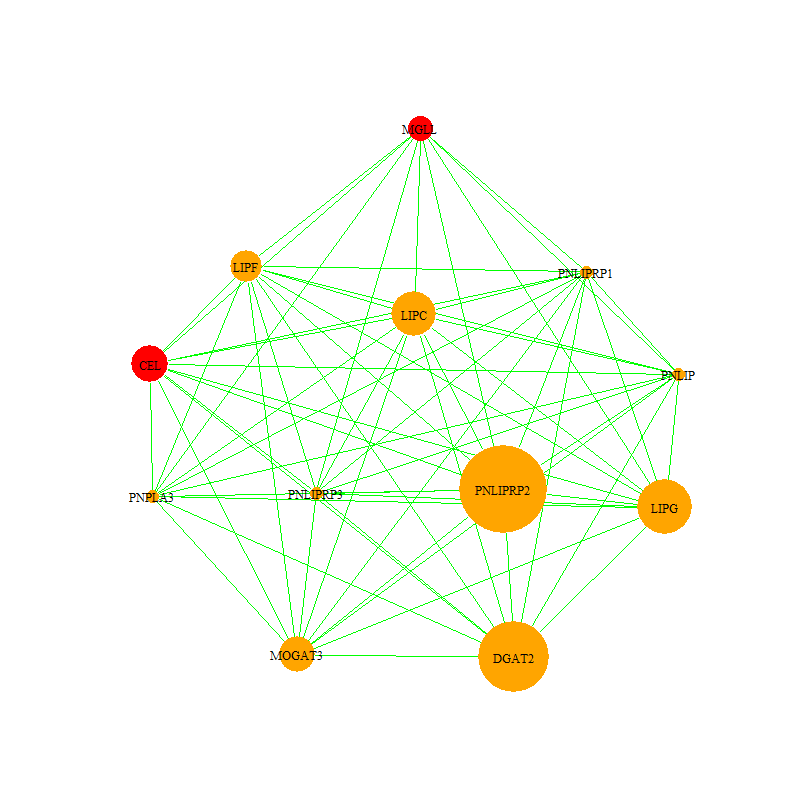

Supplement: Additional file 5 — Visualization of top ranked CCA modules. [file 1756-0381-6-17-S5.zip › CCAModules/plotCCA.334.tif]

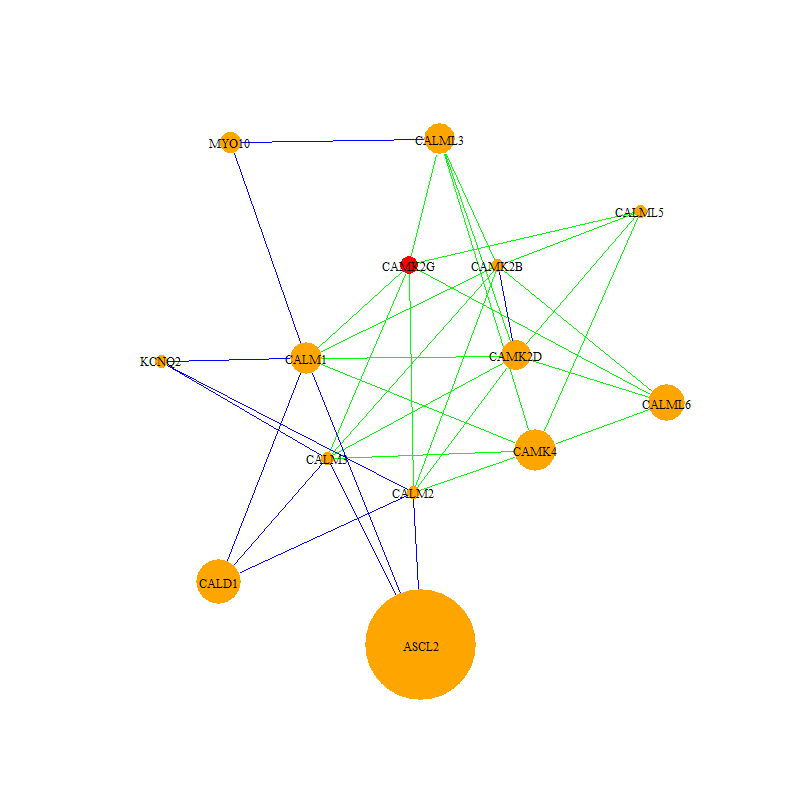

Supplement: Additional file 5 — Visualization of top ranked CCA modules. [file 1756-0381-6-17-S5.zip › CCAModules/plotCCA.345.tif]

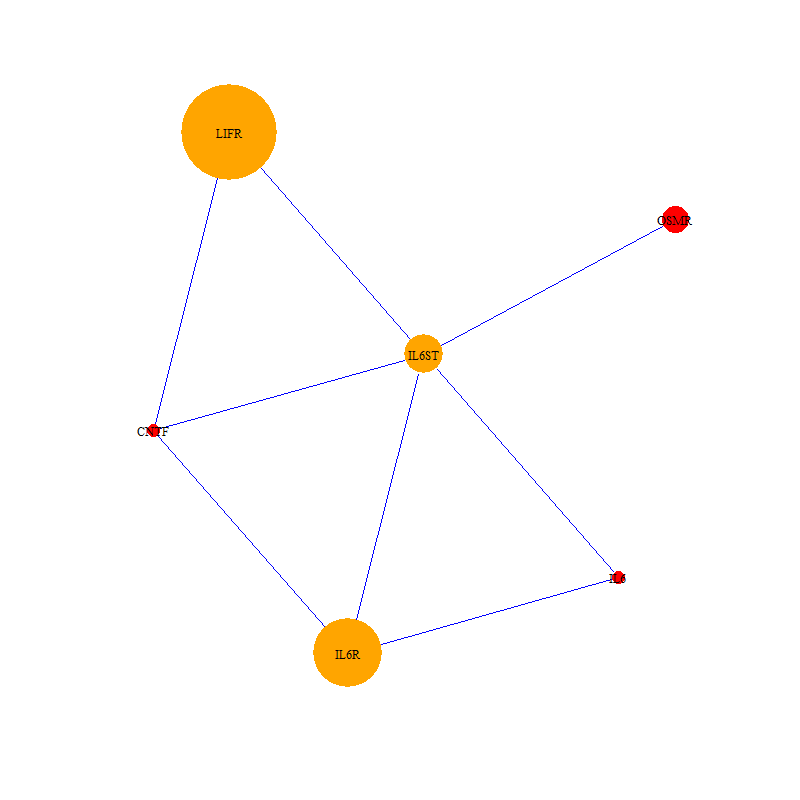

Supplement: Additional file 5 — Visualization of top ranked CCA modules. [file 1756-0381-6-17-S5.zip › CCAModules/plotCCA.410.tif]

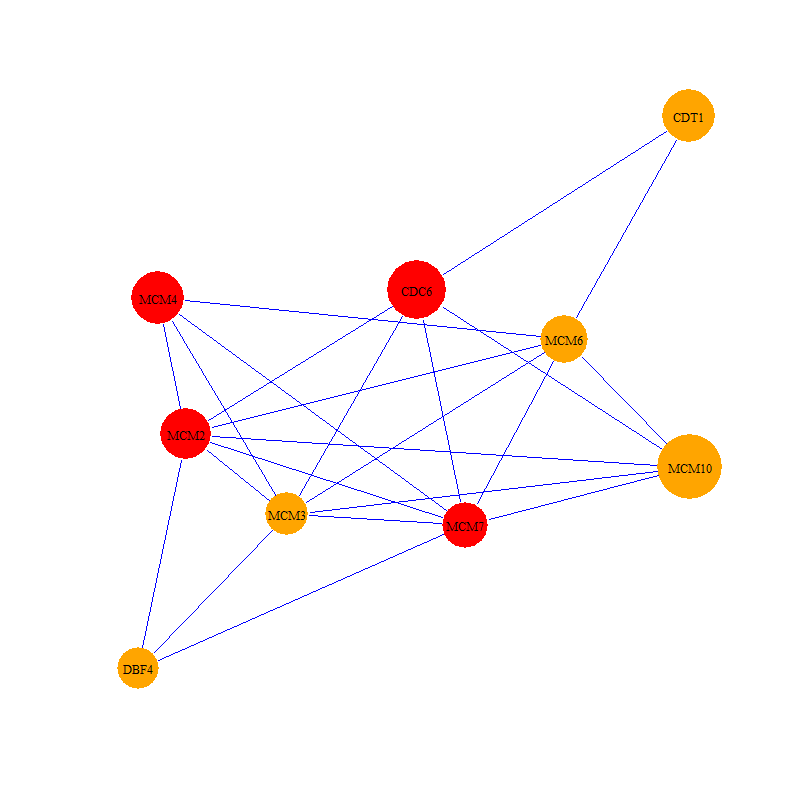

Supplement: Additional file 5 — Visualization of top ranked CCA modules. [file 1756-0381-6-17-S5.zip › CCAModules/plotCCA.412.tif]

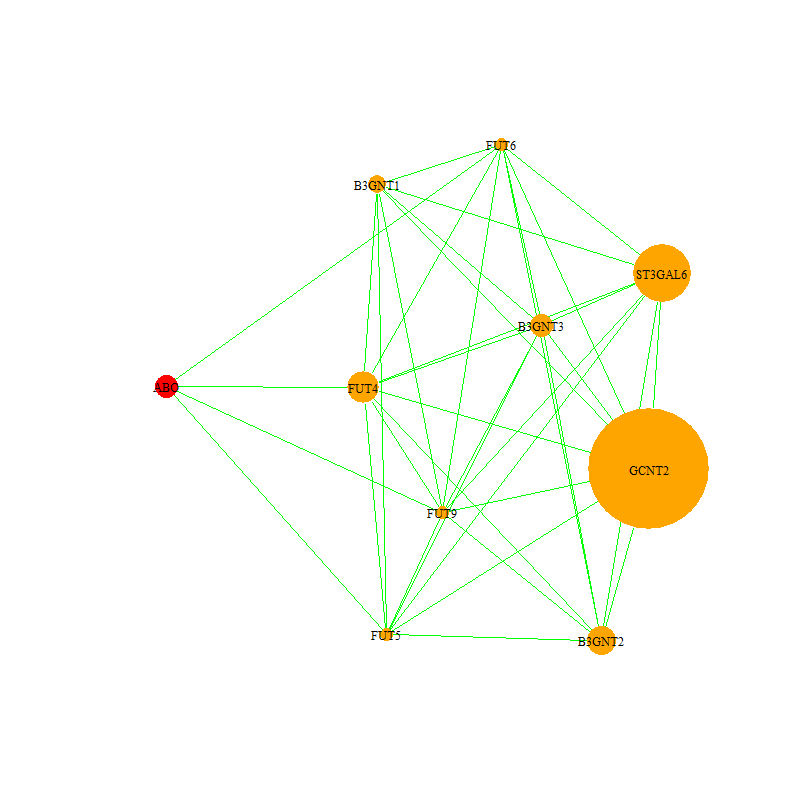

Supplement: Additional file 5 — Visualization of top ranked CCA modules. [file 1756-0381-6-17-S5.zip › CCAModules/plotCCA.452.tif]

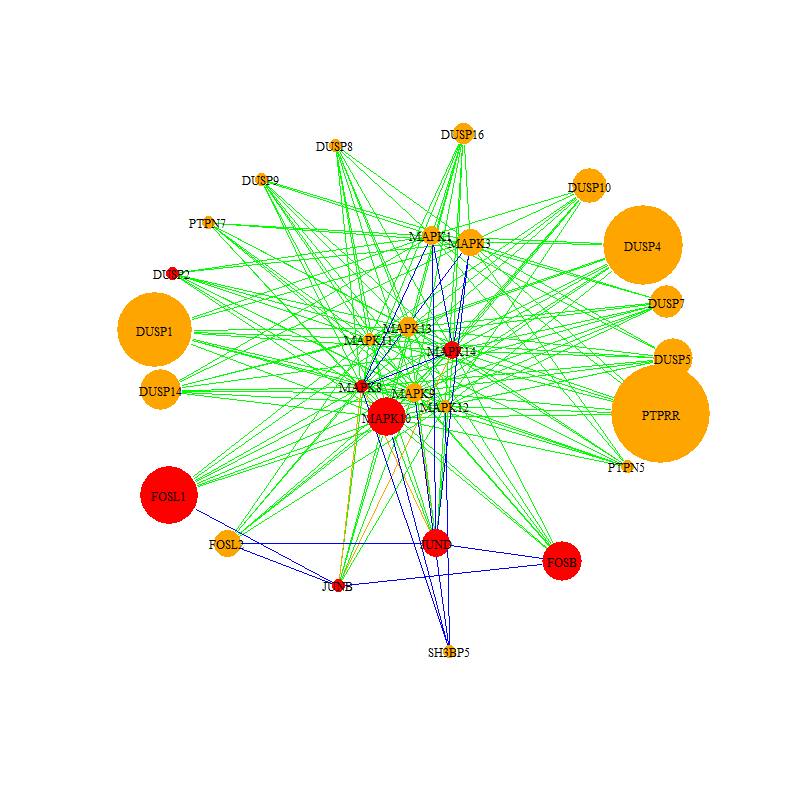

Supplement: Additional file 5 — Visualization of top ranked CCA modules. [file 1756-0381-6-17-S5.zip › CCAModules/plotCCA.487.tif]

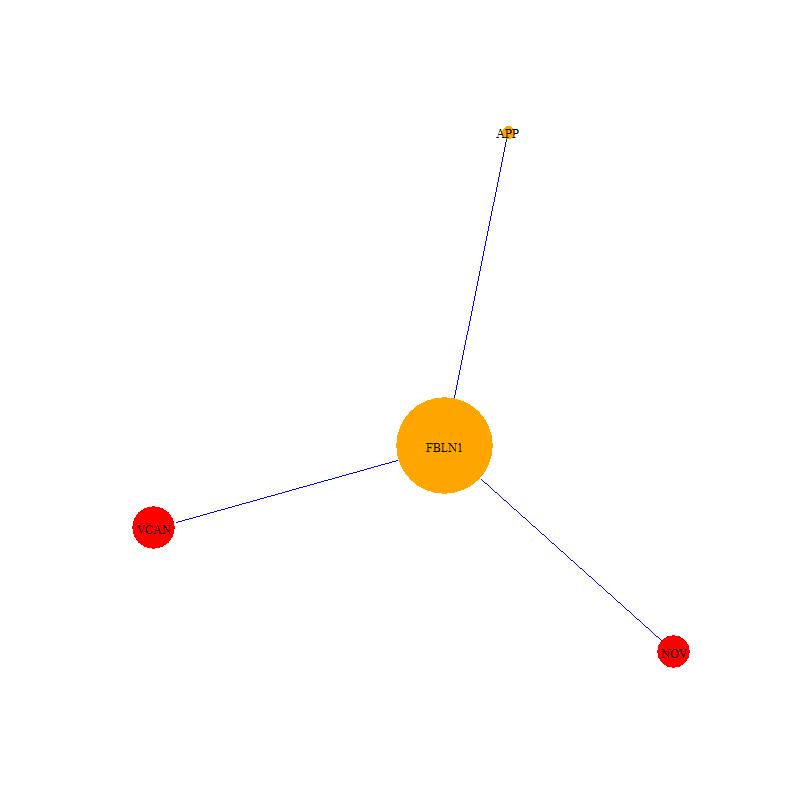

Supplement: Additional file 5 — Visualization of top ranked CCA modules. [file 1756-0381-6-17-S5.zip › CCAModules/plotCCA.54.tif]

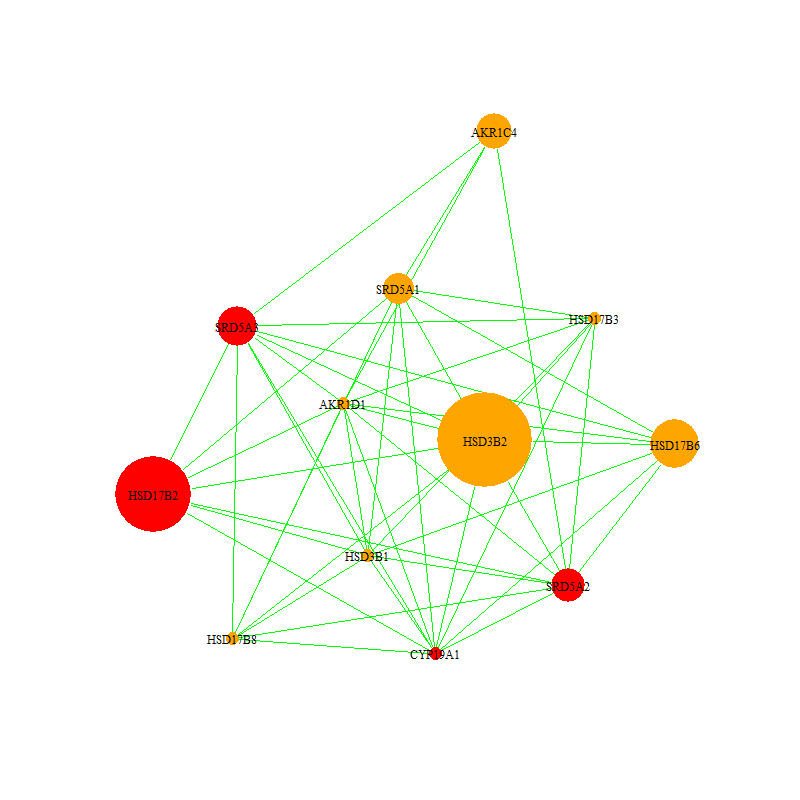

Supplement: Additional file 5 — Visualization of top ranked CCA modules. [file 1756-0381-6-17-S5.zip › CCAModules/plotCCA.757.tif]

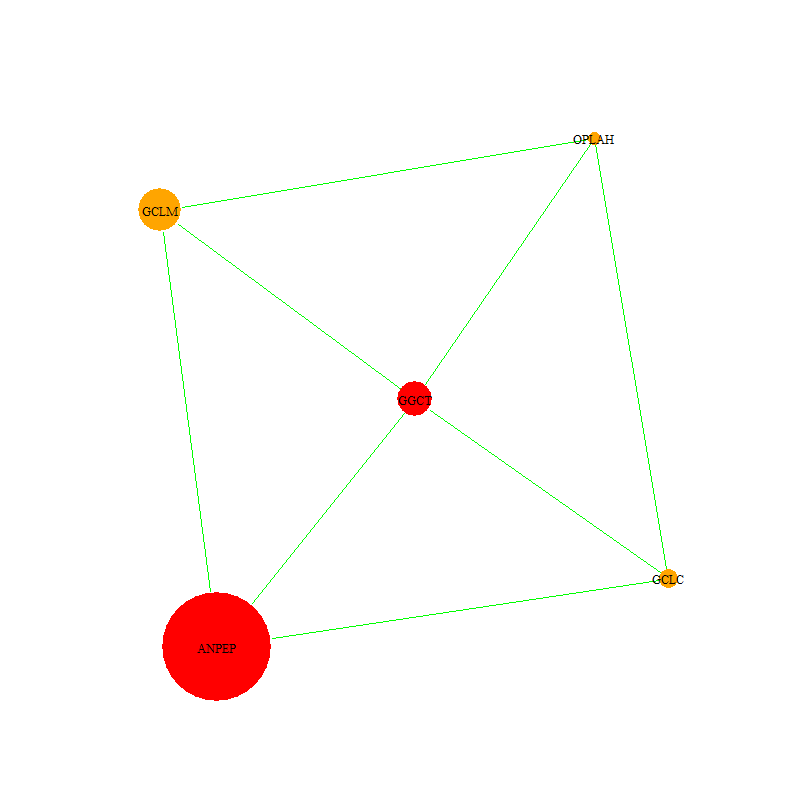

Supplement: Additional file 5 — Visualization of top ranked CCA modules. [file 1756-0381-6-17-S5.zip › CCAModules/plotCCA.758.tif]

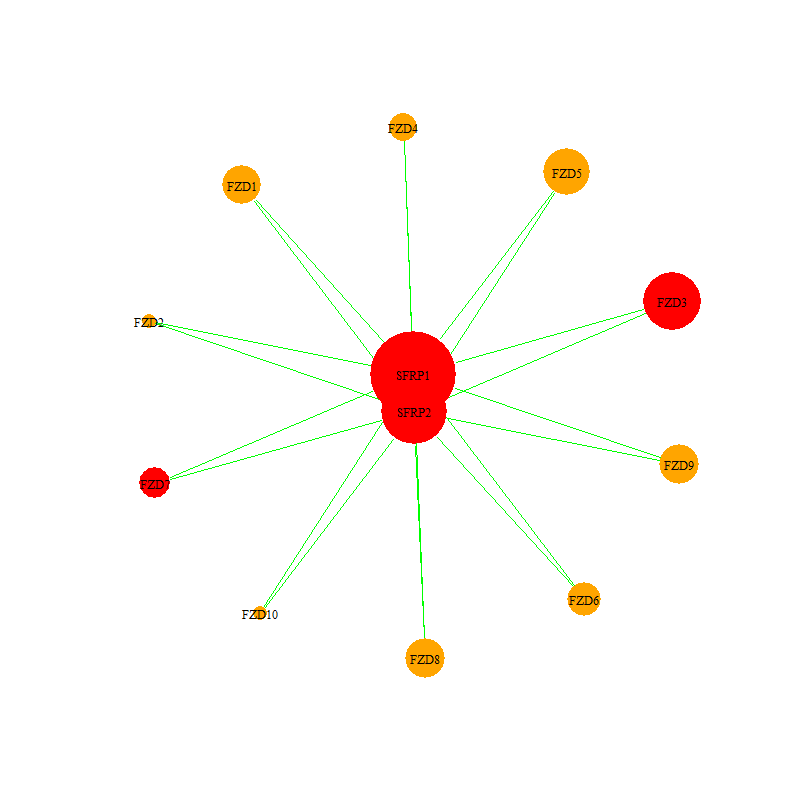

Supplement: Additional file 5 — Visualization of top ranked CCA modules. [file 1756-0381-6-17-S5.zip › CCAModules/plotCCA.762.tif]

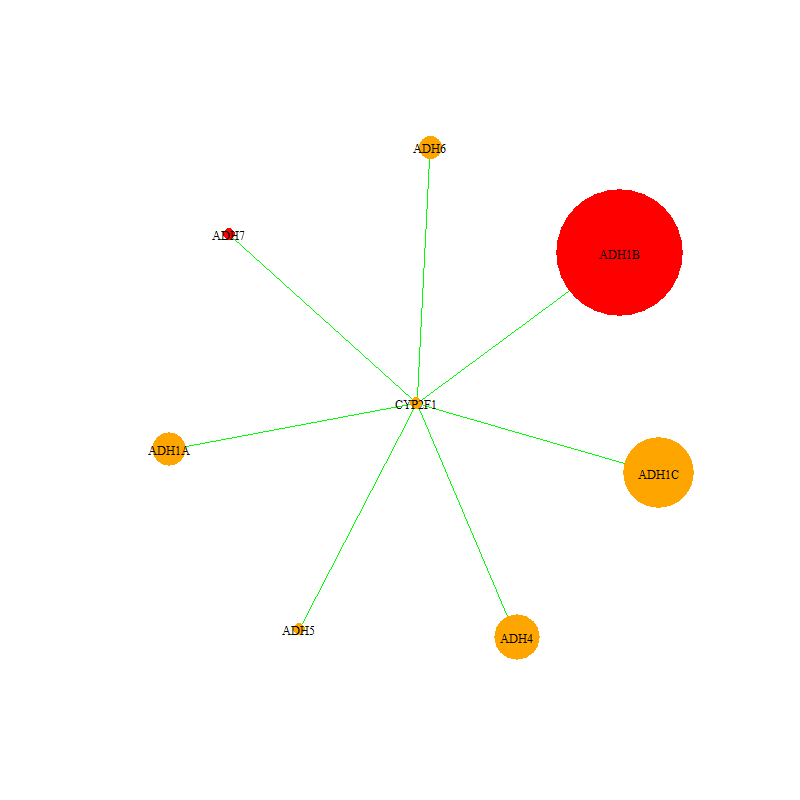

Supplement: Additional file 5 — Visualization of top ranked CCA modules. [file 1756-0381-6-17-S5.zip › CCAModules/plotCCA.770.tif]
