# Supplementary material for: Using random walks to identify cancer-associated modules in expression data
Source: BioData Min. 2013 Oct 15;6:17. doi: 10.1186/1756-0381-6-17 (PMC4015830; doi:10.1186/1756-0381-6-17)
Supplement: Additional file 6 — Key genes described in BC, HCC and CCA modules. [file 1756-0381-6-17-S6.docx]

Key Genes described in BC Modules

| Gene | Gene Description | Module | Function |
| --- | --- | --- | --- |
| SKP2 | S-phase kinase-associated protein 2 | 143 | Mediates the ubiquitination and subsequent proteasomal degradation of target proteins involved in cell cycle progression, signal transduction and transcription |
| CCNA2 | cyclin A2 | 143 | Essential for the control of the cell cycle at the G1/S (start) and the G2/M (mitosis) transitions |
| BRCA2 | breast cancer 2, early onset | 143 | Involved in double-strand break repair and/or homologous recombination. |
| CDK2 | cyclin-dependent kinase 2 | 143 | Serine/threonine-protein kinase involved in the control of the cell cycle; essential for meiosis. |
| JAK1 | Janus kinase 1 | 79 | Tyrosine kinase, involved in the IFN-alpha/beta/gamma signal pathway. Kinase partner for the interleukin (IL)-2 receptor |
| SOCS1,-2,-3 | suppressor of cytokine signaling1,-2,-3 | 79 | SOCS family proteins form part of a classical negative feedback system that regulates cytokine signal transduction, involved in negative regulation of cytokines that signal through the JAK/STAT pathway. |
| IL21R | interleukin 21 receptor | 79 | Transduces the growth promoting signal of IL21, and is important for the proliferation and differentiation of T cells, B cells, and natural killer (NK) cells. The ligand binding of this receptor leads to the activation of multiple downstream signaling molecules, including JAK1, JAK3, STAT1, and STAT3. |
| CBLC | Cbl proto-oncogene | 79 | Regulator of EGFR mediated signal transduction |
| FIGF | c-fos induced growth factor (VEGF D) | 82 | Growth factor active in angiogenesis, lymphangiogenesis and endothelial cell growth, stimulating their proliferation and migration and also has effects on the permeability of blood vessels. |
| IFGIR | insulin-like growth factor 1 receptor | 82 | Receptor tyrosine kinase which mediates actions of insulin-like growth factor 1 (IGF1). The activated IGF1R is involved in cell growth and survival control. IGF1R is crucial for tumor transformation and survival of malignant cells. |
| PDGFRA | platelet-derived growth factor receptor, alpha polypeptide | 82 | Tyrosine-protein kinase that acts as a cell-surface receptor for PDGFA, PDGFB and PDGFC and plays an essential role in the regulation of embryonic development, cell proliferation, survival and chemotaxis. |
| EGFR | epidermal growth factor receptor | 82 | Receptor tyrosine kinase binding ligands of the EGF family and activating several signaling cascades. Binding of EFGR to a ligand induces receptor dimerization and tyrosine autophosphorylation leads to cell proliferation. |
| MET | met proto-oncogene (hepatocyte growth factor receptor) | 82 | Receptor tyrosine kinase that transduces signals from the extracellular matrix into the cytoplasm by binding to hepatocyte growth factor/HGF ligand. Regulates many physiological processes including proliferation, morphogenesis and survival. |
| ErbB4 | v-erb-a erythroblastic leukemia viral oncogene homolog 4 | 82 | Tyrosine-protein kinase that plays an essential role as cell surface receptor for neuregulins and EGF family members and regulates organ development, gene transcription, cell proliferation, differentiation, migration and apoptosis. |
| IRS2 | insulin receptor substrate 2 | 82 | Mediates the control of various cellular processes by insulin |
| FGF7 | fibroblast growth factor | 82 | Plays an important role in the regulation of embryonic development, cell proliferation and differentiation. |
| VEGFA | vascular endothelial growth factor A | 82 | This gene encodes a member of the PDGF (platelet-derived growth factor)/VEGF (vascular endothelial growth factor) family. Growth factor active in angiogenesis, vasculogenesis and endothelial cell growth. Induces endothelial cell proliferation, promotes cell migration, inhibits apoptosis and induces permeabilization of blood vessels. Binds to FLT1/VEGFR1 and KDR/VEGFR2 receptors, heparan sulfate and heparin. |
| INSRR | insulin receptor-related receptor | 82 | Receptor with tyrosine-protein kinase activity. Activates a signaling pathway that involves IRS1 and AKT1/PKB |
| INSR | insulin receptor | 82 | Binding of insulin to the insulin receptor (INSR) stimulates glucose uptake . Many tumors have altered expression of IGF1R and its ligands and this constitutes an early, possible initiating, event in tumorigenesis. |
| PDGFRB | platelet-derived growth factor receptor, beta polypeptide | 82 | Tyrosine-protein kinase that acts as cell-surface receptor for PDGFB, PDGFD and PDGFA. Plays an essential role in the regulation of embryonic development, cell proliferation, survival, differentiation, chemotaxis and migration. |

Key Genes described in HCC Modules

| Gene | Gene Description | Module | Function |
| --- | --- | --- | --- |
| CDC7 | cell division cycle 7 homolog | 361 | Phosphorylates substrates that regulate the G1/S phase transition and DNA replication, including MCM2 and MCM3. |
| DBF4 | DBF4 homolog | 361 | Regulatory subunit for CDC7 which activates its kinase activity thereby playing a central role in DNA replication and cell proliferation. Required for progression of S phase. The complex CDC7-DBF4 selectively phosphorylates MCM2 and is then involved in regulating the initiation of DNA replication during cell cycle |
| ORC5L | origin recognition complex, subunit 5 | 361 | The origin recognition complex (ORC) is a highly conserved protein complex essential for the initiation of the DNA replication in eukaryotic cells. Studies in yeast demonstrated that ORC binds specifically to origins of replication and serves as a platform for the assembly of additional initiation factors such as Cdc6 and Mcm proteins. |
| CDC6 | cell division cycle 6 homolog | 361 | Involved in the initiation of DNA replication and s in checkpoint controls that ensure complete DNA replication before mitosis. Reported to be regulated in response to mitogenic signals and transcriptional control involving E2F proteins. |
| MCM2,-3,-4,-5,-7 | minichromosome maintenance complex component 2,-3,-4,-5,-7 | 361 | The MCM2-7 complex (MCM complex) is the putative replicative helicase essential for 'once per cell cycle' DNA replication initiation and elongation in eukaryotic cells. Required for DNA replication and cell proliferation |
| IGF1 | insulin-like growth factor 1 (somatomedin C) | 429 | The insulin-like growth factors are structurally and functionally related to insulin but have a much higher growth-promoting activity. |
| IDE | insulin-degrading enzyme | 429 | Plays a role in the cellular breakdown of insulin, IAPP, glucagon, bradykinin, kallidin and other peptides, and thereby plays a role in intercellular peptide signaling. |
| NOV | nephroblastoma overexpressed | 429 | Immediate-early protein likely to play a role in cell growth regulation |
| IGFBP7 | insulin-like growth factor binding 7 | 429 | Binds IGF-I and IGF-II with low affinity. Stimulates prostacyclin (PGI2) production and cell adhesion. |
| RPS6KA2 | ribosomal protein S6 kinase, 90kDa, polypeptide 2 | 429 | Serine/threonine-protein kinase that acts downstream of ERK (MAPK1/ERK2 and MAPK3/ERK1) signaling and mediates mitogenic and stress-induced activation of transcription factors, regulates translation, and mediates cellular proliferation, survival, and differentiation. May function as tumor suppressor in epithelial ovarian cancer cells. |
| RPS6KA6 | ribosomal protein S6 kinase, 90kDa, polypeptide 6 | 429 | Constitutively active serine/threonine-protein kinase that exhibits growth-factor-independent kinase activity. Participates in p53/TP53-dependent cell growth arrest signaling and plays an inhibitory role during embryogenesis |
| DUSP1,-2,-6,-9 | dual specificity phosphatase 1, -2,-6,-9 | 414 | These phosphatases inactivate their target kinases by dephosphorylation. They negatively regulate members of the MAP- kinase superfamily (MAPK/ERK, SAPK/JNK, p38), which are associated with cellular proliferation and differentiation. |
| MAPK9,-10,-12,-14 | mitogen-activated protein kinase 9,-10,-12,-14 | 414 | MAP kinases act as an integration point for multiple biochemical signals, and are involved in a wide variety of cellular processes such as proliferation, differentiation, transcription regulation and development. |
| PTPRR | protein tyrosine phosphatase, receptor type, R | 414 | PTPs are signaling molecules that regulate a variety of cellular processes including cell growth, differentiation, mitotic cycle, and oncogenic transformation. Silencing of this gene has been associated with colorectal cancer. Sequesters mitogen-activated protein kinases (MAPKs) such as MAPK1, MAPK3 and MAPK14 in the cytoplasm in an inactive form. |
| FOSL1 | FOS-like antigen, FBJ murine osteosarcoma viral oncogene B | 414 | Fos proteins interact with Jun proteins enhancing their DNA binding activity. FOS proteins have been implicated as regulators of cell proliferation, differentiation, and transformation. |
| RIPK2 | receptor-interacting serine-threonine kinase 2 | 414 | Serine/threonine/tyrosine kinase that plays an essential role in modulation of innate and adaptive immune responses. It is a potent activator of NF-kappaB and inducer of apoptosis in response to various stimuli. |
| SH3BP5 | SH3-domain binding protein 5 | 414 | Plays a negative regulatory role in BTK-related signaling in B-cells. May be involved in BCR-induced apoptotic cell death. |
| JUNB | jun B proto-oncogene | 414 | Transcription factor involved in regulating gene activity following the primary growth factor response. |

Key Genes described in CCA Modules

| Gene | Gene Description | Module | Function |
| --- | --- | --- | --- |
| SPI1 | spleen focus forming virus (SFFV) proviral integration oncogene | 301 | Binds to the PU-box, a purine-rich DNA sequence that can act as a lymphoid-specific enhancer. A transcriptional activator involved in the differentiation or activation of macrophages or B-cells. Binds RNA and modulates pre-mRNA splicing. |
| SPIB | Spi-B transcription factor | 301 | Transcriptional activator which binds to the PU-box, a purine-rich DNA sequence that can act as a lymphoid-specific enhancer. Required for B-cell receptor (BCR) signaling, necessary for normal B-cell development and antigenic stimulation |
| RUNX1 | runt-related transcription factor 1 | 301 | Core binding factor (CBF) is a transcription factor that binds to many enhancers and promoters and is involved in normal hematopoiesis development. Chromosomal translocations are well-documented and are associated with leukemia. |
| IRF4 | interferon regulatory factor 4 | 301 | A member of the IRF (interferon regulatory factor) family of transcription factors, important in the regulation of interferons in response to infection by virus, and in the regulation of interferon-inducible genes. IRF4 negatively regulates Toll-like-receptor (TLR) signaling. A translocation involving this gene and the IgH may be a cause of multiple myeloma. |
| CEBPB | CCAAT/enhancer binding protein (C/EBP), beta | 301 | Transcriptional activator in the regulation of genes involved in immune and inflammatory responses. Binds to an IL-1 response element in the IL-6 gene and plays a role in regulation of acute-phase reaction, inflammation and hemopoiesis. |
| CDK1 | cyclin-dependent kinase 1 | 144 | A member of the Ser/Thr protein kinase family that acts as a catalytic subunit of the protein kinase complex known as M-phase promoting factor (MPF), which is essential for G1/S and G2/M phase transitions of eukaryotic cell cycle. |
| PBK | PDZ binding kinase | 144 | Phosphorylates MAP kinase p38 and may be active only in mitosis. Can form a complex with TP53, leading to TP53 destabilization and attenuation of G2/M checkpoint in response to DNA damage. |
| HMGA2 | high mobility group AT-hook 2 | 144 | A transcriptional regulator that plays an key role in the meiotic G2/M transition and in cell cycle regulation via CCNA2. |
| FOXM1 | forkhead box M1 | 144 | Transcriptional factor regulating the expression of cell cycle genes essential for DNA replication and mitosis. |
| BRSK1 | BR serine/threonine kinase 1 | 144 | Serine/threonine-protein kinase that plays a key role in neuron polarization and centrosome duplication. Phosphorylates CDC25B, CDC25C, MAPT/TAU, RIMS1, TUBG1, TUBG2 and WEE1. Involved in the DNA damage checkpoint, probably by inhibiting CDK1 activity through phosphorylation and activation of WEE1, and inhibition of CDC25B and CDC25C. |
| WEE1 | WEE1 homolog | 144 | A nuclear tyrosine kinase belonging to the Ser/Thr family of protein kinases. Catalyzes the inhibitory tyrosine phosphorylation of CDC2/cyclin B kinase, and appears to coordinate the transition between DNA replication and mitosis. |
| CDC25A | cell division cycle 25 homolog A | 144 | Tyrosine protein phosphatases and is required for progression from G1 to S phase of the cell cycle. It dephosphorylates CDK1 and CDK2 and it is involved in the DNA damage response. It has oncogenic properties that are not well-understood. |
| CDC25B | cell division cycle 25 homolog B | 144 | Tyrosine protein phosphatase required for G2/M phases of the cell cycle progression and abscission during cytokinesis. Dephosphorylates CDK1 and stimulates its kinase activity. CDC25B has oncogenic properties that are not well-understood. |
| CDC25C | cell division cycle 25 homolog C | 144 | Tyrosine protein phosphatase required for progression of the cell cycle by activating G2 cells into prophase. Directly dephosphorylates CDK1 and activates its kinase activity. It is also thought to suppress p53-induced growth arrest. |
| YWHAB, -E | tyrosine 3-monooxygenase /tryptophan 5-monooxygenase activation protein, beta, -epsilon | 144 | The 14-3-3 family of proteins interacts with CDC25 phosphatases, RAF1 and IRS1 proteins, suggesting a role in biochemical activities related to signal transduction, such as cell division, mitogenic signaling and regulation of insulin sensitivity. YWHAE has been implicated in the pathogenesis of small cell lung cancer. |
| RAP1GAP | RAP1 GTPase activating protein | 144 | T GTPase-activating-protein (GAP) that down-regulates activity of the ras-related RAP1 protein. RAP1 plays a role in diverse processes such as cell proliferation, adhesion, differentiation, and embryogenesis. |
| SFRP1,-2 | secreted frizzled-related protein 1-2 | 762 | Soluble frizzled-related proteins (sFRPS)are modulators of Wnts and Wnt signaling. They regulate differentiation and cell growth. Epigenetic silencing of SFRP genes leads to deregulation of the Wnt-pathway which is associated with cancer. |
| FZD2,-3,-5,-6, -8,-9 | frizzled family receptor 2,-3,-5,-6, -8,-9 | 762 | Most Frizzled receptors are coupled to the beta-catenin canonical signaling pathway, which leads to the activation of disheveled proteins, inhibition of GSK-3 kinase, nuclear accumulation of beta-catenin and activation of Wnt target genes. |
